# Covalent cucurbit[7]uril–dye conjugates for sensing in aqueous saline media and biofluids†

**DOI:** 10.1039/d0sc03079a

**Published:** 2020-09-22

**Authors:** Changming Hu, Laura Grimm, Amrutha Prabodh, Ananya Baksi, Alicja Siennicka, Pavel A. Levkin, Manfred M. Kappes, Frank Biedermann

**Affiliations:** Institute of Nanotechnology (INT), Karlsruhe Institute of Technology (KIT) Hermann-von-Helmholtz Platz 1 76344 Eggenstein-Leopoldshafen Germany frank.biedermann@kit.edu; Institute of Physical Chemistry (IPC), Karlsruhe Institute of Technology (KIT) Fritz-Haber-Weg 6 76131 Karlsruhe Germany; Institute of Chemical and Biological Systems – Functional Molecular Systems (IBCS-FMS), Karlsruhe Institute of Technology (KIT) Hermann-von-Helmholtz Platz 1 76344 Eggenstein-Leopoldshafen Germany

## Abstract

Non-covalent chemosensing ensembles of cucurbit[*n*]urils (CB*n*) have been widely used in proof-of-concept sensing applications, but they are prone to disintegrate in saline media, *e.g.* biological fluids. We show here that covalent cucurbit[7]uril–indicator dye conjugates are buffer- (10× PBS buffer) and saline-stable (up to 1.4 M NaCl) and allow for selective sensing of Parkinson's drug amantadine in human urine and saliva, where the analogous non-covalent CB7⊃dye complex is dysfunctional. The in-depth analysis of the covalent host–dye conjugates in the gas-phase, and deionized *versus* saline aqueous media revealed interesting structural, thermodynamic and kinetic effects that are of general interest for the design of CB*n*-based supramolecular chemosensors and systems. This work also introduces a novel high-affinity indicator dye for CB7 through which fundamental limitations of indicator displacement assays (IDA) were exposed, namely an impractical slow equilibration time. Unlike non-covalent CB*n*⊃dye reporter pairs, the conjugate chemosensors can also operate through a S_N_2-type guest–dye exchange mechanism, which shortens assay times and opens new avenues for tailoring analyte-selectivity.

## Abstract 2

Unimolecular chemosensor shows superior stability and detection capabilities in biofluids compared to bimolecular reporter pairs.

## Graphical abstract

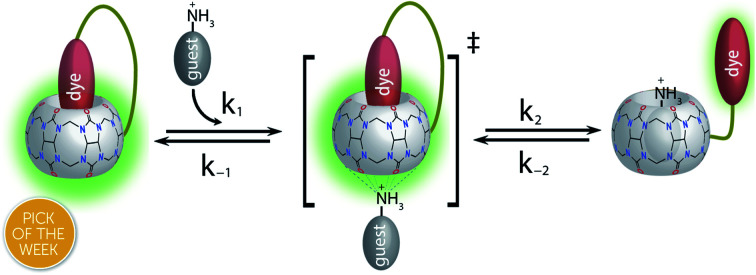

## Introduction

Emission based detection and analysis under the use of supramolecular assemblies, namely host–guest combinations, has become an extensively investigated research field in the last decades. Supramolecular complexes were widely investigated regarding their use in the fields of drug detection^1–3^ and enzymatic reaction monitoring.^4,5^ For spectroscopically silent analytes, several methods such as indicator displacement assays (IDA), where a dye is first bound to a host and then replaced by a guest, were developed.^6–10^ Among the various available host systems for analyte detection, cucurbit[*n*]urils (CB*n*), which are glycoluril-based macrocyclic hosts, have become popular building blocks for chemosensors on account of their water solubility, and their wide applicability in binding indicators and biorelevant analytes.^11–13^ Although CB*n*-based chemosensors possess advantageous high binding affinities and fast binding kinetics for many biorelevant small molecules in deionized water,^14–16^ the non-covalent interaction between host and guest is strongly modulated by salts due to competitive^17^ (or cooperative)^18^ cation binding to the carbonyl-fringed CB*n* portals. Besides, non-covalent CB*n*⊃dye reporter pairs – as any bimolecular non-covalent complex – are inherently prone to dissociate upon dilution. Therefore, many reported CB*n*-based chemosensors are operational for sensing applications in deionized water or “minimal buffers” but were often not suitable for applications in saline media or biofluids, especially if their salt concentration is varying from sample to sample (matrix effects).^19,20^ We recently demonstrated that the use of a high-affinity dye for CB8 enabled the detection of the drug memantine in blood serum by IDA.^21^ However, this approach can have severe fundamental limitations, *e.g.* for CB7-based assays, that are discussed further below.

Consequently, integrating the indicator and receptor into a single, non-dissociable unimolecular chemosensor appears to be a promising alternative design strategy to overcome dilution issues and to reduce the effect of salts on the chemosensor performance in biofluids. For instance, emissive naphthalene units were installed as cavity walls in *de novo* cucurbit[*n*]uril-derivatives,^22^ which thereby were functional in human urine for detecting addictive over-the-counter drugs.^23^

Unimolecular host–dye conjugates based on cucurbit[7]uril macrocycles would be promising chemosensors on account of CB7's exceptionally high binding affinity for many hydrophobic or cationic bioactive molecules such as steroids and polyamines,^16,24^ that can reach up to astonishing ∼10^15^ M^−1^ for adamantane derivatives.^25^ The underlying driving force for these outstanding binding affinities is the interplay of high-energy water release from inside of the cavity^26^ and hydrophobic interactions of guest and inner cavity, hydrogen bond formation and electrostatic interactions of the carbonyl-fringed rims with cationic groups on the guest.^2,27,28^

**Fig. 1:**
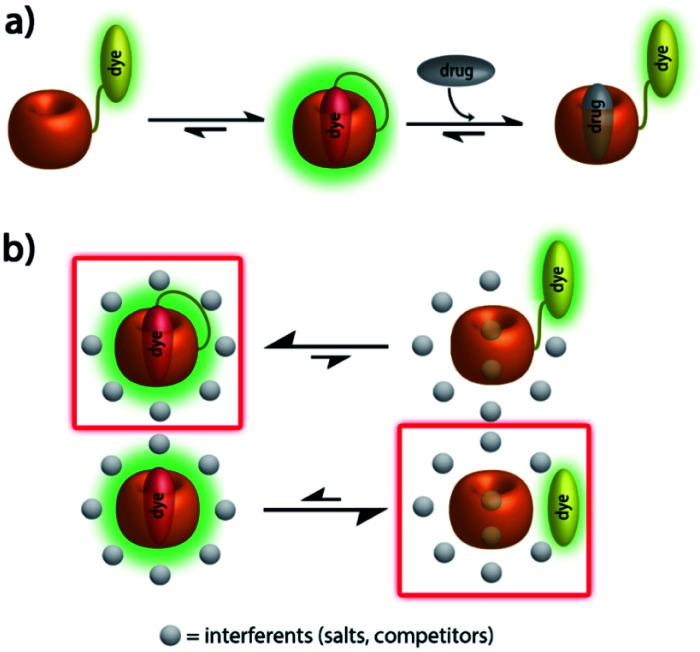
(a) Schematic representation of analyte-induced conformational changes of CB7–dye conjugates that can be detected by emission spectroscopy; (b) schematic representation of the equilibrium between free- and bound-formation of CB7–dye with/without linker in the presence of a high concentration of interferents.

Herein, we present unimolecular CB7-based chemosensors with hydrophilic and flexible linkers that allow for self-encapsulation of the indicator dye in the host cavity (Fig. 1a).

## Results

### Design and synthesis of CB7–dye conjugates

**Scheme 1:**
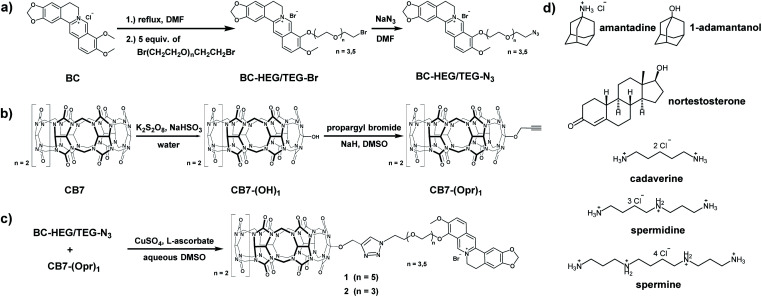
Synthesis routes of (a) berberine-HEG/TEG-azide, (b) mono-propargyl cucurbit[7]uril, (c) chemosensor **1** and **2** and (d) chemical structure of all guests.

Through synthetic advances, it is now feasible to synthesize mono-functionalized CB7 (ref. 29–34) with chemically reactive groups, that allow for the covalent attachment of dye molecules. For instance, CB7 was covalently linked to tetramethylrhodamine and the resulting conjugate showed CB7-like binding properties for typical CB7–guests.^35^ A carboxyfluorescein-labeled CB7 chemosensor was reported for DNA sensing by using Förster resonance energy transfer (FRET).^36^ Cyanine-3 (Cy3) covalently labeled CB7 (CB7-Cy3) was utilized for the monitoring of single-vesicle fusion assays,^37^ protein bioimaging,^38^ visualizations of autophagosome-lysosome fusion,^39^ and investigation of cellular uptake and excretion pathways.^40^ However, such CB7–dye conjugates were connected by short linkers which can't enable the self-encapsulation of the dye into the CB7 cavity. Consequently, the signal change upon guest binding was modest^35^ or an additional, non-covalently bound indicator dye was required,^36^ respectively. Several different fluorescent reporter dyes are known for CB7,^41^ out of which many are p*K*_a_ shift-dyes, *e.g.* acridine dyes^42^ and quinone-imine dyes.^43^ These dyes typically become protonated and thereby show enhanced emission intensity upon inclusion in the CB7 cavity that is known to stabilize positively charged species. Such p*K*_a_ shift dyes work well in buffered aqueous media of a defined pH, however, undesirable matrix-dependent signal variations are found when biological fluids of a varying pH, *e.g.* urine, are used. In this work, berberine was selected as an indicator dye because it shows a large intensity enhancement on inclusion in CB7,^44^ a high binding constant of 10^7^ M^−1^ in deionized water^45^ and is chemically functionalizable through a demethylation-alkylation procedure (Scheme 1a).^46^ Ethylene glycol-based linkers were chosen as relatively hydrophilic and flexible connectors between CB7 and berberine moieties.^47^ Chemical modeling suggested that a linker length of at least four ethylene glycol units is necessary to enable the entry into and exit of the berberine dye from the CB7 cavity. Thus, hexaethylene glycol (HEG) and tetraethylene glycol (TEG) were utilized as linkers to evaluate the effect of the linker-length.

The host–dye conjugates cucurbit[7]uril-HEG-berberine (**1**) and cucurbit[7]uril-TEG-berberine (**2**) were prepared through a convergent synthetic route shown in Scheme 1. To keep the number of synthetic steps with CB7-species to a minimum, the HEG or TEG linkers were attached to the berberine moiety, resulting in azide terminated berberine-hexaethylene glycol-azide (BC-HEG-N_3_) or berberine-tetraethylene glycol-azide (BC-TEG-N_3_) conjugates (see Scheme 1a). The conjugates are then coupled to alkyne-functionalized CB7 by a Huisgen 1,3-dipolar cycloaddition (see Scheme 1c), which was selected because of its good reactivity and lack of side products. Specifically, BC-HEG-N_3_ and BC-TEG-N_3_ were prepared through a three-step procedure from commercial berberine hydrochloride (see ESI† for details) in overall 54% yield on a 50 mg scale, and mono-propargyloxylated CB7 (CB7-(Opr)_1_) was synthesized in a two-step hydroxylation-alkylation procedure^48^ with an overall yield of 5% on a 20 mg scale. The modest yield is in accordance with the literature, but fortunately not problematic because CB7 can be readily prepared at a gram scale. Purification of the BC-HEG-N_3_ and BC-TEG-N_3_ dye-linker conjugates was performed by flash column chromatography on silica, while mono-propargyloxylated CB7 was obtained *via* mono-hydroxylated CB7 which was purified through CHP20P resin columns. Coupling of BC-HEG-N_3_ or BC-TEG-N_3_ with CB7-(Opr)_1_ was then performed in DMSO/H_2_O (v/v = 55/45) in the presence of CuSO_4_ and l-ascorbate. The purification of chemosensor **1** and **2** was carried out by HPLC on a preparative scale with a C18 column and a mixture of acetonitrile and 0.1% trifluoroacetic acid (TFA) aqueous (v/v = 1/3) as a solvent to remove unreacted starting material and catalyst.

### Characterization and conformation of CB7–dye conjugates

**Fig. 2:**
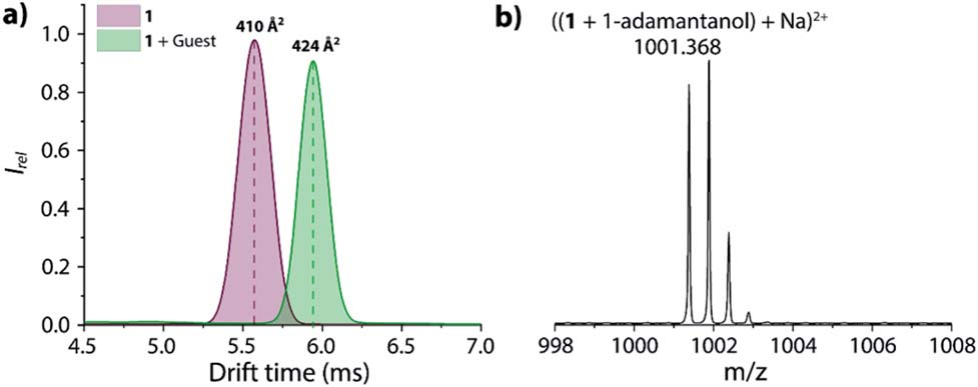
(a) The ion mobilogram of **1** in the absence and presence of 1-adamantanol shows an increase in collision cross-section (CCS) upon dye displacement in the inclusion complex. (b) ESI-MS of **1** in the presence of 1-adamantanol in positive ion mode.

The supramolecular behaviour of the CB7-linker-BC conjugates was characterized by advanced mass spectrometry experiments, ^1^H NMR, absorbance, and emission spectroscopy to evaluate if the CB7-linker-BC conjugates adopt the intended self-bound, “folded” conformation (shown in Fig. 1a), or if the linkers impose constraints. For simplicity, we focus the following description on chemosensor **1** and refer the reader to the ESI† for the corresponding results of chemosensor **2**. In order to get an insight into the inherent conformation of complex **1** in the absence of solvent effects, ion mobility spectrometry was performed. For chemosensor **1**, the parent peak appeared at *m*/*z* 925.3130 as dicationic species and can be assigned as (**1** + Na)^2+^ (see Fig. S24a† in the ESI). This species showed a drift time of 5.62 ms in the ion mobilogram, converting to a cross-section of 410 Å^2^ (Fig. 2a).

The uniform, symmetric curve shape indicated that only one defined conformation, *i.e.* the folded or unbound state occurs in the gas-phase, but not a mixture of both. Upon addition of the guest, 1-adamantanol, the characteristic complex was found at *m*/*z* 1001.368 and was assigned as ((**1** + 1-adamantanol) + Na)^2+^ (Fig. 2b). This species shows now a much larger drift time of 5.94 ms and a corresponding cross-section of 424 Å^2^ in the ion mobilogram. The relatively large increase in the cross-section upon guest binding can be understood by the displacement of the bound BC moiety and the unfolding of the conformation. For comparison, if chemosensor **1** would have occurred in its unfolded conformation in the gas phase, then guest inclusion into the cavity of CB7 would have not affected the BC moiety, and the drift time would have remained almost the same.^49^ For the analogous chemosensor **2** with the shorter TEG-linker, the ion mobilogram shows two peaks in drift time (4.87 and 5.17 ms) that likely both adopt folded conformations but may differ in the penetration depth of the BC moiety into the CB7 cavity (Fig. S24c†). Upon addition of 1-adamantanol, unfolding of chemosensor **2** occurs (see Fig. S24c and d† in the ESI) as is evidenced by the appearance of a single peak at a significantly higher drift time (5.66 ms).

**Fig. 3:**
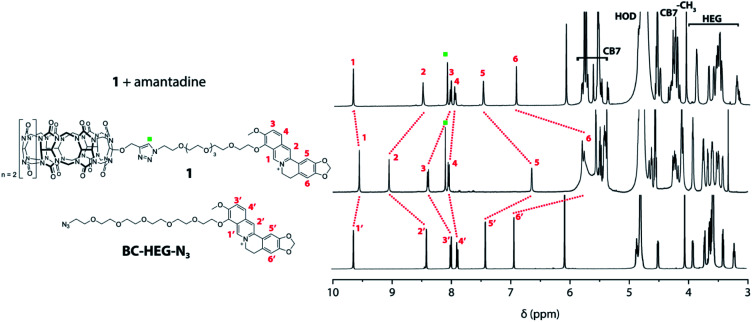
Overlay of ^1^H NMR (500 MHz, D_2_O) spectra of BC-HEG-N_3_ (bottom), chemosensor **1** (middle) and chemosensor **1** with one equivalent of amantadine (top).

Experiments in water were carried out to investigate if unimolecular, self-folding of the CB7–dye conjugates also occurs in solution. The ^1^H NMR spectrum of **1** in D_2_O shows three sets of peak areas that can be assigned to the aromatic peaks from the BC moiety (marked with red numbers) to the CB7 host, and the HEG linker (Fig. 3). The singlet at 8.10 ppm (marked with a green square) confirms that the click reaction has proceeded to form a triazole-moiety. Furthermore, by comparing with the ^1^H NMR spectrum of the corresponding BC-HEG-N_3_ compound before click reaction, it becomes clear that the aromatic protons of **1** exhibited both strong upfield and downfield shifts. In analogy to literature reports,^44^ this can be interpreted by the inclusion of the 1,3-benzodioxole moiety of BC into the CB7 cavity (shielding region of CB*n* hosts),^44,50^ while the isoquinolinium moiety resides in the deshielding carbonyl-fringed portal region of the host. Similar conclusions can be drawn for chemosensor **2**, see Fig. S18† for the analogous ^1^H NMR spectra analysis. Notably, the ^1^H NMR spectra of **2** shows broader peaks in the folded conformation than that of **1**, indicating that the distribution of conformers of **2** is present in solution. This finding agrees with conformer distribution seen in the ion mobilogram of folded **2**. After the addition of amantadine to the solution of **1** or **2**, the aromatic protons of the berberine moiety were shifted in a way that indicates displacement of the BC moiety from the host cavity (Fig. 3 and S18†). Note also the characteristic differences in the chemical shifts of the linker ethylene glycol proton signals between the folded and unfolded structure, and particularly the sharpening of the ^1^H NMR peaks of chemosensor **2** upon unfolding.

**Fig. 4:**
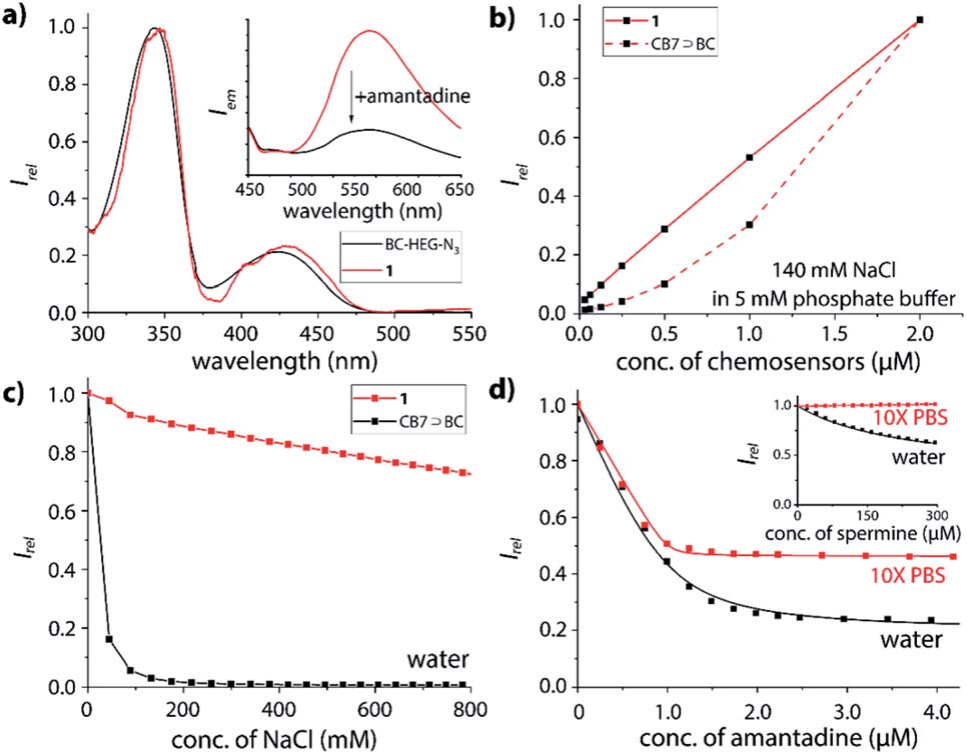
(a) UV-Vis absorption spectra of BC-HEG-N_3_ and **1**, each 5 μM in water. Inset: fluorescence emission spectra (*λ*_ex_ = 350 nm) of 1 μM **1** before and after addition of 1.5 eq. amantadine in water. (b) Plot of emission intensity at 540 nm in 140 mM NaCl and 5 mM phosphate buffer *versus* concentration of CB7⊃BC complex (dashed line) and **1** (solid line), *λ*_ex_ = 350 nm; (c) plot of emission intensity at 540 nm of 1 μM CB7⊃BC complex (black) and 1 μM **1** (red) in water *versus* concentration of NaCl, *λ*_ex_ = 350 nm. (d) Fitting plot of emission intensity at 540 nm of 1 μM **1** in water (black) and 10× PBS (red) upon addition of amantadine, *λ*_ex_ = 350 nm. Inset: chemosensor **1** upon addition of spermine in the same media. Note: the individual data points in (b and c) were connected by line segments to guide the eye and do not represent fitting curves. 10× PBS is consisting of 1.37 M NaCl, 27 mM KCl, 100 mM Na_2_HPO_4_ and 18 mM KH_2_PO_4_.

Furthermore, absorbance and emission spectroscopy were utilized because they allow for probing the supramolecular behaviour of the chemosensors at several orders of magnitude lower concentrations than NMR. The UV-Vis spectra of **1** in water shows an indicative 5 nm bathochromic shift at 350 nm in comparison to BC-HEG-N_3_ (Fig. 4a), which in analogy to literature reports^44^ suggests the inclusion of the BC chromophore into the CB7 cavity also at the micromolar concentration range. The emission spectra of **1** show a maximum at 540 nm by excitation at 350 nm (Fig. 4a, inset). The addition of amantadine leads to a strong decrease in emission intensity, indicative of displacement of the BC moiety from the cavity of **1**, because the BC fluorophore displays a much higher emission intensity inside the hydrophobic CB7 cavity than in water.^44^ Likewise, the emission intensity of the covalently bound CB7–dye conjugate **1** is a suitable indicator if the BC chromophore remains encapsulated by the host upon dilution. Indeed, we found a highly linear curve of the emission intensity *versus* the concentration of **1** even after several times of dilution down to 30 nM (Fig. 4b, solid line). In contrast, the intensity-concentration plot for the non-covalent bimolecular CB7⊃BC complex showed a convex curved shape because complex dissociation occurred at low concentration, see dashed line in Fig. 4b. (In both experiments, 140 mM NaCl was used to weaken the CB7-BC binding strength). Thus, also photophysical experiments confirm that chemosensor **1** adopts a unimolecular, folded structure, as is graphically depicted in Fig. 1a.

### Stability of CB7⊃dye complexes *vs.* chemosensor **1** in saline media

As the basis for subsequent analyte detection studies, the stability of our CB7-BC conjugates in saline media was investigated. According to previous reports, the stability of many CB*n*⊃dye complexes significantly decreases in the presence of salts, which reduces their utility for IDA sensing applications.^17,24,44,51–54^ Indeed, as shown in Fig. 4c (black line), the presence of around 100 mM sodium chloride leads to complete decomposition of the bimolecular CB7⊃BC assembly, which is in accordance with our expectation as competitive binding of metal cations to the carbonyl-fringed CB*n* portals occurs, *e.g. K*_a_ = 2.57 × 10^3^ M^−1^ for CB7 with Na^+^.^17^ Clearly, the bimolecular CB7⊃BC complex will therefore dissociate in biologically relevant media such as biofluids with typically high salt concentrations, *e.g.* 135–145 mM Na^+^ in plasma and 20–40 mM Na^+^ in urine for healthy humans.^55^ In contrast, the residual fluorescence intensity for chemosensor **1** remains at around 70% even at 800 mM NaCl (Fig. 4c, red line). Note that the modestly decreased emission intensity of **1** in saline media likely results from the formation of **1**⊃Na^+^, where the BC moiety remains encapsulated inside the CB7 cavity while the metal cation occupies the opposite CB7-portal.^18^ (ESI-MS experiments gave additional evidence that the binary complex of **1** with Na^+^ is prominently present also in the gas-phase, which was the species used for the ion-mobility studies.)

### Binding affinities of biorelevant analytes with the chemosensors

The host–guest binding properties of chemosensors **1** and **2** were investigated in desalinated *versus* saline media. As representative guests for CB7, several adamantyl derivatives (amantadine, 1-adamantanol),^25^ polyamines (cadaverine, spermine, spermidine)^56^ and steroids (nortestosterone)^24^ were selected because of their known high binding affinities for CB7.

Expectedly, both chemosensor **1** and **2** respond with emission quenching upon addition of competitively binding guests that displace the BC moiety from the cavity of the host; the change in emission intensity is slightly larger for **2** than for **1** (see ESI† for details), pointing towards a small but significant effect of the linker length on structures adopted in solution. Intriguingly, the binding curve of amantadine with **1** (Fig. 4d) or **2** (Fig. S35†) remains very steep even in 10× PBS, and binding may be even stronger than in deionized water (Fig. S33 and S35† in the ESI). Likewise, for the non-charged steroid nortestosterone, a very similar binding affinity with chemosensor **2** was found in 10× PBS and water (Table 1 and Fig. S47†).

**Table 1:** Summary of the binding constants determined by fluorescence titration experiments with chemosensor **1** and **2** at 25 °C. The estimated error in log *K*_a_ is 0.2

Guest	Medium	log *K*_a_		
		**1**	**2**	CB7
Amantadine	Water	7.4	7.9	12.6^c^
	1× PBS	>8^a^	>8^a^	—
	10× PBS	>8^a^	>8^a^	—
	Surine	>8^a^	>8^a^	—
Cadaverine	Water	3.7	4.0	8.4^d^
	1× PBS	3.5	3.7	—
	10× PBS	<1^b^	<1^b^	—
Spermine	Water	3.6	3.9	7.4^e^
	1× PBS	3.4	3.6	—
	10× PBS	<1^b^	<1^b^	—
Spermidine	Water	4.6	4.5	—
	10× PBS	<1^b^	<1^b^	—
1-Adamantanol	10× PBS	>8^a^	7.5	—
Nortestosterone	Water	4.5	4.5	7.1^d^
	1× PBS	4.3	4.4	6.6^f^
	10× PBS	4.1	4.8	—

a Lower estimate on the binding affinity, see Fig. S37 in the ESI.

b Binding affinity is too small to be determined by fluorescence titration.

c Determined by NMR.^58^

d Determined with berberine as indicator.^24,59^

e Determined with cyanostilbene as indicator.^57^

f Determined by isothermal titration calorimetry (ITC).^24^ 1× PBS is consisting of 137 mM NaCl, 2.7 mM KCl, 10 mM Na_2_HPO_4_ and 1.8 mM KH_2_PO_4_.

In contrast to the observation for amantadine or non-charged guest, the polycationic guests cadaverine, spermine and spermidine do not bind at all to our chemosensors in 10× PBS, while in deionized or minimal buffers, CB7 is a known high-affinity binder of polyamines such as spermidine (Table 1).^57^ Indeed, also chemosensor **1** is responsive to polyamines in deionized water (see Fig. S38–S43 in the ESI†). We found binding affinities in a range of 10^3^ to 10^5^ M^−1^ in the order spermidine > cadaverine ≥ spermine (Table 1).

Moreover, the binding titrations indicated that TEG-linker-based **2** forms a more strained folded conformation than HEG-linker-based **1**, as can be deduced from the observation that the binding affinities of chemosensor **2** are slightly but measurably higher than that of chemosensor **1** for the set of guests studied.

### Binding kinetics of biorelevant analytes with the chemosensors

**Fig. 5:**
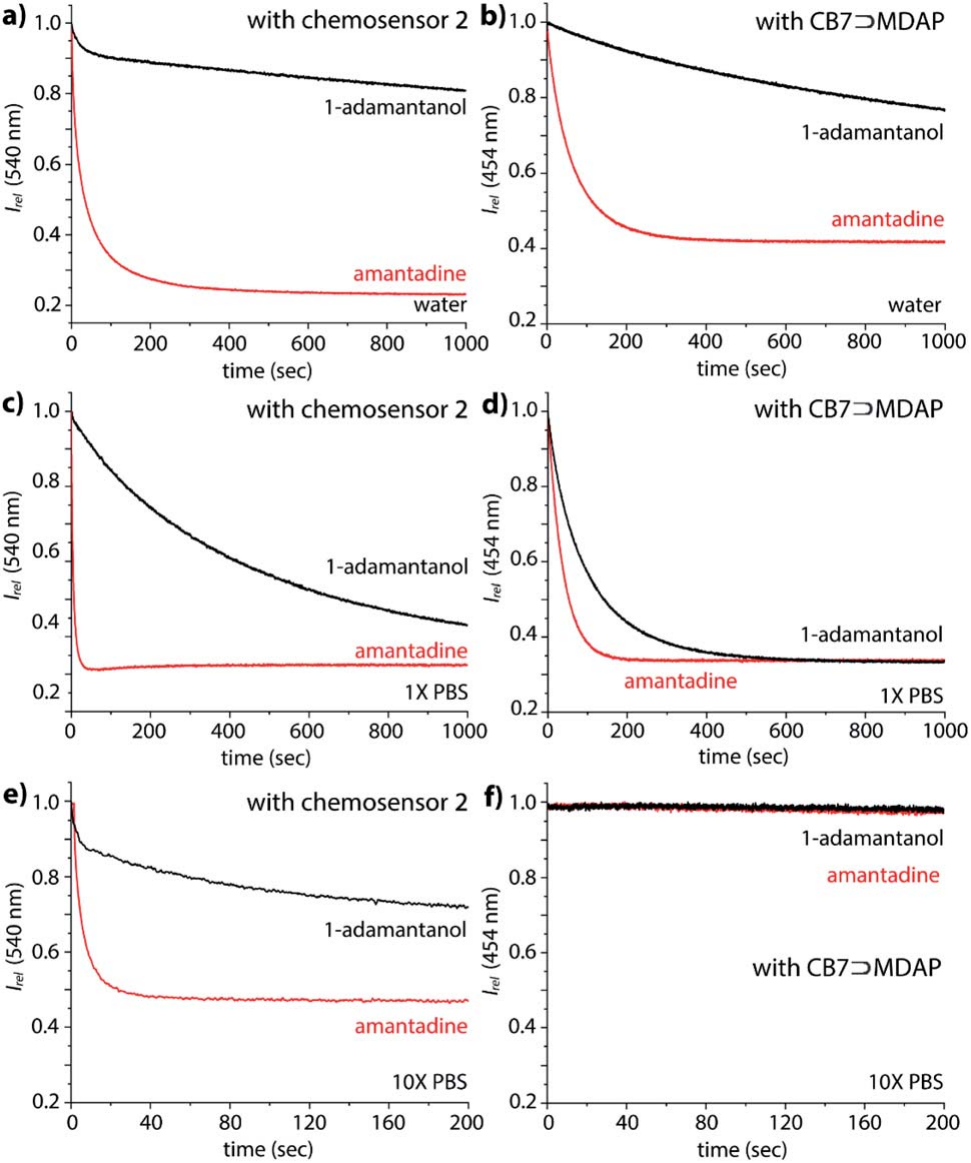
Fluorescence-based kinetic traces at 540 nm of 1.2 μM **2** and 2.09 μM amantadine (red) or 1-adamantanol (black) in (a) water, (c) 1× PBS and (e) 10× PBS at 25 °C, *λ*_ex_ = 350 nm; fluorescence-based kinetic traces at 454 nm of 1.8 μM MDAP, 1.2 μM CB7 and 2.09 μM amantadine (red) or 1-adamantanol in (b) water, (d) 1× PBS and (f) 10× PBS at 25 °C, *λ*_ex_ = 339 nm.

Interesting features of the chemosensors were also found for their binding kinetics: while amantadine, 1-adamantanol and the polycationic amines are all relatively strongly bound in water, the equilibration times follow the order polyamines (*n*+) > amantadine (1+) ≫ 1-adamantanol/nortestosterone, ranging from seconds (polyamines, amantadine) to several thousand of seconds for non-charged guests, which points to a charge-accelerating effect on the binding kinetics, see details in the ESI.† It has been observed previously that positively charged guests can form exclusion complexes prior to a flip-flop-mechanism into the cavity, which can speed up the binding kinetics.^60^ In fact, the binding kinetics of 1-adamantanol was so slow in desalinated water, as a result of which no binding isotherms for *K*_a_ value determination could be obtained in reasonable measurement times (Fig. 5a). In PBS, binding kinetics of amantadine binding is much faster than in water (Fig. 5), providing a practically convenient way to shorten assay times through addition of salts. Notably, amantadine and 1-adamantanol can still be readily distinguished based on their kinetic profile, allowing for selective sensing of amantadine in the presence of other non-charged high-affinity competitors (Fig. 5c and e).

The kinetics studies also indicate modestly faster reaction kinetics of **2** compared to **1**, which is in accordance with an energetically less-stable folded structure for the TEG-linked chemosensor variant **2** (Fig. S48†).

### Features and fundamental limitations of non-covalent CB7⊃dye reporter pairs

As an alternative to the use of a covalently dye–CB7 linked unimolecular chemosensor, we assessed if the bimolecular CB7⊃dye can be used for selective sensing of amantadine in saline media and biofluids (see next section) when suitable high affinity dyes are used. Through the known binding affinities for CB7 with metal cations, it is possible to estimate the apparent binding constant of bimolecular CB7⊃guest (and CB7⊃dye) complexes in the presence of salts *via*
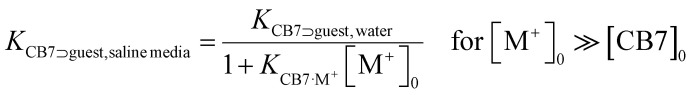


For instance, with *K*_CB7·Na+_ = 2.6 × 10^3^ M^−1^ for the competitive interaction of Na^+^ with CB7,^17^ the affinity of CB7 for amantadine still is expected as ∼10^12^ M^−1^ in the presence of 220 mM Na^+^. Following our previous analysis for the design of a CB8⊃dye IDA assay in biofluids, one would therefore conclude that indicator dyes within an affinity range of *K*_CB7⊃dye, saline media_ = 10^7^–10^11^ M^−1^ may be ideally suited for selectively detecting amantadine in the presence of salts and other weaker binding interferents, *e.g.* biogenic amines. However, this assumption turned out to be impractical:

(i) The lack of pH-unresponsive, high-affinity indicator dyes for CB7 sets the first “trivial” hindrance for establishing IDA-based sensing assays for amantadine in biofluids. MDAP is one of the indicator dyes for CB7 with the highest *K*_a_ value, 2.7 × 10^9^ M^−1^ in deionized water.^18,61^ Expectedly, its affinity to CB7 strongly drops with increasing salt content, reaching 1.8 × 10^6^ M^−1^, in 1× PBS (see Fig. S30† the ESI). In 10× PBS, its *K*_a_ value became so weak (*K*_a_ = 4.2 × 10^3^ M^−1^) that the CB7⊃MDAP reporter pair cannot be used in the micromolar concentration range (see Fig. S30† in the ESI). Consequently, the bimolecular CB7⊃MDAP complex is applicable for amantadine detection in saline media if they do not exceed the salt concentration of 1× PBS, however matrix effects will nevertheless be encountered if the salinity significantly differs between the biofluid samples.

**Fig. 6:**
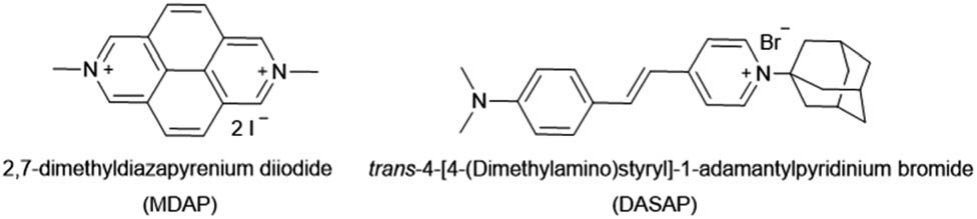
Chemical structures of MDAP and DASAP.

In deionized water, the CB7⊃MDAP reporter pair shows like the covalent chemosensors the ability to kinetically differentiate the positively charged drug amantadine from the non-charged analogue 1-adamantanol (Fig. 5b). However, the distinguishability of amantadine and 1-adamantanol by CB7⊃MDAP is much worsened upon increase of salinity to that of 1× PBS (Fig. 5d). In 10× PBS, the detection of amantadine through CB7⊃MDAP was not possible anymore due to disintegration of the non-covalent host–dye complex (Fig. 5f). Furthermore, the equilibration of amantadine with the CB7⊃MDAP complex is much slower than with the unimolecular chemosensors, causing longer assay times.

(ii) In order to overcome the affinity limitations of the CB7⊃MDAP host–dye complex that lead to its susceptibility to salts, we strove to develop a novel, pH-unresponsive, high-affinity indicator dye for CB7. Taking into consideration the strong binding affinities of adamantane for CB7,^11,14,15^ and the favorable photophysical properties of stilbene-type dyes,^17,41^ DASAP (see Fig. 6 for chemical structure), was considered to be a promising indicator dye, which could be obtained in 2 steps in 17.0% yield, see ESI.† DASAP showed promising properties as an indicator dye for CB7, *e.g.* a 10-fold increase in its emission intensity upon binding, a large Stokes shift (125 nm), a much more red-shifted absorption and emission spectra than MDAP, and a steep binding isotherm with CB7 both in deionized water and in 10× PBS (see Fig. S27† in the ESI). Its binding affinity to CB7 was estimated to exceed 10^7^ M^−1^ even in 10× PBS. At first sight, one could believe that the desired non-pH dependent, high-affinity dye for CB7 was found. However, its use proved to be completely impractical in an IDA format; no significant change in the emission intensity of the CB7⊃DASAP complex dissolved in water or 10× PBS was observed within 500 seconds of assay time even upon addition of 100× excess of amantadine, indicating that the CB7⊃DASAP complex is kinetically inert on the practically relevant time scale (see Fig. S28† in the ESI). Likewise, attempts to set up a guest-displacement-assay^59^ by addition of DASAP to the pre-equilibrated CB7⊃amantadine complex failed, again due to a slow unbinding kinetics for high affinity guests from CB7 (see Fig. S29† in the ESI).

### Chemosensor-based assay for the quantitative determination of amantadine in urine and saliva

Motivated by the ability of our chemosensor to detect amantadine even in ultra-high concentrated saline media (Fig. 4d and S32,† about 1.37 M NaCl) in comparison with the ineffectiveness of CB7⊃BC complexes in such environments, we transferred our binding studies first to synthetic urine (“surine”) as a well-defined media (see Fig. S50† in the ESI) and then to real human urine and saliva.

**Fig. 7:**
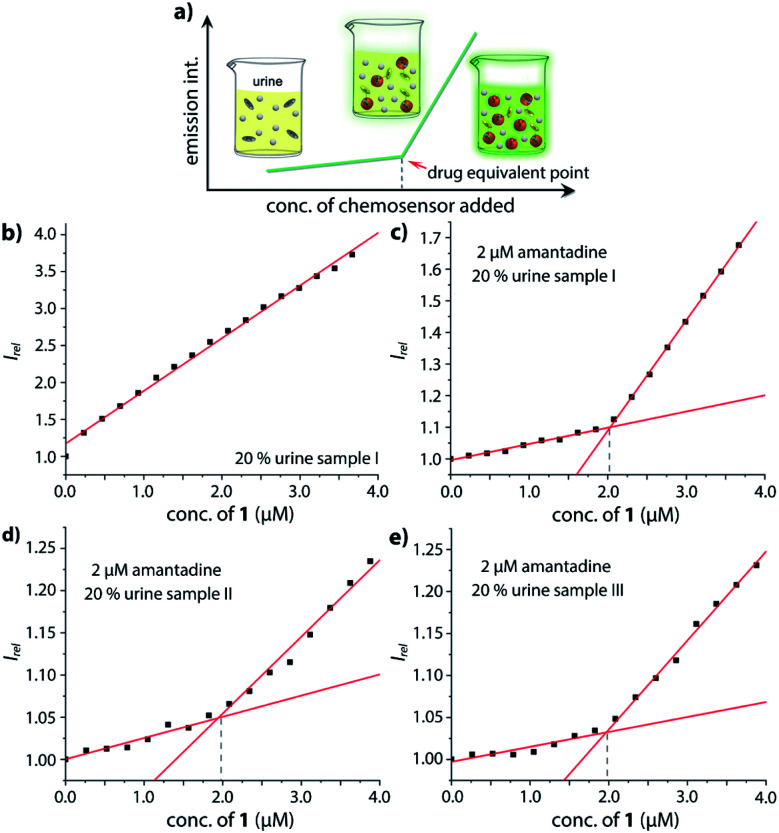
(a) Schematic representation of the analyte detection with CB7–dye conjugate chemosensors in real urine media and simulation of the trends in fluorescent intensity with increasing addition of conjugate chemosensor; (b) fitting plot of the normalized emission intensity at 540 nm *versus* concentration of **1** in 20% urine sample I; fitting plot of the normalized emission intensity at 540 nm *versus* concentration of **1** in 20% urine (c) sample I, (d) sample II and (e) sample III with a final concentration of 2 μM amantadine at 25 °C. This corresponds to an amantadine concentration estimate of ≈10 μM in the urine samples, which were indeed spiked with 10 μM amantadine.

Amantadine is typically prescribed in 100–200 mg daily doses and is typically excreted by 15–50% unchanged in urine.^62,63^ Thus, amantadine concentration of ≥40 μM will be found for up to 24 h in urine. Following established routines for spectroscopic urine diagnostics,^19,20,64^ urine samples were diluted with water in a 1 : 4 ratio in order to reduce the absorbance of real urine and to avoid inner filter effects. As the analyte concentration in urine samples is usually unknown, we devised a new method for using chemosensor **1** for quantifying amantadine in real urine samples. In short, chemosensors **1** and **2** were utilized as titrant to determine the amantadine concentration in several real urine samples (Fig. 7a). In the negative control sample (urine from a healthy donor that is not receiving amantadine treatment), the addition of chemosensor **1** to the assay medium follows a linear plot, in accordance with the proportional increase of the chemosensor concentration (Fig. 7b). Thus, unfolding of the chemosensor does not occur in the negative control medium, indicating that chemosensor **1** is not affected by the components present in this urine sample. Indeed, polyamines such as cadaverine and spermidine that are present in human urine at a low micromolar concentration^65^ did clearly not unfold the chemosensor, which is in accordance with their low binding affinity for the chemosensors in saline media.

In order to simulate urine from Parkinson's patients receiving amantadine treatment, urine samples from three healthy donors were each spiked with 10 μM amantadine and then diluted by water to reach a final concentration of 2 μM amantadine following the biological relevant concentration. As can be seen in Fig. 7c–e, the fluorescence intensity showed a significant two-stage distribution as a function of chemosensor concentration. The amantadine concentration is obtained by the intersect of the two straight lines described by two sets of data points: (1) those obtained with titrant concentrations under 1.0 equivalent and (2) those obtained with titrant concentrations above 1.0 equivalent. After fitting the two different stages, the intersection was found at approx. 2 μM amantadine in correspondence to the spiked amantadine concentration of 2 μM in 4 : 1 diluted urine medium (and thus 10 μM amantadine in urine). To probe the influence of sample-to-sample matrix variations, experiments were carried out in three different urine samples and gave in each instance the anticipated result (Fig. 7c–e).

It should be noted that amantadine detection in surine was also feasible through the CB7⊃MDAP reporter pair and the simulated presence of biogenic amines, only modestly disturbed the assay (Fig. S51 and S52†). However, the kinetics of amantadine binding to CB7⊃MDAP is slower than for the unimolecular chemosensors, and its susceptibility to interference from salts or other CB7-binding compounds is higher. Thus, it remains to be seen if the CB7⊃MDAP-based assay remains reliable under practical conditions, *i.e.* in real urine that contains varying concentrations of salts and organic components (*e.g.* other drugs).

**Fig. 8:**
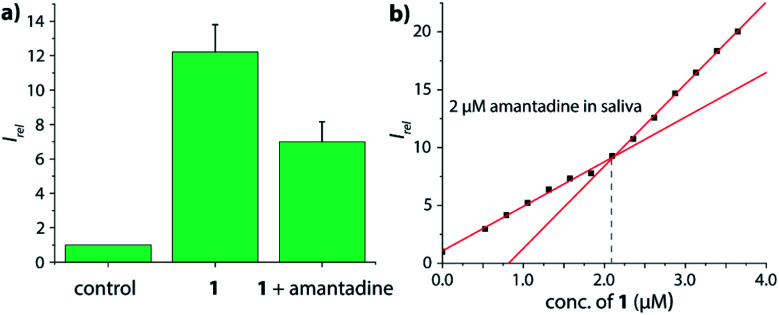
(a) Normalized emission intensity at 540 nm of 5 μM chemosensor **1** in human saliva prior and after addition of 5 μM amantadine. Error bars represent the standard deviation of the mean (*n* = 3). (b) Fitting plot of the normalized emission intensity at 540 nm obtained by the stepwise addition of **1** to saliva that was spiked with 2 μM amantadine, see the ESI† for repetition experiments and controls.

After oral administration, amantadine is also found in other body fluids, such as nasal, saliva and cerebrospinal fluid (CSF).^66,67^ For instance, the levels of amantadine in saliva equals approximately those in the serum in the range of 0.3 μg mL^−1^ to 0.8 μg mL^−1^ (around 2 μM to 5 μM).^68^ However, saliva contains much higher concentrations of biogenic amines (up to 400 μM cadaverine)^69^ than urine while being less saline, which would have caused undesirable interferences for indicator displacement assays with non-covalent CB7⊃dye reporter pairs. Fortunately, chemosensor **1** and **2** were found to be operational also in human saliva: as qualitative testing, saliva sample from a healthy donor was spiked with 5 μM chemosensor **1** and the emission was recorded by a microplate reader prior and after spiking with 5 μM amantadine (Fig. 8a), resulting in the expected emission intensity decrease upon unfolding of the chemosensor in the presence of the drug. Note that this assay can be performed directly in non-diluted saliva. Analogously to the assay type introduced for quantification of amantadine in urine, we also set to determine the amantadine concentration in non-diluted saliva; we used again 2 μM of drug spiking as a presentative showcase. Again, the expected amantadine concentration was returned from our assay in three different saliva samples by using chemosensor **1** and **2** (see Fig. 8b and S62–S69 in the ESI†). Investigation of the reporter pair CB7⊃MDAP for amantadine detection in artificial saliva showed a comparable performance to that of the chemosensors, but the simulated presence of cadaverine that can reach high micromolar concentrations in the saliva for some patients^69^ showed a stronger impact on CB7⊃MDAP than on **2** (see Fig. S70† in the ESI).

## Discussion

In this study, we designed and synthesized unimolecular chemosensors based on CB7–dye conjugates, which was inspired by the seminal work on a covalent β-cyclodextrins (β-CD)-methyl red chemosensors.^70^ The covalent linkage of an indicator dye to the host by a flexible tether is a promising approach towards dilution-stable chemosensors. For instance, dansyl-appended CD conjugates were synthesized and applied for detecting peroxide explosives in aqueous media.^71,72^ Recently, a coumarin labeled fluorogenic probe was studied for evaluation of phosphatidylserine on cell surfaces by using an intramolecular indicator displacement (IID) mechanism.^73^ Dilution-stable unimolecular CB*n*–dye conjugates are attractive chemosensors for diagnostics applications in biofluids because cucurbit[*n*]uril macrocycles (CB*n*, *n* = 5–8, 10, 12), especially CB7, show high binding affinities for several biologically relevant molecules in aqueous media such as amino acids and their derivatives, peptides, and drugs.^11,14–16,21,74–76^

Amantadine, which is a common drug to treat type A influenza viruses^77^ and dyskinesia associated with Parkinson's disease,^78–80^ is strongly bound to CB7 with affinities up to 10^12^ M^−1^.^58^ In the particular case of amantadine, the line between curing treatment and the occurrence of a series of side effects due to the accumulation of amantadine in the human body is narrow.^81,82^ Thus, the development of low-cost and widely applicable methods (*e.g.* for point-of-care use) that enables the routine quantification of the amantadine concentration in non-invasively available biofluids such as urine and saliva, may enable individual drug-dose adjustments and could contribute to improved life quality. To date, the most widely used detection methods for amantadine are chromatographic methods such as LC/GC/HPLC-MS,^83–85^ near-infrared spectroscopy,^86^ piezo-electric immunosensor,^87^ and electrochemical techniques, *e.g.*, potentiometry.^88,89^ Each of these methods has its strengths, however, all of them are largely constrained by complicating and long-lasting operating procedures and expensive equipment. Our emission-based supramolecular-analytical assay for amantadine in urine and saliva can be readily performed and does not require any sample pre-treatment steps. It is rapid, cost-efficient and parallelizable and could revolutionize amantadine sensing for drug dose adjustment in medical settings.

**Fig. 9:**
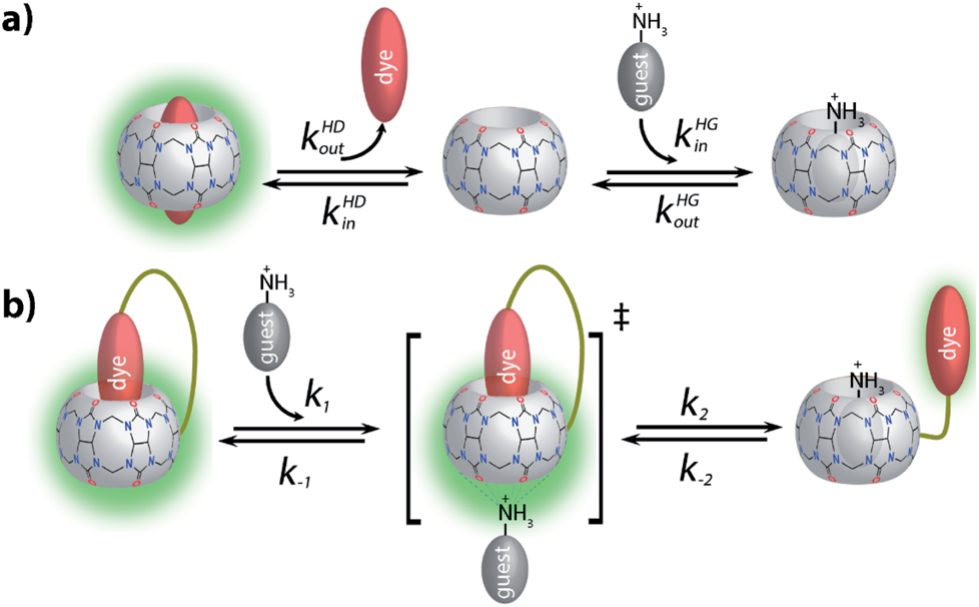
(a) Schematic representation of a S_N_1-type-mechanism for the guest-exchange process on analyte-induced conformational changes of non-covalent CB7⊃dye reporter pair complexes; (b) schematic representation of a S_N_2-type-mechanism for the guest-exchange process on analyte-induced conformational changes of CB7–dye conjugates.

In the following, the herein utilized unimolecular chemosensor design is compared to the much more frequently adopted use of non-covalent, bimolecular reporter pairs:

(1) The increase of the affinity of the dye for the host through covalent tethering, resulting in a higher degree of complexation also in the presence of disruptive competitors, enables now the use of intrinsically weakly binding indicator dyes that possess desirable photophysical properties. In fact, non-charged or monocationic CB7–indicator dyes such as berberine (BC), cannot be used in biofluids due to disintegration of their non-covalent reporter pair complex, *e.g. K*_a_ = 2.4 × 10^7^ M^−1^ for CB7⊃BC in water but <1.9 × 10^5^ M^−1^ in the presence of 50 mM NaCl.^44^ This restricted the choice of indicators to the few known high-affinity dyes for CB7, which are mostly dicationic aromatic species such as MDAP^18,61^ and 2,7-dimethyldiazaphenanthrenium (MDPT),^90^ that due to their electron-poor and symmetric structures possess rather unimpressive photophysical properties. For instance, this work demonstrated that the CB7⊃MDAP reporter pair is functional for indicator displacement assays in typical biorelevant saline buffer such as 1× PBS, where it retained a sufficiently high *K*_a_ value of 1.8 × 10^6^ M^−1^. However, a higher salinity was not tolerated by this reporter pair. From a photophysical point of view, MDAP is an applicable fluorophore (*λ*_em, max_ = 454 nm), nevertheless, dyes that absorb and emit in the visible region of the electromagnetic spectrum are practically often preferred. The herein advocated host–dye conjugation strategy widens the scope of functional chemosensors that can be prepared for use in (saline) aqueous media by lifting the previous constraints on searching for high-affinity dyes that simultaneously possess desirable photophysical properties.

(2) Inspired by the reported adamantly-BODIPY conjugate that functions as a p*K*_a_ shift indicator dye for CB7,^91^ we developed a novel pH-independent push–pull type high-affinity indicator dye (DASAP) for CB7 that functions both in water and in strongly saline buffers. At first, this design appeared to have overcome both the affinity and the photophysical limits encountered by MDAP, but practice uncovered a fundamental limitation of indicator displacement assay-based sensing for CB7⊃dye reporter pairs. Most indicator dyes reach a critical length such that upon inclusion complex formation with CB7, both portal regions of the hosts are occupied by the dye, as is pictorially depicted in Fig. 9a. This structural feature is also shared by the high-affinity CB7⊃dye complexes with dicationic dyes and has critical implications on the equilibration times for sensing assay times: in principle, the guest-exchange process for host–guest complexes can occur through a S_N_1-type or S_N_2-type-mechanism. For the example case of non-covalent CB7⊃dye complexes, the S_N_1 mechanism appears to be the main pathway,^92^ proceeding through the exit of the dye from the host as first and rate limiting step (Fig. 9a). Conversely, a S_N_2-type dye–guest exchange mechanism would require the formation of a guest·CB7·dye transient complex, whose formation is impeded by the protruding indicator dye. (BC may be one exception as the dye does not penetrate through the whole cavity, allowing simultaneous binding of the dye and a cationic species, *e.g.* metal ions.^18^ However, the CB7⊃BC complex is salt labile and cannot be used in biofluids, see the Results section).

Being limited to an S_N_1-type dye–guest exchange mechanism for most CB7⊃dye reporter pair complexes, it becomes clear that the higher the affinity of the dye for the host, the lower will be the rate constant for the exit of the dye from the host because *k*_out_ = *k*_in_*K*_a_^−1^, and *k*_in_ is relatively similar amongst different guests for the same CB*n* homologue.^18,92–94^ These counteracting effects result in a fundamental limitation for applying CB*n*-based IDA for sensing applications in biofluids with non-covalent reporter pairs. On the one hand, the affinity of the dye needs to be high enough to avoid disintegration of the CB*n*⊃dye reporter pair in the presence of salts and other competitively binding guests. While, on the other hand, high-affinity dyes cause overall slowed down exchange kinetics, which result in prolonged assay times. Note also that similar rational holds for guest-displacement assays (GDA),^59^ which likewise show impractically slow equilibration times for the detection of high affinity guests such as amantadine, *e.g.* see the Results section.

Indeed, while the thermodynamic and kinetic parameters of the CB7⊃MDAP complex are fortunately counterbalanced (*K*_a_ = 2.7 × 10^9^ M^−1^, *k*_in_ = 2.4 × 10^7^ M^−1^ s^−1^, *k*_out_ = 9.0 × 10^−3^ s^−1^ in water, see ref. 92), thereby enabling its practical use for sensing applications in saline media and biofluids, they also mark a host–dye affinity limit beyond which assay performance will drop because of a too slow exit of the dye from the host. For instance, the high-affinity CB7⊃DASAP complex was found to be impracticable as a reporter pair for the detection of amantadine despite its excellent salt stability and favorable photophysical properties. Similarly, our previously reported CB8⊃MPCP reporter pair (*K*_a_ = 3.9 × 10^12^ M^−1^, *k*_in_ = 2.0 × 10^7^ M^−1^ s^−1^, *k*_out_ = 5.1 × 10^−6^ s^−1^ in water, see ref. 21 and 92) also reached the limiting affinity region for non-covalent CB8⊃dye reporter pairs, resulting in assay times of several minutes even under the accelerating effect of salts present in biofluids. It is worth noting that CB8 has a lower inherent affinity to biorelevant metal cations such as Na^+^, K^+^ and Ca^2+^ than CB7,^17^ and thus CB8⊃dye reporter pairs are less adversely affected by salts, permitting the use of a lower affinity range for the reporter dye than for the analogous CB7⊃dye reporter pairs in saline media. Conversely, CB6-based chemosensing assays will likely not be operational in biofluids if non-covalent CB6⊃dye complexes are used as the reporter pairs, because the ingression rates are comparably low for CB6 complexes due to the constricted portal region^52,92^ (thus limiting the acceptable affinity values for the CB6⊃dye reporter pairs), while on the other hand CB6 has a sizeable affinity for Na^+^, K^+^ and Ca^2+^.^17^ The covalent chemosensor design overcomes these issues, and thus will be particularly attractive also for CB6-based chemosensors.

For chemosensors **1** and **2**, the covalently-tethered indicator dye BC does not protrude to both CB7 portals, but conversely one CB7 portal region remains accessible, as was confirmed by ^1^H NMR experiments and molecular modelling. The situation is graphically depicted in Fig. 9b and is consequence from the choice of berberine as the indicator dye (that inherently does not fully penetrate the CB7 cavity, see above) and from the restricted linker lengths. Importantly, these covalently-linked chemosensors possessing one accessible CB7-portal region can form transient complexes with positively-charged guests through cation–carbonyl interactions. Thus, the S_N_2 dye–guest exchange pathway can be adopted by positively charged guests such as amantadine, rationalizing much faster equilibration of chemosensors **1** and **2** than of CB7⊃MDAP with this cationic target analyte, and also explaining the large kinetic differences observed between amantadine and 1-amantanol binding. The covalent chemosensor design strategy thereby opens new opportunities for improving the distinguishability of different analytes through their characteristic binding kinetics. In this study, different linker lengths of chemosensors **1** and **2** were shown to give distinctly different structural, thermodynamic, spectroscopic and kinetic behaviour in the gas phase and in solution, suggesting that future exploration of the linker length can be an additional chemosensor design-criterium, for instance when indicator dyes other than berberine are used that could penetrate fully into the host cavity.

Our study also revealed new supramolecular effects for CB*n*-based host–guest complexes, *e.g.* the “expected” strongly attenuated binding affinity of the chemosensors for polyamines in saline buffer compared to the strengthening of the binding for non-charged and mono-charged guests upon salinity increase of the medium. Thus, practical assay performance – *e.g.* in urine – can be improved by increase in the salinity of the medium which at the same time will shorten the equilibration times. Note that a similar strategy cannot be applied to the CB7⊃MDAP (or CB7⊃BC) reporter pair-based assays because binding strength of the bimolecular host–dye complex becomes too weak in high salinity media.

Lastly, exploration of the characteristic effects on the thermodynamics and kinetics of analyte binding through addition of salts to the assay medium will be useful for pattern recognition-based differentiation of organic analytes.

## Conclusions

In this study, we designed and synthesized two novel CB7-based unimolecular chemosensors, verified their conformation as a back-folded unimolecular structure in the gas-phase and in solution, and reported on their interaction with biologically relevant molecules. Both conjugates displayed excellent resistance to dilution and salt effects and remained functional chemosensors even in ∼10^6^ times excess of sodium chloride. In biologically relevant buffered saline and even in real urine, the chemosensors displayed a great selectivity for Parkinson's drug amantadine over potential interferents such as polyamines (too low affinity binding in saline media) or hydrophobic, non-charged guests such as the steroid nortestosterone (very slow binding). To the best of our knowledge, this is the first CB*n*-based unimolecular chemosensor that can be utilized for the selective detection of a blockbuster drug, *i.e.* amantadine, in the medically relevant concentration range in human urine and saliva.^95^ While the non-covalent CB7⊃MDAP reporter pair also may be a viable choice for amantadine detection in biofluids, this study nevertheless uncovered important and general shortcomings of the use of high-affinity host–guest reporter pairs, that will be even more limiting for CB6-based reporter pairs. We believe that the covalent chemosensor design strategy can overcome many of these limitations and can increase the analyte-detection selectivity of the assays by providing access to a S_N_2-type guest exchange mechanism.

The “chemosensor-standard-addition-method” that was utilized here for amantadine quantification is particularly useful because it circumvents the otherwise often observed matrix-to-matrix effects between different biofluid specimens, *e.g.* due to different colors, emission backgrounds or salinity of urine samples. On account of the simplicity, cost-effectiveness and speed of the supramolecular analytical method developed, it may find future use in established diagnostic laboratories or point-of-care applications.

## Conflicts of interest

There are no conflicts to declare.

## Supplementary Material

SC-011-D0SC03079A-s001

## References

[cit1] You L., Zha D., Anslyn E. V. (2015). Chem. Rev..

[cit2] Schneider H.-J., Yatsimirsky A. K. (2008). Chem. Soc. Rev..

[cit3] Geng W.-C., Sessler J. L., Guo D.-S. (2020). Chem. Soc. Rev..

[cit4] Dsouza R. N., Hennig A., Nau W. M. (2012). Chem. – Eur. J..

[cit5] Bai L.-M., Zhou H., Liu W.-E., Chai H., Yang L.-P., Yan W., Zhang W., Yang H.-H., Jiang W. (2019). Chem. Commun..

[cit6] Nguyen B. T., Anslyn E. V. (2006). Coord. Chem. Rev..

[cit7] Jo H. H., Lin C.-Y., Anslyn E. V. (2014). Acc. Chem. Res..

[cit8] Beatty M. A., Borges-González J., Sinclair N. J., Pye A. T., Hof F. (2018). J. Am. Chem. Soc..

[cit9] Liu Y., Perez L., Mettry M., Easley C. J., Hooley R. J., Zhong W. (2016). J. Am. Chem. Soc..

[cit10] Zheng Z., Geng W.-C., Gao J., Wang Y.-Y., Sun H., Guo D.-S. (2018). Chem. Sci..

[cit11] Sinn S., Biedermann F. (2018). Isr. J. Chem..

[cit12] Lagona J., Mukhopadhyay P., Chakrabarti S., Isaacs L. (2005). Angew. Chem., Int. Ed..

[cit13] Mutihac R.-C., Bunaciu A. A., Buschmann H.-J., Mutihac L. (2020). J. Inclusion Phenom. Macrocyclic Chem..

[cit14] Assaf K. I., Nau W. M. (2015). Chem. Soc. Rev..

[cit15] Barrow S. J., Kasera S., Rowland M. J., del Barrio J., Scherman O. A. (2015). Chem. Rev..

[cit16] Shetty D., Khedkar J. K., Park K. M., Kim K. (2015). Chem. Soc. Rev..

[cit17] Zhang S., Grimm L., Miskolczy Z., Biczók L., Biedermann F., Nau W. M. (2019). Chem. Commun..

[cit18] Miskolczy Z., Megyesi M., Biczók L., Prabodh A., Biedermann F. (2020). Chem. – Eur. J..

[cit19] Zhu L., Zhao Z., Zhang X., Zhang H., Liang F., Liu S. (2018). Molecules.

[cit20] Yang H., Liu Y., Yang L., Liu K., Wang Z., Zhang X. (2013). Chem. Commun..

[cit21] Sinn S., Spuling E., Bräse S., Biedermann F. (2019). Chem. Sci..

[cit22] Prabodh A., Bauer D., Kubik S., Rebmann P., Klärner F. G., Schrader T., Delarue Bizzini L., Mayor M., Biedermann F. (2020). Chem. Commun..

[cit23] Minami T., Esipenko N. A., Akdeniz A., Zhang B., Isaacs L., Anzenbacher P. (2013). J. Am. Chem. Soc..

[cit24] Lazar A. I., Biedermann F., Mustafina K. R., Assaf K. I., Hennig A., Nau W. M. (2016). J. Am. Chem. Soc..

[cit25] Moghaddam S., Yang C., Rekharsky M., Ko Y. H., Kim K., Inoue Y., Gilson M. K. (2011). J. Am. Chem. Soc..

[cit26] Biedermann F., Uzunova V. D., Scherman O. A., Nau W. M., De Simone A. (2012). J. Am. Chem. Soc..

[cit27] Schneider H.-J. (2009). Angew. Chem., Int. Ed..

[cit28] Houk K. N., Leach A. G., Kim S. P., Zhang X. (2003). Angew. Chem., Int. Ed..

[cit29] Ayhan M. M., Karoui H., Hardy M., Rockenbauer A., Charles L., Rosas R., Udachin K., Tordo P., Bardelang D., Ouari O. (2015). J. Am. Chem. Soc..

[cit30] Ayhan M. M., Karoui H., Hardy M., Rockenbauer A., Charles L., Rosas R., Udachin K., Tordo P., Bardelang D., Ouari O. (2016). J. Am. Chem. Soc..

[cit31] Park K. M., Baek K., Ko Y. H., Shrinidhi A., Murray J., Jang W. H., Kim K. H., Lee J.-S., Yoo J., Kim S., Kim K. (2018). Angew. Chem., Int. Ed..

[cit32] An J., Kim S., Shrinidhi A., Kim J., Banna H., Sung G., Park K. M., Kim K. (2020). Nat. Biomed. Eng..

[cit33] Vinciguerra B., Cao L., Cannon J. R., Zavalij P. Y., Fenselau C., Isaacs L. (2012). J. Am. Chem. Soc..

[cit34] Dong N., He J., Li T., Peralta A., Avei M. R., Ma M., Kaifer A. E. (2018). J. Org. Chem..

[cit35] Bockus A. T., Smith L. C., Grice A. G., Ali O. A., Young C. C., Mobley W., Leek A., Roberts J. L., Vinciguerra B., Isaacs L., Urbach A. R. (2016). J. Am. Chem. Soc..

[cit36] Zhang S., Assaf K. I., Huang C., Hennig A., Nau W. M. (2019). Chem. Commun..

[cit37] Gong B., Choi B. K., Kim J. Y., Shetty D., Ko Y. H., Selvapalam N., Lee N. K., Kim K. (2015). J. Am. Chem. Soc..

[cit38] Kim K. L., Sung G., Sim J., Murray J., Li M., Lee A., Shrinidhi A., Park K. M., Kim K. (2018). Nat. Commun..

[cit39] Li M., Lee A., Kim K. L., Murray J., Shrinidhi A., Sung G., Park K. M., Kim K. (2018). Angew. Chem., Int. Ed. Engl..

[cit40] Li M., Lee A., Kim S., Shrinidhi A., Park K. M., Kim K. (2019). Org. Biomol. Chem..

[cit41] Dsouza R. N., Pischel U., Nau W. M. (2011). Chem. Rev..

[cit42] Shaikh M., Mohanty J., Singh P. K., Nau W. M., Pal H. (2008). Photochem. Photobiol. Sci..

[cit43] LaManna J. C., McCracken K. A. (1984). Anal. Biochem..

[cit44] Megyesi M., Biczók L., Jablonkai I. (2008). J. Phys. Chem. C.

[cit45] Miskolczy Z., Biczók L. (2014). J. Phys. Chem. B.

[cit46] Cao H., Liao S., Zhong W., Xiao X., Zhu J., Li W., Wu X., Feng Y. (2017). Molecules.

[cit47] Lu G., Lam S., Burgess K. (2006). Chem. Commun..

[cit48] Zhang S., Domínguez Z., Assaf K. I., Nilam M., Thiele T., Pischel U., Schedler U., Nau W. M., Hennig A. (2018). Chem. Sci..

[cit49] Lee T.-C., Kalenius E., Lazar A. I., Assaf K. I., Kuhnert N., Grün C. H., Jänis J., Scherman O. A., Nau W. M. (2013). Nat. Chem..

[cit50] Zhao N., Liu L., Biedermann F., Scherman O. A. (2010). Chem.–Asian J..

[cit51] Biedermann F., Elmalem E., Ghosh I., Nau W. M., Scherman O. A. (2012). Angew. Chem., Int. Ed..

[cit52] Márquez C., Hudgins R. R., Nau W. M. (2004). J. Am. Chem. Soc..

[cit53] Ong W., Kaifer A. E. (2004). J. Org. Chem..

[cit54] Jeon Y.-M., Kim J., Whang D., Kim K. (1996). J. Am. Chem. Soc..

[cit55] Maciel A. T., Vitorio D., Salles L. D., Park M. (2014). Anaesthesia and Intensive Care.

[cit56] Park K. M., Kim J., Ko Y. H., Ahn Y., Murray J., Li M., Shrinidhi A., Kim K. (2018). Bull. Chem. Soc. Jpn..

[cit57] Lee E.-C., Kim H.-J., Park S. Y. (2019). Chem.–Asian J..

[cit58] Liu S., Ruspic C., Mukhopadhyay P., Chakrabarti S., Zavalij P. Y., Isaacs L. (2005). J. Am. Chem. Soc..

[cit59] Sinn S., Krämer J., Biedermann F. (2020). Chem. Commun..

[cit60] Marquez C., Nau W. M. (2001). Angew. Chem., Int. Ed..

[cit61] Sindelar V., Cejas M. A., Raymo F. M., Kaifer A. E. (2005). New J. Chem..

[cit62] Drugs.com, Amantadine Dosage, https://www.drugs.com/dosage/amantadine.html

[cit63] Bai PharmD D., What You Need to Know About Amantadine for Parkinson Disease, https://www.neurologylive.com/clinical-focus/what-you-need-to-know-about-amantadine-for-parkinson-disease

[cit64] Wang G.-Q., Qin Y.-F., Du L.-M., Li J.-F., Jing X., Chang Y.-X., Wu H. (2012). Spectrochim. Acta, Part A.

[cit65] Lipton A., Sheehan L. M., Kessler Jr G. F. (1975). Cancer.

[cit66] Kornhuber J., Quack G., Danysz W., Jellinger K., Danielczyk W., Gsell W., Riederer P. (1995). Neuropharmacology.

[cit67] Aoki F. Y., Sitar D. S. (1988). Clin. Pharmacokinet..

[cit68] Bennett J. E., Dolin R., Blaser M. J. and Mandell G. L., Mandell, Douglas, and Bennett's Principles and Practice of Infectious Diseases, Elsevier Health Sciences, 2009

[cit69] Goldberg S., Kozlovsky A., Gordon D., Gelernter I., Sintov A., Rosenberg M. (1994). J. Dent. Res..

[cit70] Ueno A., Kuwabara T., Nakamura A., Toda F. (1992). Nature.

[cit71] Ikeda H., Nakamura M., Ise N., Oguma N., Nakamura A., Ikeda T., Toda F., Ueno A. (1996). J. Am. Chem. Soc..

[cit72] Almenar E., Costero A. M., Gaviña P., Gil S., Parra M. (2018). R. Soc. Open Sci..

[cit73] Zwicker V. E., Oliveira B. L., Yeo J. H., Fraser S. T., Bernardes G. J. L., New E. J., Jolliffe K. A. (2019). Angew. Chem., Int. Ed..

[cit74] Macartney D. H. (2011). Isr. J. Chem..

[cit75] Urbach A. R., Ramalingam V. (2011). Isr. J. Chem..

[cit76] Kasera S., Herrmann L. O., Barrio J. d., Baumberg J. J., Scherman O. A. (2014). Sci. Rep..

[cit77] Prud'homme I. T., Zoueva O., Weber J. M. (1997). J. Clin. Virol..

[cit78] Vollum D. I., Parkes J. D., Doyle D. (1971). BMJ [Br. Med. J.].

[cit79] Cook P. E., Dermer S. W., Mcgurk T. (1986). Can. J. Psychiatry.

[cit80] Kornhuber J., Bormann J., Hübers M., Rusche K., Riederer P. (1991). Eur. J. Pharmacol., Mol. Pharmacol. Sect..

[cit81] Hayden F. G. (2006). Antiviral Res..

[cit82] He G., Qiao J., Dong C., He C., Zhao L., Tian Y. (2008). Antiviral Res..

[cit83] Bhadoriya A., Rathnam S., Dasandi B., Parmar D., Sanyal M., Shrivastav P. S. (2018). J. Pharm. Anal..

[cit84] Sioufi A., Pommier F. (1980). J. Chromatogr. B: Biomed. Sci. Appl..

[cit85] Duh T.-H., Wu H.-L., Pan C.-W., Kou H.-S. (2005). J. Chromatogr. A.

[cit86] Dou Y., Sun Y., Ren Y., Ju P., Ren Y. (2005). J. Pharm. Biomed. Anal..

[cit87] Yun Y., Pan M., Wang L., Li S., Wang Y., Gu Y., Yang J., Wang S. (2019). Anal. Bioanal. Chem..

[cit88] Abdel-Ghani N. T., Shoukry A. F., Hussein S. H. (2002). J. Pharm. Biomed. Anal..

[cit89] Jalali F., Maghooli R. (2009). Anal. Sci..

[cit90] Ling Y., Wang W., Kaifer A. E. (2007). Chem. Commun..

[cit91] Alnajjar M. A., Bartelmeß J., Hein R., Ashokkumar P., Nilam M., Nau W. M., Rurack K., Hennig A. (2018). Beilstein J. Org. Chem..

[cit92] Prabodh A., Sinn S., Grimm L., Miskolczy Z., Megyesi M., Biczok L., Bräse S., Biedermann F. (2020). Chem. Commun..

[cit93] Masson E., Raeisi M., Kotturi K. (2018). Isr. J. Chem..

[cit94] Appel E. A., Biedermann F., Hoogland D., del Barrio J., Driscoll M. D., Hay S., Wales D. J., Scherman O. A. (2017). J. Am. Chem. Soc..

[cit95] Parkinson's Disease Drugs Market – Growth, Trends, and Forecasts, 2020–2025, https://www.mordorintelligence.com/industry-reports/parkinsons-disease-drugs-market

