# Supplementary material for: Covalent cucurbit[7]uril–dye conjugates for sensing in aqueous saline media and biofluids
Source: Chem Sci. 2020 Sep 22;11(41):11142–53. doi: 10.1039/d0sc03079a (PMC8162441; doi:10.1039/d0sc03079a)
Supplement: SC-011-D0SC03079A-s001 [file SC-011-D0SC03079A-s001.pdf]

Electronic Supplementary Material (ESI) for Chemical Science.
This journal is © The Royal Society of Chemistry 2020

Electronic Supplementary Information

Covalent Cucurbit[7]uril-Dye Conjugates for Sensing in Aqueous Saline Media and Biofluids

Changming Hu,[a] Laura Grimm,[a] Amrutha Prabodh,[a] Ananya Baksi,[a],[b] Alicja Siennicka,[a] Pavel A. Levkin,[c] Manfred M. Kappes,[a],[b] and Frank Biedermann*[a]

[a] *Institute of Nanotechnology (INT), Karlsruhe Institute of Technology (KIT), Hermann-von-Helmholtz Platz 1, 76344 Eggenstein-Leopoldshafen, Germany*

[b] *Institute of Physical Chemistry (IPC), Karlsruhe Institute of Technology (KIT), Fritz-Haber-Weg 6, 76131 Karlsruhe, Germany*

[c] *Institute of Biological and Chemical Systems – Functional Molecular Systems (IBCS-FMS), Karlsruhe Institute of Technology (KIT), Hermann-von-Helmholtz Platz 1, 76344 Eggenstein-Leopoldshafen, Germany*

Table of Contents

Experimental Details 3

Synthesis and Characterization 6

Synthesis of berberrubine (BC-OH) 6

Synthesis of Dibromo-hexaethylene glycol (Br-HEG-Br) 7

Synthesis of berberine-hexaethylene glycol-bromide (BC-HEG-Br) 8

Synthesis of berberine-hexaethylene glycol-azide (BC-HEG-N_3) 9

Synthesis of cucurbit[7]uril-HEG-berberine conjugate (1) 11

Synthesis of Dibromo-tetraethylene glycol (Br-TEG-Br) 12

Synthesis of berberine-tetraethylene glycol-bromide (BC-TEG-Br) 13

Synthesis of berberine-tetraethylene glycol-azide (BC-TEG-N_3) 14

Synthesis of cucurbit[7]uril-TEG-berberine conjugate (2) 16

Synthesis of *N*-adamantyl-4-methylpyridine (Ad-MePy) 18

Synthesis of *trans*-4-[4-(Dimethylamino)styryl]-1-adamantylpyridinium bromide (DASAP) 19

Ion mobilogram and ESI-MS spectra of 1 and 2 21

Fluorescence spectrum analysis 22

Chemical structures of analytes and reference dyes 22

Titration of cucurbit[7]uril to berberine and its derivatives 22

Titration of cucurbit[7]uril to DASAP 23

Fluorescence kinetic traces of amantadine addition to CB7⊃DASAP 24

Fluorescence kinetic traces of DASAP to CB7⊃amantadine 24

Fluorescence titration and kinetic traces of cucurbit[7]uril to MDAP 25

Absorption and emission spectra of chemosensor 2 26

Fluorescence titration of NaCl to 1 and 2 26

Fluorescence titration of amantadine to 1 and 2 27

Fluorescence titration of cadaverine to 1 and 2 30

Fluorescence titration of spermine to 1 and 2.. 32

Fluorescence titration of spermidine to 1 and 2... 34

Fluorescence titration of 1-adamantanol to 1 and 2... 36

Fluorescence titration of nortestosterone to 1 and 2 37

Overlay of kinetic traces on addition of amantadine to 1 and 2.. 39

Detection limit of 2 for amantadine 39

Amantadine determination in biofluid probes 40

Amantadine determination with 1 and 2 in surine... 40

Amantadine determination with CB7⊃MDAP in surine 41

Amantadine determination with 2 and CB7⊃MDAP in surine in the presence of spermine... 41

100 nM amantadine detection by 2 in surine.. 42

Amantadine determination with 1 and 2 in urine 43

Urine sample I.. 43

Urine sample II... 46

Urine sample III 47

Amantadine determination with 1 and 2 in saliva 48

Saliva sample I 48

Saliva sample II.. 50

Saliva sample III... 51

Amantadine determination with 2 and CB7⊃MDAP in artificial saliva in the absence or presence of cadaverine 52

References 53

Experimental Details

Material. All solvents were used as received from suppliers without any further purification. All purchased chemicals were used as received. Cucurbit[7]uril (CB7)[1], mono-hydroxylated cucurbit[7]uril (CB7-OH)[2], mono-propargyloxylated cucurbit[7]uril (CB7-Opr)[3] and 2,7-dimethyldiazapyrenium diiodide (MDAP)[4] were synthesized according to established literature methods. The stock solutions of 1X (137 mM NaCl, 2.7 mM KCl, 10 mM Na_2HPO_4 and 1.8 mM KH_2PO_4) and 10X (1.37 M NaCl, 27 mM KCl, 100 mM Na_2HPO_4 and 18 mM KH_2PO_4) phosphate-buffered saline (PBS) were prepared from GibcoTM PBS tablets by dissolving a tablet in 500 mL or 50 mL of distilled water, respectively. The pH was 7.45 and required no adjustment. The artificial saliva was from Pickering Laboratories and used as received. Surine was from Cerilliant and used as received. MDAP, CB7, chemosensor **1** and chemosensor **2** stock solutions were prepared in Milli-Q water and then diluted in 1X or 10X PBS for the measurements. The concentration of MDAP, berberine hydrochloride (BC) and nortestosterone stock solutions were determined accurately by using their molar absorption coefficients (MDAP: 7800 $M^{-1}cm^{-1}$ at 393 nm, BC: 22300 $M^{-1}cm^{-1}$ at 344 nm and nortestosterone: 15320 $M^{-1}cm^{-1}$ at 248 nm) by UV-Vis absorption titration measurements in Milli-Q water. The concentration of CB7 and amantadine hydrochloride stock solution were determined by fluorescence titration against a known concentration of MDAP dye and CB7⊃MDAP receptor complex, respectively, by exciting the sample at 339 nm and collecting the emission intensity at 454 nm in Milli-Q water. The 1-adamantanol stock solution concentration was determined by ITC measurements. The stock solution concentrations of chemosensor **1** and **2** were determined by fluorescence titration against a known concentration of amantadine hydrochloride by exciting the sample at 350 nm and collecting the emission intensity at 540 nm in Milli-Q water. The stock solution of polyamine (cadaverine, spermine and spermidine) were prepared to the aqueous solution with certain concentration directly by weighing. In general, chemosensor solutions should be stirred for 30 minutes in the desired medium to ensure full equilibration along with a stable baseline for spectroscopical measurements.

Nuclear Magnetic Resonance (NMR) Spectroscopy. ^{1}H and ^{13}C NMR spectra were recorded either in deuterium oxide, Chloroform-d_3 or DMSO-d_6 on a Bruker Avance 500 spectrometer at 25°C. The

^1H and ^{13}C NMR chemical shifts (δ) are given in ppm and refer to residual protons on the corresponding deuterated solvent.

High-Performance Liquid Chromatography (HPLC). Analytical HPLC experiments were performed on an LC-2000Plus HPLC system equipped with a UV-2075 UV-Vis detector and a Kromasil 100 C18 5 μM LC column (250 × 4.6 mm, Agela) at a flow rate of 0.8 mL/min. Preparatory HPLC was performed on the same system but equipped with a Kromasil 100 C18 5 μM LC precolumn (50 × 20 mm, Agela) and a Kromasil 100 C18 5 μM LC preparative column (250 × 50 mm, Agela) for purification of CB7-HEG-BC (**1**) and CB7-TEG-BC (**2**) at a flow rate of 12 mL/min. All crude samples were dissolved in a mixture of water and ACN (v/v = 3/1) to get a final concentration of 1.5 mg/mL with one drop of TFA to improve the solubility.

Electrospray Ionization Ion Mobility-Mass Spectrometry (ESI-IM-MS). All samples (only for chemosensor **1** and **2**) were dissolved in Milli-Q water to reach a final concentration of 500 μM. The stock solutions were diluted 20 times with Milli-Q water and then further diluted with ACN (v/v = 1/2). About 20 μL formic acid was added to acidify 2 mL diluted samples which were directly infused to a Waters' Synapt G2S HDMS mass spectrometer equipped with ESI source coupled with ion mobility cell. All data was collected in positive ion mode in the mass range m/z 200-2000. Optimized parameters are as follows: flow rate: 5 μL/min, capillary voltage: 1 kV, cone voltage: 20 V, source offset: 30 V, source temperature: 80ºC, desolvation temperature: 200ºC, cone gas flow: 0 L/min, desolvation gas flow: 400 L/min, nebulizer: 2.5 bar. Guest inclusion was confirmed by IMS where additional optimized parameters used are as follows: He flow: 180 mL/min, IMS gas flow: 90 mL/min, trap: 2 mL/min, IMS wave velocity: 850 m/S, wave height: 40 V. Collision cross-sections were calibrated using polyalanine (2+ ions). The mass resolution was 35000 and IMS resolution was 25-30. About 300 spectra acquired over 5 min were averaged for each measurement.

Electrospray Ionization Mass Spectrometry (ESI-MS). Mass spectrum was performed on a microOTOF-Q (208-320 Vac, 50/60 Hz, 1800VA), Bruker. The trace amount of samples (except chemosensor **1** and **2**) were dissolved into 1 mL MeOH and then treated in an ultrasound bath for 30 seconds. All of data were collected in positive mode in the mass range m/z 100-2000. Optimized parameters are as follows: dry temperature: 100°C, dry gas: 3.0 L/min, ion energy: 5.0 eV, collision energy: 10.0 eV, collision RF: 800 Vpp, transfer time: 120 μs, prepuls storage: 10 μs.

Absorbance Spectra. Absorbance spectra were measured at 25°C in Milli-Q water on a Jasco V-730 double-beam UV-Vis spectrophotometer. For UV-Vis absorption experiments, PMMA cuvettes with a light path of 10 mm and dimensions of 10 × 10 mm from Brand with a spectroscopic cut-off at 220 nm were utilized. The samples were equilibrated by using a water thermostatic cell holder STR-812, while the cuvettes were equipped with a stirrer allowing rapid mixing.

Fluorescence Spectra. Steady-state emission spectra and time-resolved emission profiles were recorded on a Jasco FP-8300 fluorescence spectrometer equipped with a 450 W Xenon arc lamp, double-grating excitation and emission monochromators. Emission spectra were corrected for source intensity (lamp and grating) and the emission spectral response (detector and grating) by standard correction curves. All titration and kinetic experiments were carried out at 25°C by using a water thermostatic cell holder STR-812, while the cuvettes were equipped with a stirrer allowing rapid mixing. For fluorescence-based titration experiments, PMMA cuvettes with a light path of 10 mm and dimensions of 10 × 10 mm from Brand with a spectroscopic cut-off at 300 nm were utilized.

Synthesis and Characterization

Synthesis of berberrubine (BC-OH)

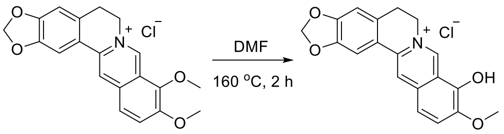

According to literature procedures,[5] berberine hydrochloride (740 mg, 2.0 mmol) was dissolved in 4 mL DMF. After stirring at 160°C for 2 h the solvent was removed under reduced pressure to yield a dark red solid. The crude product was purified by column chromatography (silica, CH_2Cl_2/MeOH (v/v = 10/1)) to give berberrubine as red solid (497 mg, 1.6 mmol, 70 %). ^{1}H NMR (500 MHz, DMSO-d_6) δ 9.07 (s, 1H), 7.98 (s, 1H), 7.61 (s, 1H), 7.21 (d, J = 7.8 Hz, 1H), 6.96 (s, 1H), 6.35 (d, J = 7.8 Hz, 1H), 6.09 (s, 2H), 4.48 (t, J = 6.1 Hz, 2H), 3.72 (s, 3H), 3.04 (t, J = 6.1 Hz, 2H) ppm. ^{13}C NMR (126 MHz, DMSO-d_6) δ 167.5, 150.1, 148.8, 147.7, 146.2, 133.7, 132.5, 129.7, 122.3, 121.7, 120.4, 117.6, 108.7, 105.2, 102.0, 101.3, 56.1, 52.8, 28.0 ppm.

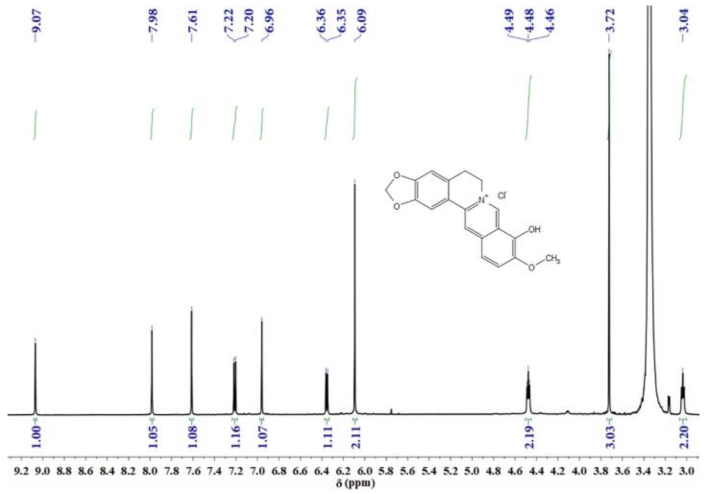

Figure S1. ^{1}H NMR spectrum (500 MHz) of BC-OH in DMSO-d_6.

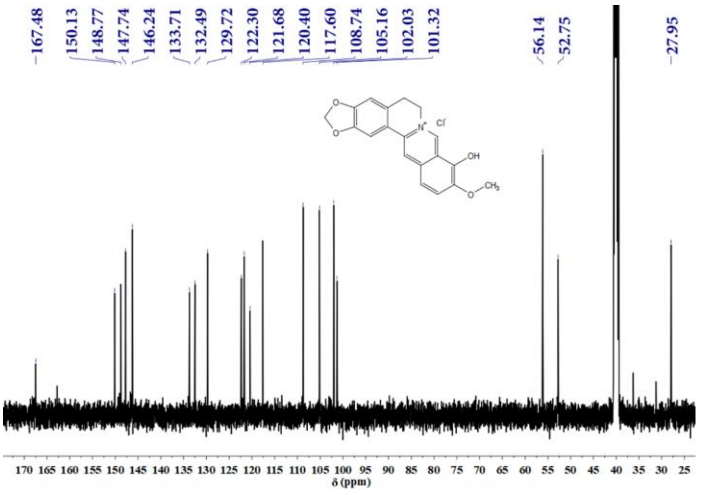

Figure S2. ^{13}C NMR spectrum (126 MHz) of BC-OH in DMSO-d_6.

Synthesis of Dibromo-hexaethylene glycol (Br-HEG-Br)

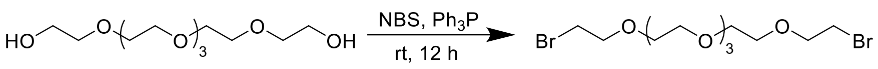

According to literatures procedures,[6] 100 mL CH_2Cl_2 were cooled down to −78°C by using a cooling mixture of dry ice and acetone. NBS (8.72 g, 49.0 mmol) and triphenylphosphine (12.84 g, 49.0 mmol) were added into a round bottom flask under stirring. After 10 min, hexaethylene glycol (4.23 g, 15.0 mmol) was added to the solution and the reaction mixture was stirred overnight. CH_2Cl_2 was removed under reduced pressure and the crude product was purified on a silica column, eluted with hexane/ethyl acetate (v/v = 2/1). The product was obtained as colorless oil (3.98 g, 9.8 mmol, 65 %). ^{1}H NMR (500 MHz, $CDCl_3$) δ 3.81 (t, J = 3.2 Hz, 4H), 3.66 (m, 16H), 3.47 (t, J = 3.2 Hz, 4H) ppm. ^{13}C NMR (126 MHz, $CDCl_3$) δ 71.36, 70.83, 70.75, 70.69, 30.47 ppm.

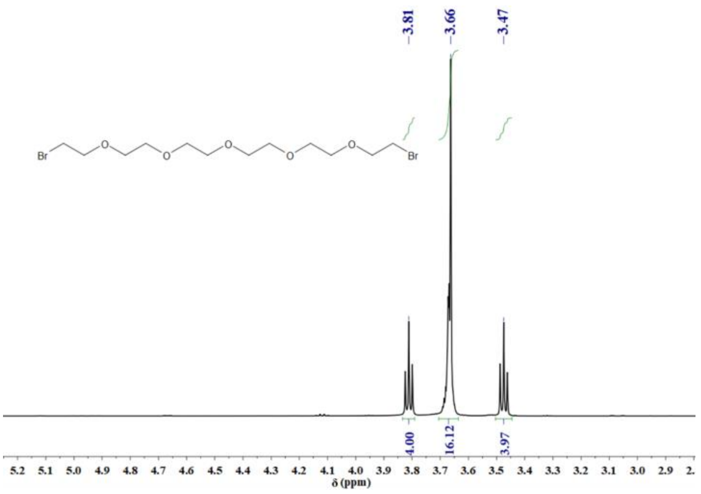

Figure S3. ^{1}H NMR spectrum (500 MHz) of Br-HEG-Br in $CDCl_3$.

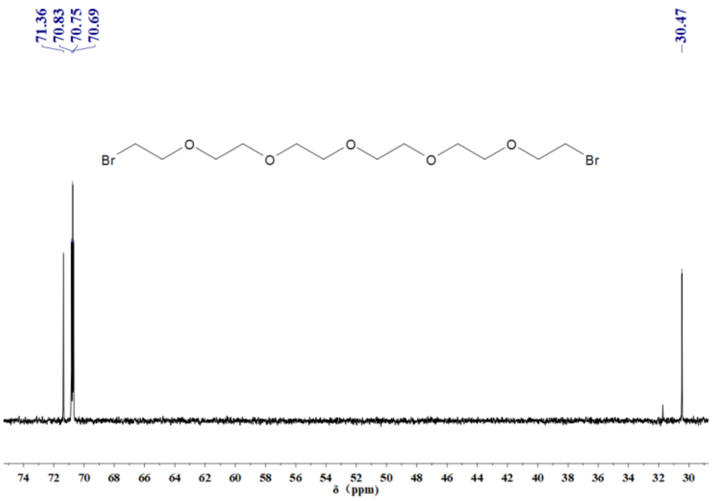

Figure S4. ^{13}C NMR spectrum (126 MHz) of Br-HEG-Br in $CDCl_3$.

Synthesis of berberine-hexaethylene glycol-bromide (BC-HEG-Br)

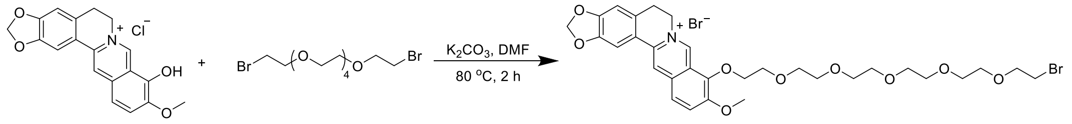

A solution of BC-OH (50.0 mg, 140 µmol), K_2CO_3 (58.0 mg, 420 µmol) and Br-HEG-Br (285 mg, 700 µmol, 180 µL) in 2 mL DMF was heated to 80°C under N_2 for 2 h. Excess of Br-HEG-Br was used to obtain preferentially the mono-substituted product. Afterwards, the mixture was cooled to room temperature and poured in 40 mL Et_2O. The crude product precipitated immediately. The crude product was dissolved in DCM and subjected to an isocratic silica column, eluted with DCM/MeOH (v/v = 10/1), and therefore purified to give a yellow powder of BC-HEG-Br (73.8 mg, 110 µmol, 81 %). ^1H NMR (500 MHz, DMSO-d_6) δ 9.77 (s, 1H), 8.95 (s, 1H), 8.20 (d, J = 9.1 Hz, 1H), 8.01 (d, J = 9.1 Hz, 1H), 7.81 (s, 1H), 7.10 (s, 1H), 6.18 (s, 2H), 4.93 (t, J = 6.2 Hz, 2H), 4.42 (t, J = 4.6 Hz, 2H), 4.07 (s, 3H), 3.80 (t, J = 4.6 Hz, 2H), 3.68 (t, J = 5.8 Hz, 2H), 3.60 - 3.43 (m, 18H), 3.21 (t, J = 6.3 Hz, 2H) ppm. ^{13}C NMR (126 MHz, DMSO-d_6) δ 150.6, 149.9, 147.7, 145.5, 142.5, 137.5, 132.9, 130.7, 126.6, 123.6, 122.0, 120.5, 120.2, 108.5, 105.4, 102.1, 73.1, 70.3, 69.8, 69.7, 69.7, 69.5, 69.5 69.5, 69.4, 69.3, 57.0, 55.5, 39.5, 32.2, 26.4 ppm. ESI-MS: Calcd. for $[M]^+$: 648.1083; found: 648.1792.

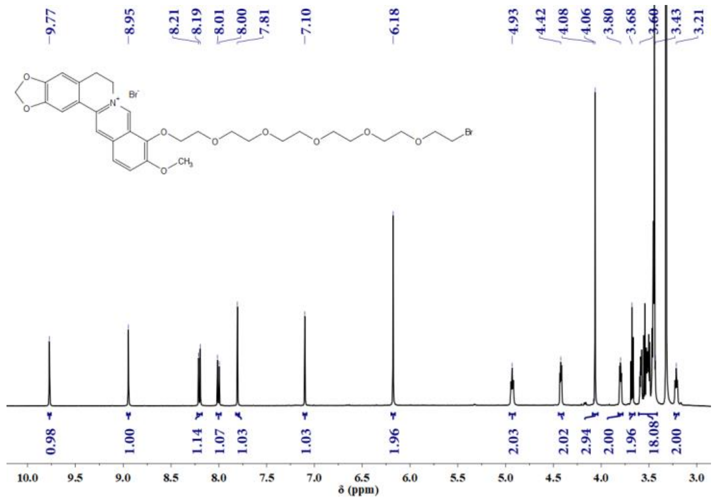

Figure S5. ^{1}H NMR spectrum (500 MHz) of BC-HEG-Br in DMSO-d_6.

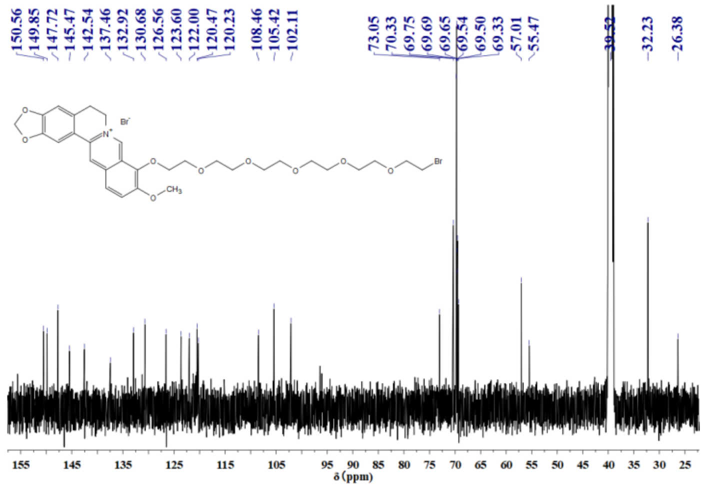

Figure S6. ^{13}C NMR spectrum (126 MHz) of BC-HEG-Br in DMSO-d_6.

Synthesis of berberine-hexaethylene glycol-azide (BC-HEG-N_3)

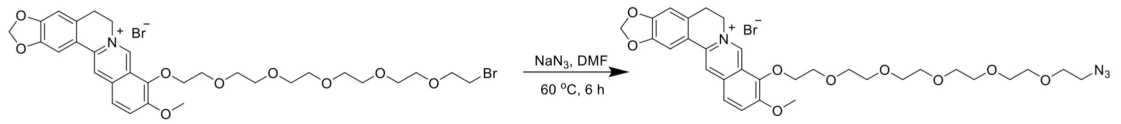

To a solution of BC-HEG-Br (73.8 mg, 100 µmol) in 1 mL DMF, NaN_3 (32.9 mg, 500 µmol) was added at room temperature. After stirring at 60°C for 6 h, the mixture was cooled to room temperature. 50 mL CH_2Cl_2 were added and 3 x 50 mL NaCl sat. solution was added in extraction cycles. The combined organic extracts were dried over anhydrous Na_2SO_4. The crude solution was concentrated and purified by an isocratic silica column eluted with CH_2Cl_2/MeOH (v/v = 10/1) to give BC-TEG-N_3 as yellow powder (66.6 mg, 95.0 µmol, 95 %). ^{1}H NMR (500 MHz, D_2O) δ 9.60 (s, 1H), 8.29 (s, 1H), 7.94 (d, J = 8.7 Hz, 1H), 7.82 (s, J = 8.9 Hz, 1H), 7.30 (s, 1H), 6.90 (s, 1H),

6.06 (s, 2H), 4.84 (t, $J = 6.0$ Hz, 2H), 4.49 (t, $J = 3.1$ Hz, 2H), 4.02 (s, 3H), 3.92 (t, $J = 3.1$ Hz, 2H), 3.72 (t, $J = 3.5$ Hz, 2H), 3.65 – 3.58 (m, 16H), 3.41 (t, $J = 4.3$ Hz, 2H), 3.20 (t, $J = 6.0$ Hz, 2H) ppm. ^{13}C NMR (126 MHz, D_2O) δ 151.8, 150.6, 150.1, 147.7, 144.3, 141.9, 137.6, 133.2, 130.2, 126.3, 123.9, 121.9, 119.9, 108.4, 104.9, 102.3, 72.9, 69.8, 69.6, 69.5, 69.5, 69.5, 69.5, 69.4, 69.4, 69.3, 69.2, 56.6, 56.0, 50.1, 26.7 ppm. ESI-MS: Calcd. for $[M]^+$: 611.2712; found: 611.2696.

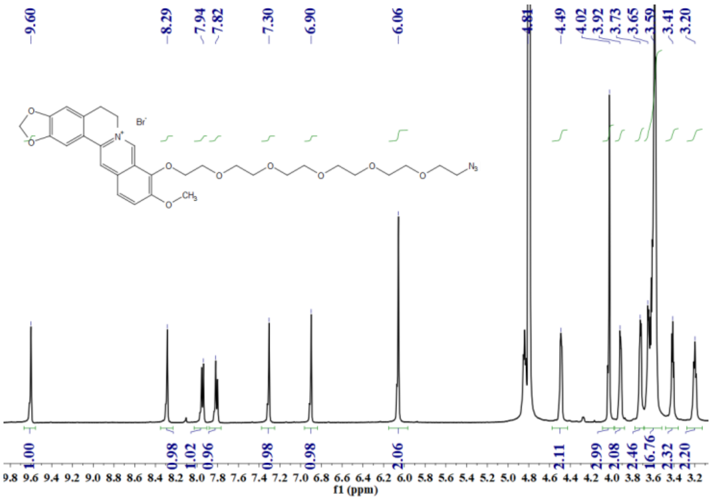

Figure S7. ^1H NMR spectrum (500 MHz) of BC-HEG-N_3 in D_2O.

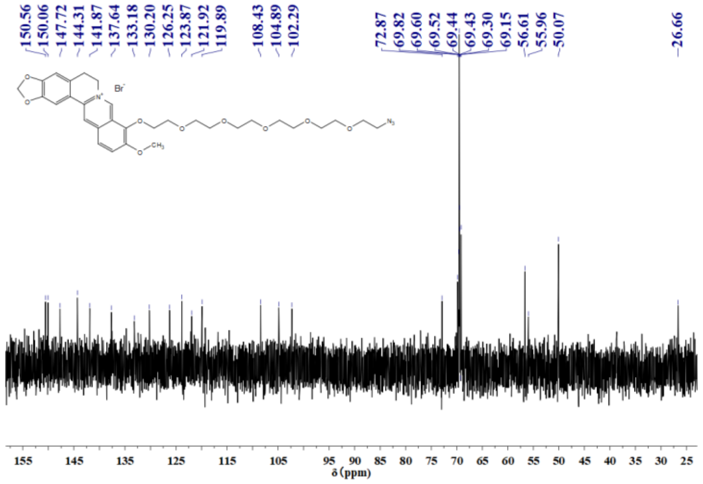

Figure S8. ^{13}C NMR spectrum (126 MHz) of BC-HEG- N_3 in D_2O.

Synthesis of cucurbit[7]uril-HEG-berberine conjugate (1)

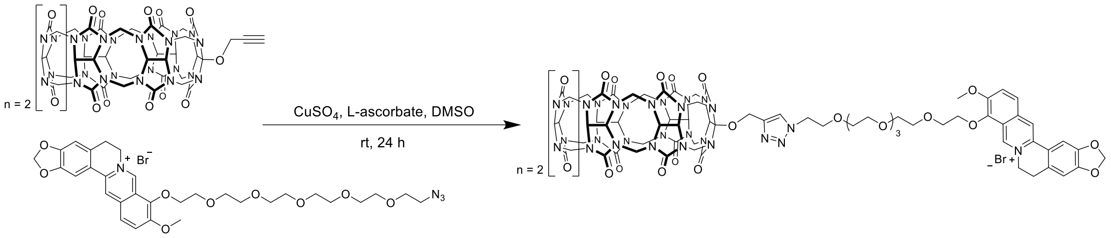

CB7-(Opr)$_1$ (8.7 mg, 7.2 µmol) and BC-HEG-N_3 (9.0 mg, 13.0 µmol) were dispersed in 0.7 mL DMSO. Then, sodium *L*-ascorbate (10.0 mg, 50.0 µmol) was added into 2.8 mL 55% DMSO aqueous solution containing $CuSO_4$ (4.5 mg, 28.0 µmol). These two solutions were mixed and stirred at room temperature for 24 h. Afterwards, 50 mL diethyl ether was added, and the resulting precipitate was washed three times with 25 mL MeOH. Drying under high vacuum afforded a dark solid. The product was purified by HPLC (C18, v (acetonitrile) / v (0.1% TFA aqueous) = 1/3) and obtained as yellow solid (3.6 mg, 1.9 µmol, 27 %). ESI-MS: $[M + Na]^{2+}$ Calcd. for: 925.3076; found: 925.3130. Due to the complex structure a precise characterization by ^{1}H and ^{13}C NMR was not possible. However, as described in the manuscript, formation and purity of the compound were characterized by several methods.

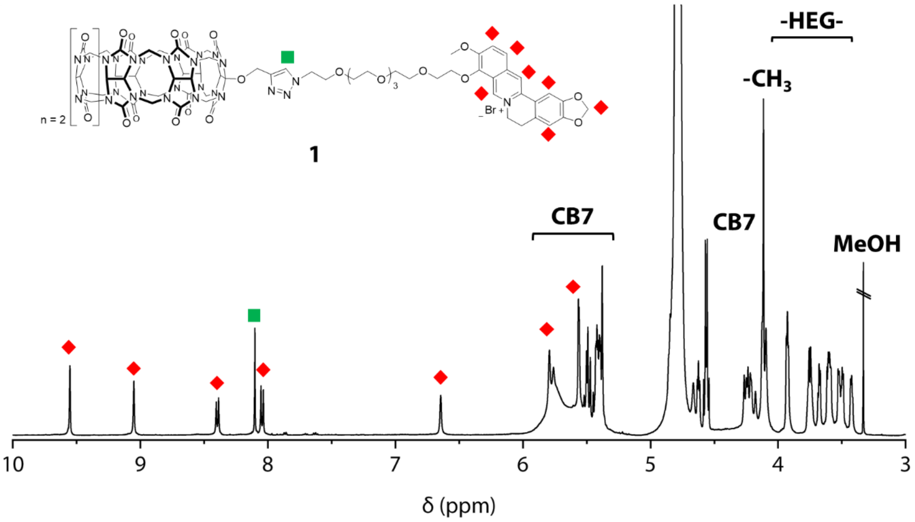

Figure S9. ^{1}H NMR spectrum (500 MHz) of **1** in D_2O.

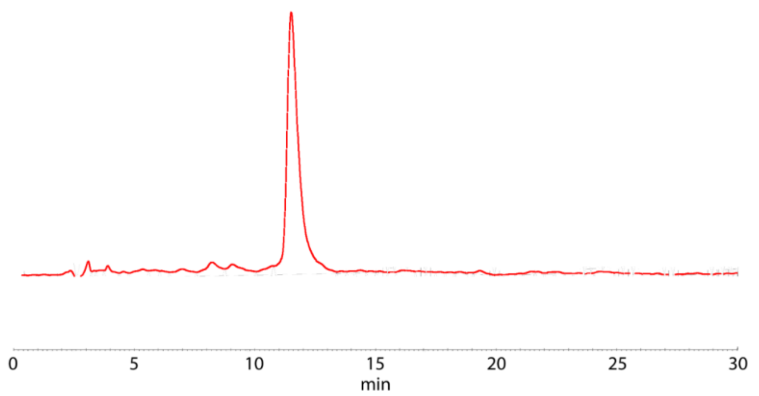

Figure S10. Analytical HPLC trace at 350 nm of **1** using a mixture of 25 % acetonitrile/75 % 0.1 % TFA aqueous as eluents.

Synthesis of Dibromo-tetraethylene glycol (Br-TEG-Br)

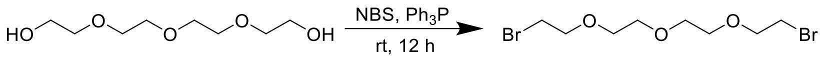

According to literatures procedures, 100 mL CH_2Cl_2 were cooled down to −78°C by using a cooling mixture of dry ice and acetone. NBS (4.36 g, 24.5 mmol) and triphenylphosphine (6.42 g, 24.5 mmol) were added to the reaction mixture under stirring. After 10 min, tetraethylene glycol (2.13 g, 11.0 mmol) was added and the solution was stirred overnight. The solvent was removed under reduced pressure and the crude product was purified by an isocratic silica chromatography with hexane/ethyl acetate (v/v = 4/1) as eluents. The product was obtained as a colorless oil (3.15 g, 9.9 mmol, 90 %). ^{1}H NMR (500 MHz, $CDCl_3$) δ 3.82 (t, J = 3.2 Hz, 4H), 3.68 (s, 8H), 3.48 (t, J = 3.2 Hz, 4H) ppm. ^{13}C NMR (126 MHz, $CDCl_3$) δ 71.2, 70.7, 70.6, 30.4 ppm.

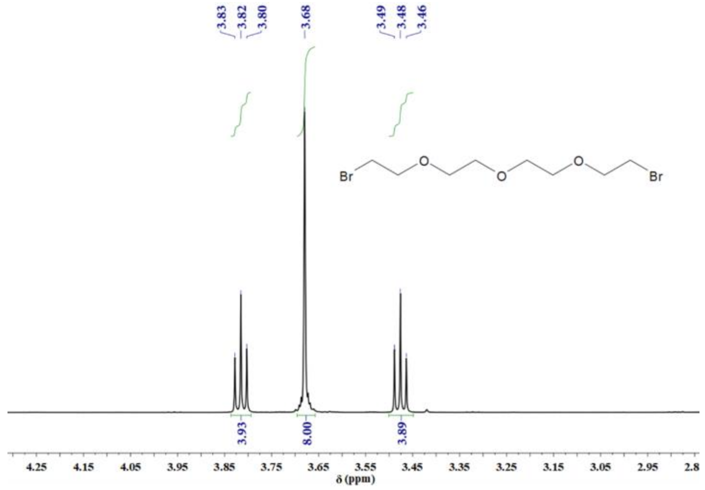

Figure S11. ^{1}H NMR spectrum (500 MHz) of Br-TEG-Br in $CDCl_3$.

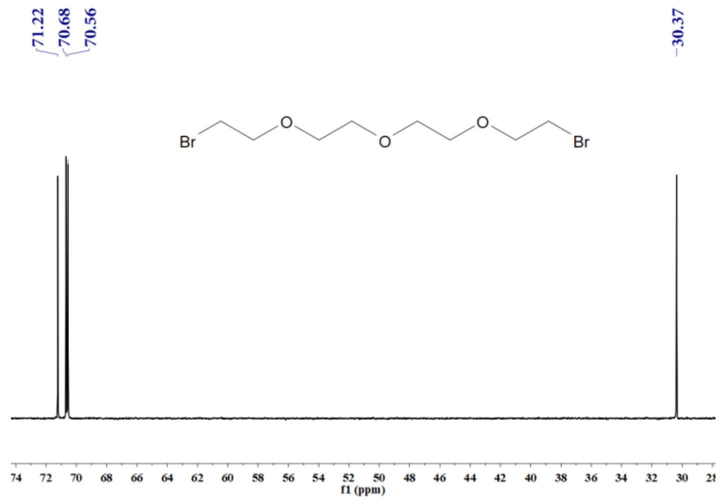

Figure S12. ^{13}C NMR spectrum (126 MHz) of Br-TEG-Br in $CDCl_3$.

Synthesis of berberine-tetraethylene glycol-bromide (BC-TEG-Br)

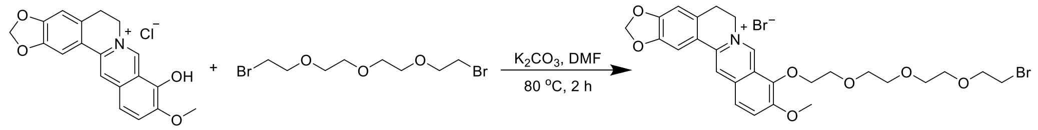

A solution of BC-OH (122 mg, 300 µmol), K_2CO_3 (141 mg, 1.0 mmol) and Br-TEG-Br (546 mg, 1.7 mmol, 1.1 mL) in 2 mL anhydrous DMF was heated to 80°C under nitrogen atmosphere for 2 h. Excess of Br-TEG-Br was used to obtain preferentially the mono-substituted product. Afterwards, the mixture was cooled to room temperature and poured in 40 mL Et_2O. The crude product precipitated immediately. The crude product was dissolved in DCM and purified by an isocratic silica column chromatography with CH_2Cl_2/MeOH (v/v = 10/1) as eluents. After removal of the solvents, BC-TEG-Br was obtained as a yellow powder (69 mg, 100 µmol, 35 %). ^{1}H NMR (500 MHz, DMSO-d_6) δ 9.78 (s, 1H), 8.95 (s, 1H), 8.20 (d, J = 9.2 Hz, 1H), 8.01 (d, J = 9.1 Hz, 1H), 7.81 (s, 1H), 7.10 (s, 1H), 6.18 (s, 2H), 4.93 (t, J = 6.2 Hz, 2H), 4.42 (t, J = 4.4 Hz, 2H), 4.06 (s, 3H), 3.81 (t, J = 4.4 Hz, 2H), 3.65 (t, J = 5.8 Hz, 2H), 3.60 – 3.46 (m, 10H), 3.22 (t, J = 3.1 Hz, 2H) ppm. ^{13}C NMR (126 MHz, DMSO-d_6) δ 151.0, 150.3, 148.2, 145.9, 143.0, 137.9, 133.4, 131.1, 127.0, 124.1, 122.9, 120.9, 120.7, 108.9, 105.9, 102.6, 73.5, 70.8, 70.2, 70.1, 70.0, 69.9, 69.8, 57.5, 55.9, 32.7, 26.9 ppm. ESI-MS: Calcd. for $[M]^+$: 560.1218; found: 560.1365.

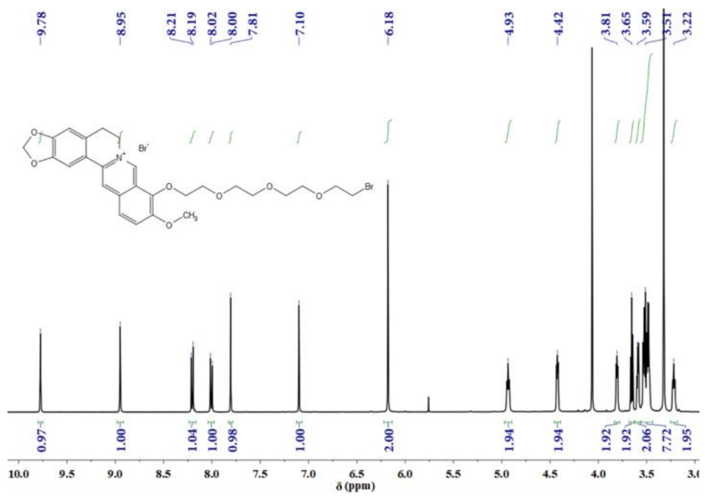

Figure S13. ^{1}H NMR spectrum (500 MHz) of BC-TEG-Br in DMSO-d_6.

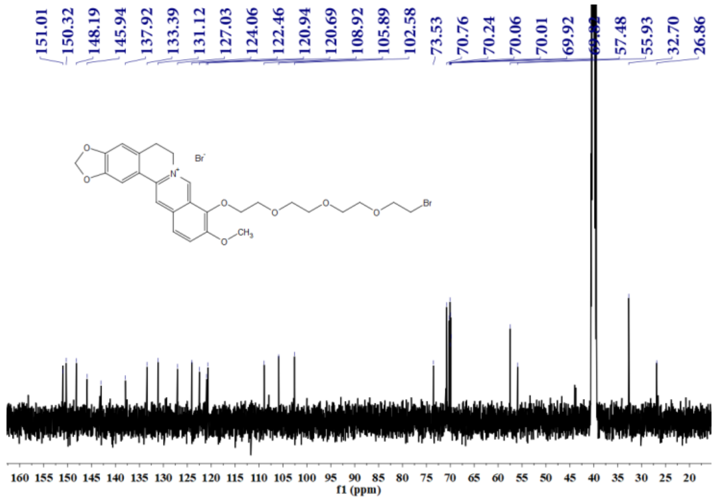

Figure S14. ^{13}C NMR spectrum (126 MHz) of BC-TEG-Br in DMSO-d_6.

Synthesis of berberine-tetraethylene glycol-azide (BC-TEG-N_3)

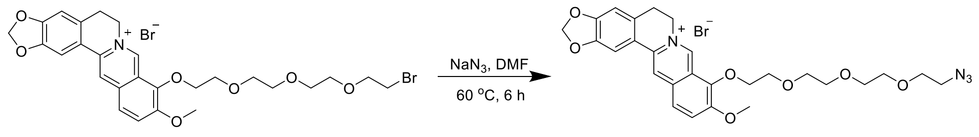

To a solution of BC-TEG-Br (55 mg, 8.6 µmol) in 1 mL DMF, NaN_3 (56 mg, 900 µmol) was added at room temperature. After stirring at 60°C for 6 h the mixture was cooled down to room temperature. 50 mL CH_2Cl_2 were added and 3 x 50 mL NaCl sat. solution was added in extraction cycles. The combined organic extracts were dried under anhydrous Na_2SO_4. The crude solution was

concentrated to 2 mL and purified by an isocratic silica column with CH_2Cl_2/MeOH (v/v = 10/1) as eluents to obtain BC-TEG-N_3 after solvent removal as yellow powder (50 mg, 8.3 μmol, 96 %). ^{1}H NMR (500 MHz, DMSO-d_6) δ 9.78 (s, 1H), 8.95 (s, 1H), 8.20 (d, J = 9.2 Hz, 1H), 8.01 (d, J = 9.1 Hz, 1H), 7.81 (s, 1H), 7.10 (s, 1H), 6.18 (s, 2H), 4.93 (t, J = 6.2 Hz, 2H), 4.42 (t, J = 4.4 Hz, 2H), 4.06 (s, 3H), 3.81 (t, J = 4.4 Hz, 2H), 3.60 – 3.48 (m, 12H), 3.22 (t, J = 3.1 Hz, 2H) ppm. ^{13}C NMR (126 MHz, DMSO-d_6) δ 150.6, 149.9, 147.7, 145.5, 142.6, 137.5, 133.0, 130.7, 126.6, 123.6, 122.0, 120.3, 120.3, 108.5, 105.5, 102.1, 73.1, 69.8, 69.6, 69.6, 69.5, 69.4, 69.2, 57.0, 55.5, 50.0, 26.4 ppm. ESI-MS: Calcd. for $[M]^+$: 523.2187; found: 523.2245.

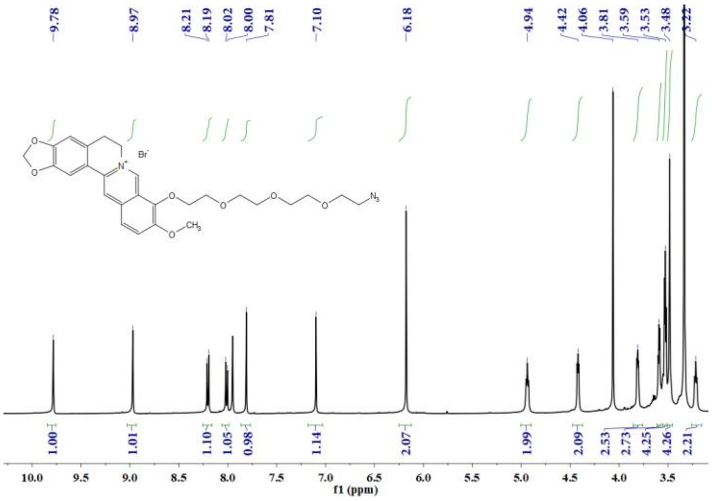

Figure S15. ^{1}H NMR spectrum (500 MHz) of BC-TEG-N_3 in DMSO-d_6.

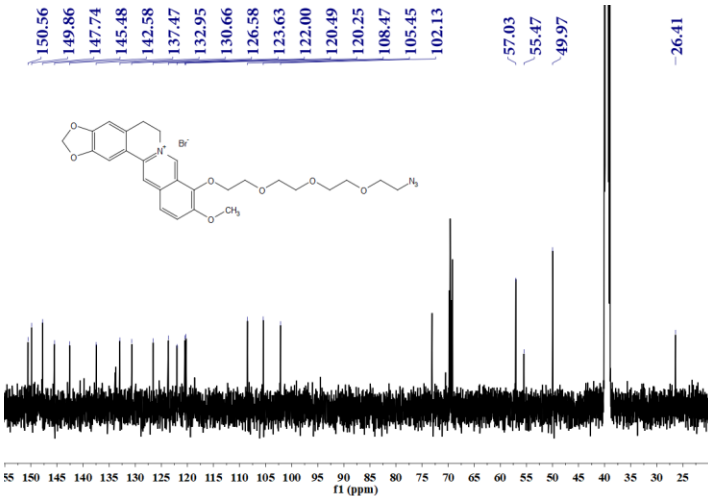

Figure S16. ^{13}C NMR spectrum (126 MHz) of BC-TEG-N_3 in DMSO-d_6.

Synthesis of cucurbit[7]uril-TEG-berberine conjugate (2)

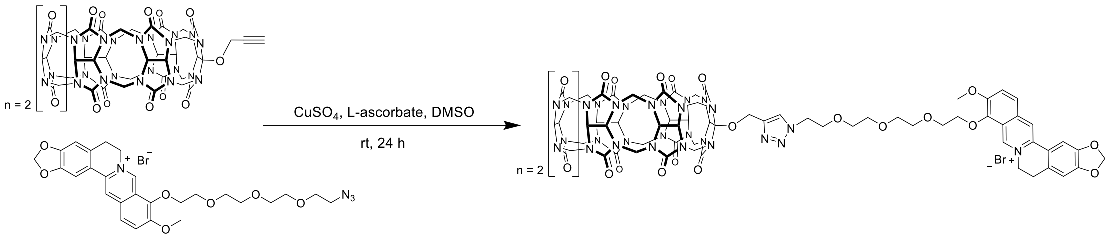

CB7-(Opr)$_1$ (8.7 mg, 7.2 µmol) and BC-TEG-N_3 (7.9 mg, 13.0 µmol) were dissolved in 700 µL DMSO. In a second flask, Sodium *L*-ascorbate (10.0 mg, 50.0 µmol) was added to 2.8 mL 55% DMSO aqueous solution containing $CuSO_4$ (4.5 mg, 28.0 µmol). The two solutions were mixed and stirred at room temperature for 24 h. Afterwards, 50 mL diethyl ether was added and the resulting precipitate was washed three times with 25 mL MeOH. Drying under high vacuum afforded a dark solid. The product was purified by HPLC (C18, v(acetonitrile) / v (0.1% TFA aq.) = 1/3) and obtained as yellow solid (1.2 mg, 690 nmol, 10 %). ESI-MS: $[M+Na]^{2+}$ Calcd. for: 881.2814; found: 881.2889. Due to the complex structure a precise characterization by ^1H and ^{13}C NMR was not possible. However, as described in the manuscript, formation and purity of the compound were characterized by several methods.

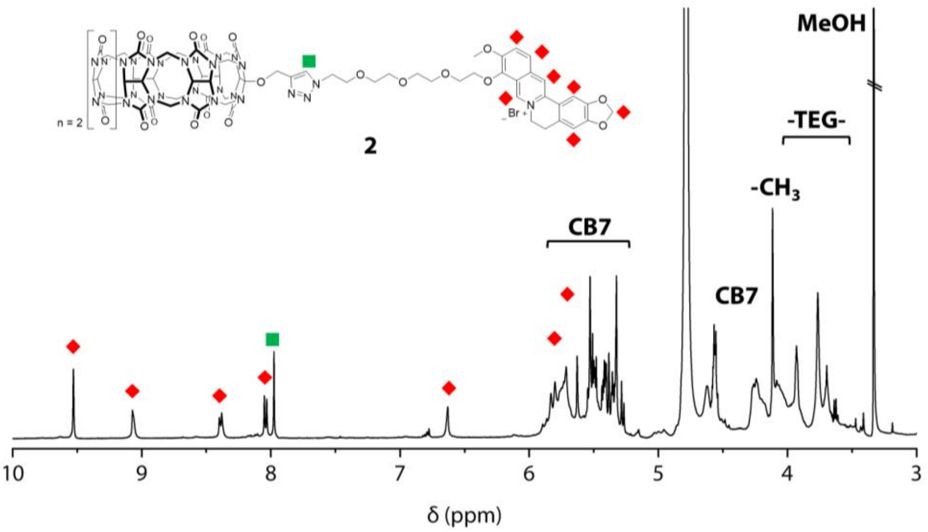

Figure S17. ^1H NMR spectrum (500 MHz) of **2** in D_2O.

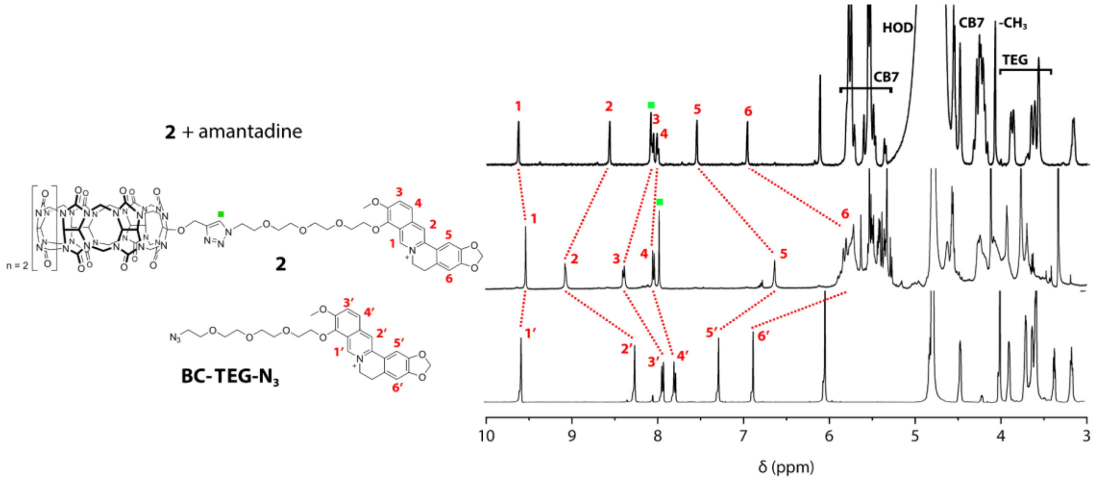

Figure S18. Overlay of ^1H NMR spectrum (500 MHz, D_2O) of BC-TEG-N_3 (bottom), chemosensor **2** (middle) and chemosensor **2** under the addition of one equivalent of amantadine (top).

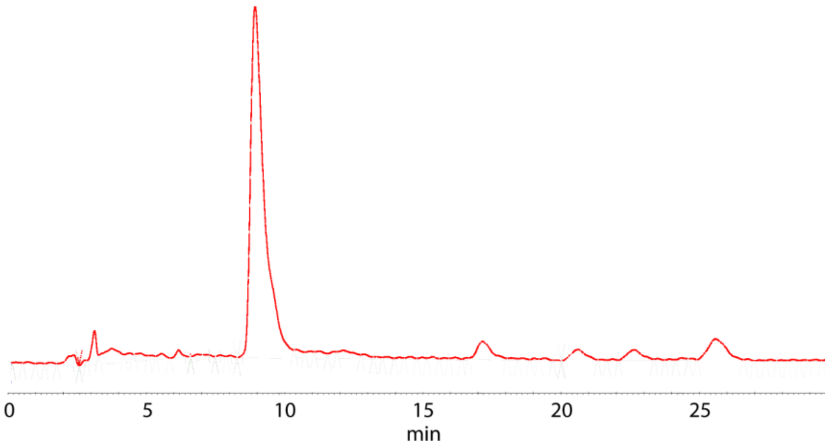

Figure S19. Analytical HPLC trace at 350 nm of **2** using a mixture of 25 % acetonitrile/75 % 0.1 % TFA aqueous for elution.

Synthesis of *N*-adamantyl-4-methylpyridine (Ad-MePy)

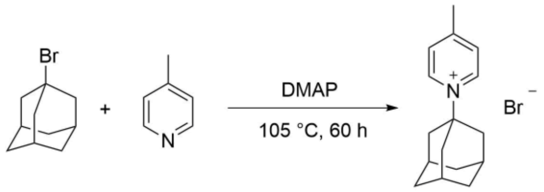

According to the literatures procedures,[7] 1-bromoadamantane (2.15 g, 10.0 mmol), 4-(dimethylamino) pyridine (60.0 mg, 500 μM) and picoline (4.67 g, 50.2 mmol, 4.82 mL) were mixed under nitrogen atmosphere. After the addition of 180 μL distilled water (180 mg, 10.0 mmol) the mixture was refluxed for 60 h. Afterwards, the mixture was cooled down to room temperature and slowly poured into chloroform. A white precipitate was formed immediately and washed several times with chloroform. The product was dried and obtained as a white solid. (2.30 g, 7.49 mmol, 75 %). ^{1}H NMR (500 MHz, D_2O) δ 8.89 (d, J = 6.9 Hz, 2H), 7.86 (d, J = 6.6 Hz, 2H), 2.63 (s, 3H), 2.36 (s, 3H), 2.29 (d, J = 2.6 Hz, 6H), 1.81 (dd, J = 28.3, 12.5 Hz, 6H) ppm. ^{13}C NMR (126 MHz, D_2O) δ 159.2, 139.8, 128.2, 68.6, 41.7, 34.6, 29.8, 20.9 ppm. ESI-MS: Calcd. for $[M]^+$: 228.1447; found: 228.1739.

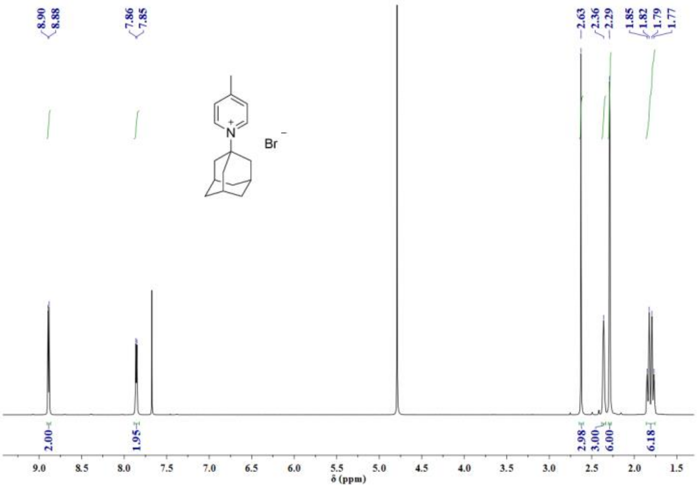

Figure S20. ^{1}H NMR spectrum (500 MHz) of Ad-MePy in D_2O.

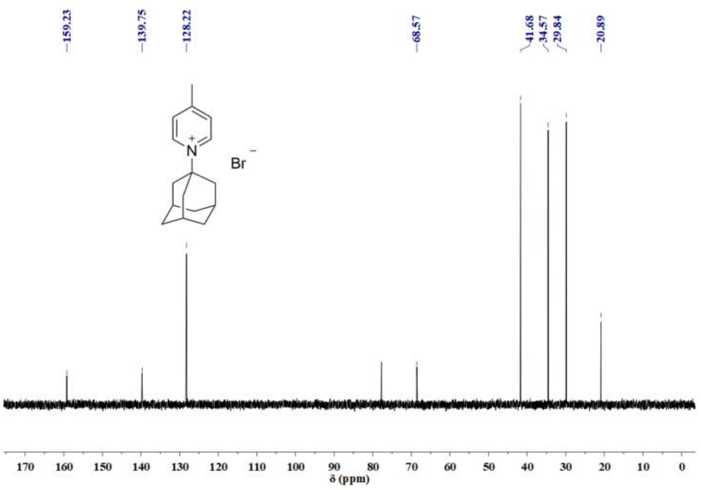

Figure S21. ^{13}C NMR spectrum (126 MHz) of Ad-MePy in D_2O.

Synthesis of *trans*-4-[4-(Dimethylamino)styryl]-1-adamantylpyridinium bromide (DASAP)

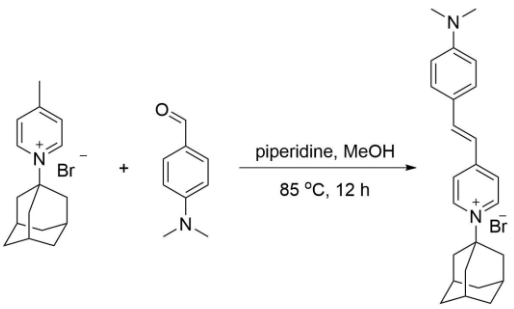

Ad-MePy (250 mg, 814 μM) was dissolved in MeOH (3 mL). To this solution, 4-dimethylamino benzaldehyde (121 mg, 814 μM) and 2-3 drops of piperidine were added and the reaction mixture was heated to 85°C and kept stirring overnight under nitrogen atmosphere. Afterwards, the mixture was cooled down to room temperature and 10 mL of ethyl acetate was added. After storage at 5°C overnight, the precipitate was collected and washed three times with ethyl acetate, one time with hexane and one time with diethyl ether. The residue was then recrystallized from a mixture of dichloromethane and ethyl acetate (v/v =1/2) to give a red-purple solid (108 mg, 247 μmol, 30 %). In analogy to the commercial dye *trans*-4-[4-(Dimethylamino)styryl]-1-methylpyridinium iodide that is commonly abbreviated as DASP, we suggest the abbreviation **DASAP** for the herein introduced dye *trans*-4-[4-(Dimethylamino)styryl]-1-adamantylpyridinium bromide. ^{1}H NMR (500 MHz, MeOH-d_3) δ 8.85 (s, 2H), 7.98 (d, J = 6.3 Hz, 2H), 7.85 (d, J = 15.8 Hz, 1H), 7.62 (d, J = 8.1 Hz, 2H), 7.10 (d, J = 15.9 Hz, 1H), 6.79 (d, J

= 7.4 Hz, 2H), 3.07 (s, 6H), 2.42 – 2.22 (m, 9H), 1.87 (s, 6H) ppm. ^{13}C NMR (126 MHz, MeOH-d_3) δ 155.9, 154.0, 144.4, 141.0, 131.6, 124.2, 123.6, 117.6, 113.1, 68.7, 43.0, 40.2, 36.2, 31.6 ppm. ESI-MS: Calcd. for $[M]^+$: 359.2482; found: 359.2506.

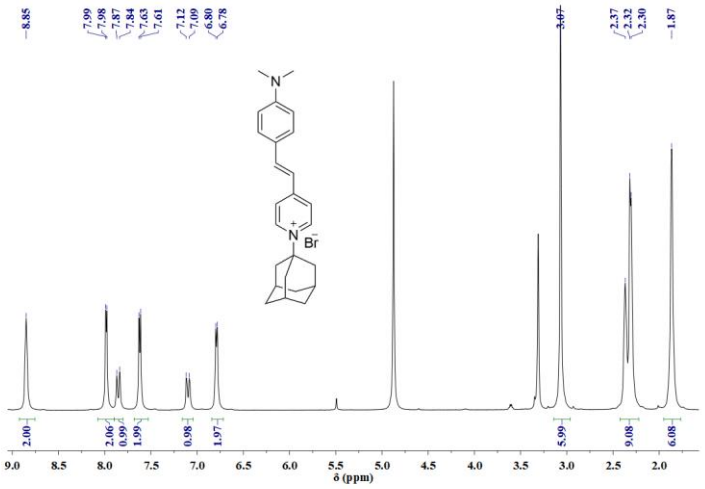

Figure S22. ^{1}H NMR spectrum (500 MHz) of DASAP in MeOH-d_3.

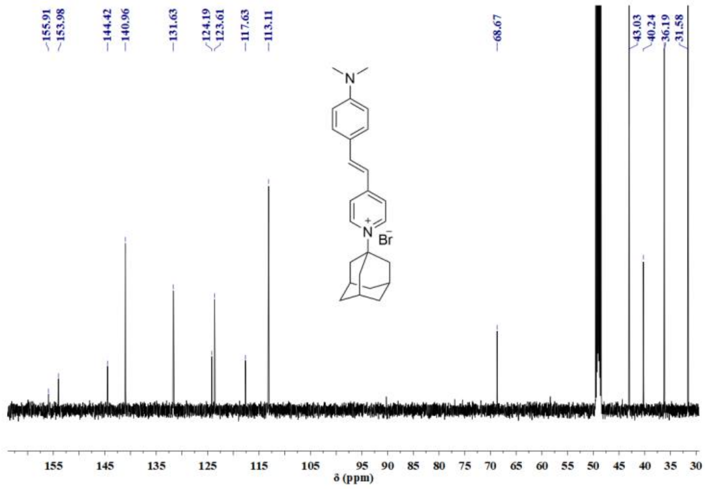

Figure S23. ^{13}C NMR spectrum (126 MHz) of DASAP in MeOH-d_3.

Ion mobilogram and ESI-MS spectra of 1 and 2

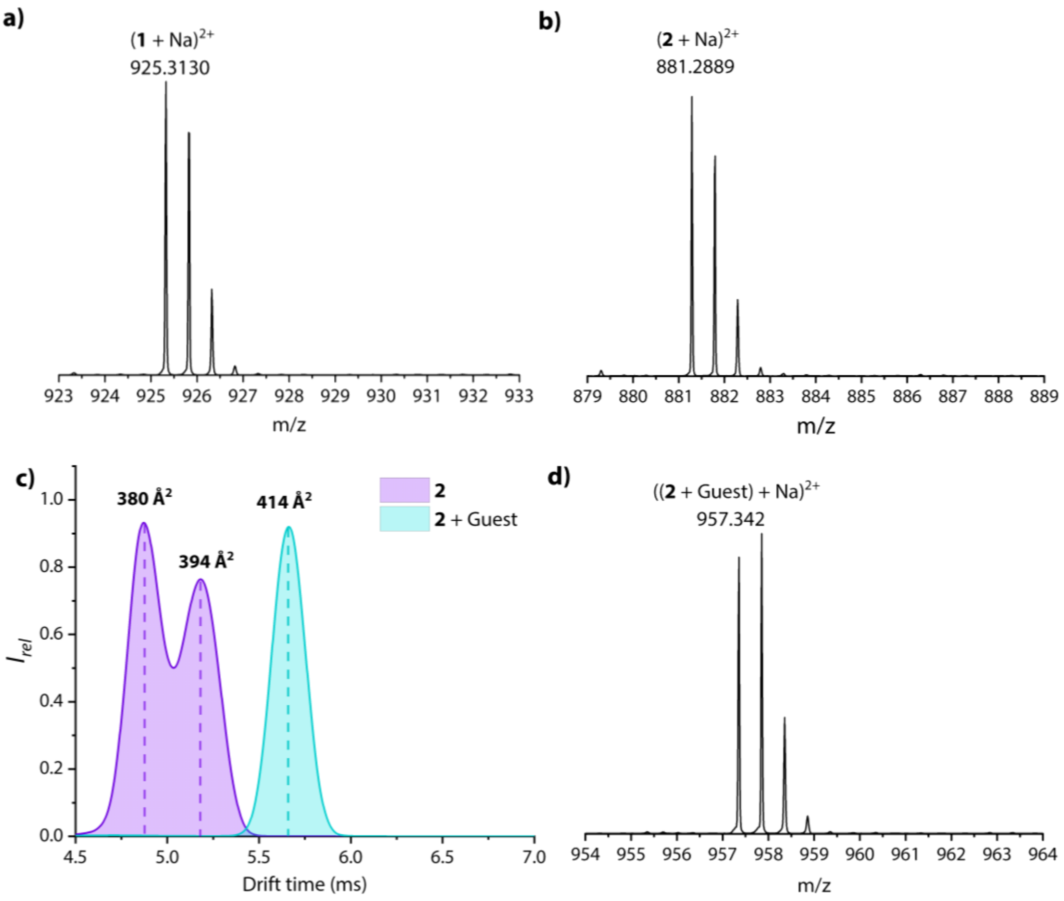

Figure S24. ESI-MS of a) **1** and b) **2** in the absence of 1-adamantanol in a mixture of water and acetonitrile (v/v = 2/1) in positive ion mode; c) Ion mobilogram of **2** in the presence of 1- adamantanol showing a significant increase in collision cross section (CCS) for the inclusion complex confirming successful inclusion of the 1-adamantanol guest. d) ESI-MS of **2** in the presence of 1-adamantanol in a mixture of water and acetonitrile (v/v = 2/1) in positive ion mode.

Table S1. Assigned mass peaks with formula, experimental mass, drift time and CCS.

Ion	Formula	m/z	Drift time (ms)	CCS (Å^2)
$(1 + Na)^{2+}$	$C_{76}H_{83}O_{24}N_{32}Na$	925.299	5.62	412
$((1 + Guest) + Na)^{2+}$	$C_{86}H_{99}O_{25}N_{32}Na$	1001.368	5.94	424
$(2 + Na)^{2+}$	$C_{72}H_{75}O_{22}N_{32}Na$	881.289	4.87/5.17	397
$((2 + Guest) + Na)^{2+}$	$C_{82}H_{92}O_{23}N_{32}Na$	957.324	5.66	414

Fluorescence spectrum analysis

Chemical structures of analytes and reference dyes

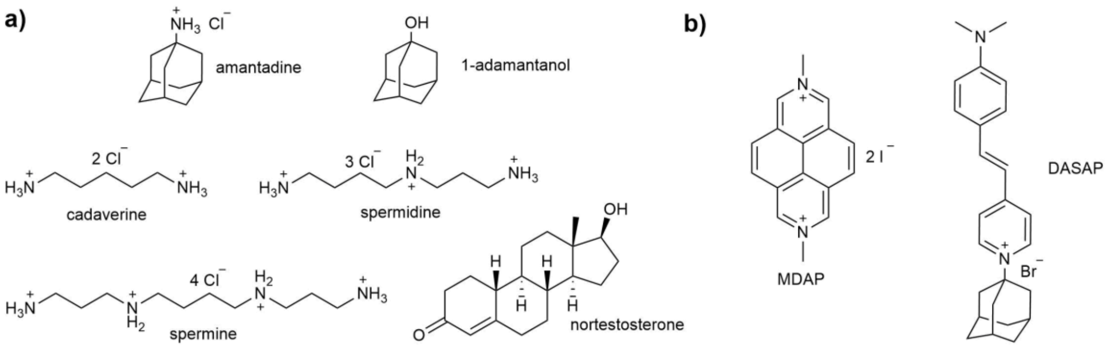

Figure S25. Chemical structures of (a) guests and (b) dyes.

Titration of cucurbit[7]uril to berberine and its derivatives

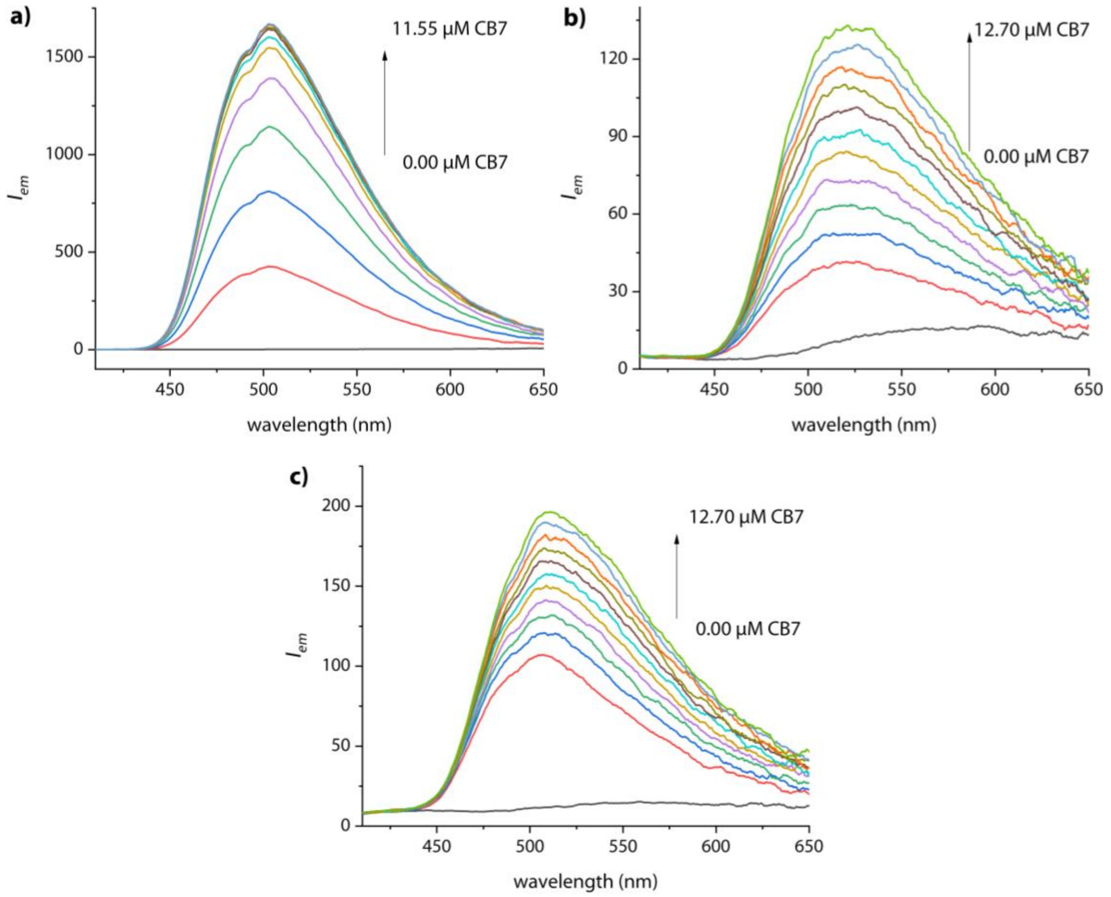

Figure S26. Emission spectra of 5 μM berberine (a), 5 μM BC-HEG-N_3 (b) and 5 μM BC-TEG-N_3 (c), upon addition of CB7 in water, λ_{ex}= 350 nm. Interval time between each titration step: 50 sec.

Titration of cucurbit[7]uril to DASAP

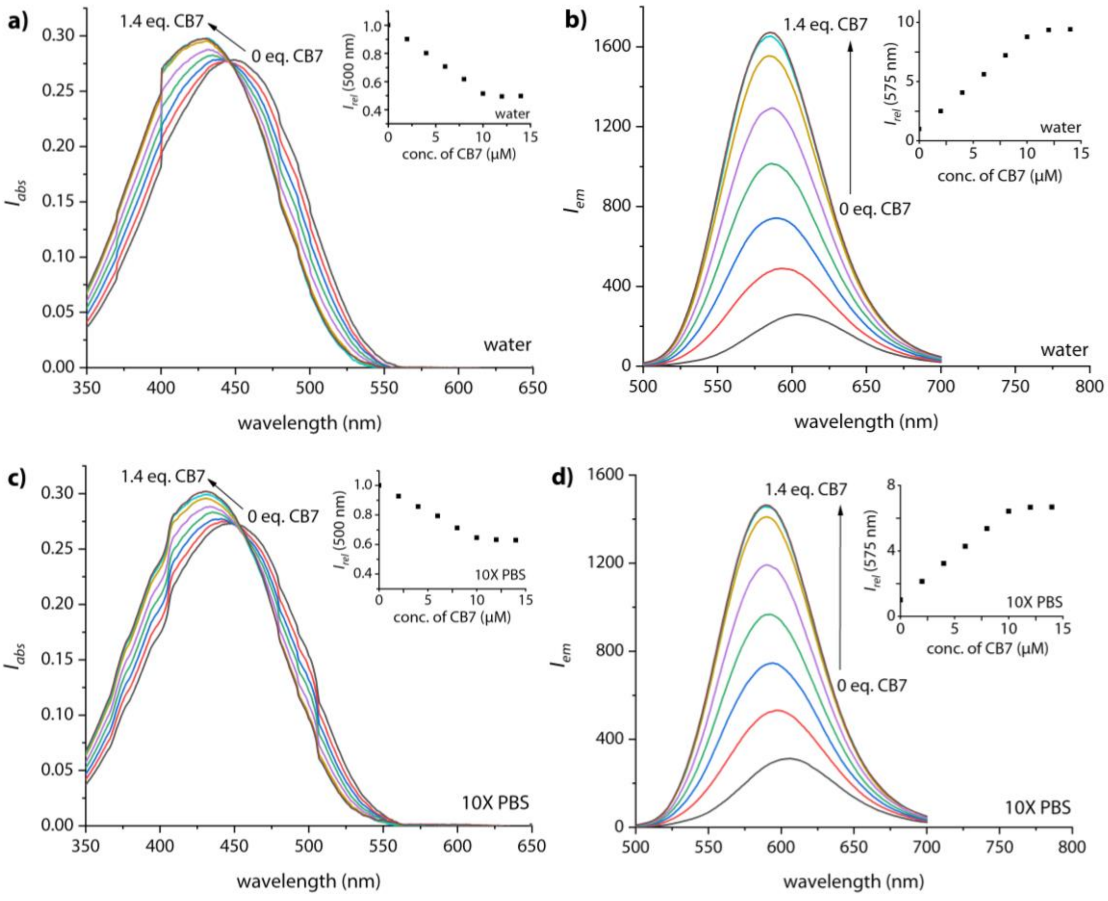

Figure S27. UV-Vis absorption spectra of 10 μM DASAP in water (a) and 10X PBS (c) at 25°C upon addition of CB7. Inset: plot of normalized absorption intensity at 500 nm. Emission spectra of 10 μM DASAP upon addition of CB7 in water (b) and 10X PBS (d) at 25°C, λ_{ex} = 450 nm. Inset: plot of normalized emission intensity at 575 nm. Interval time between each titration step 50 sec.

Fluorescence kinetic traces of amantadine addition to CB7⊃DASAP

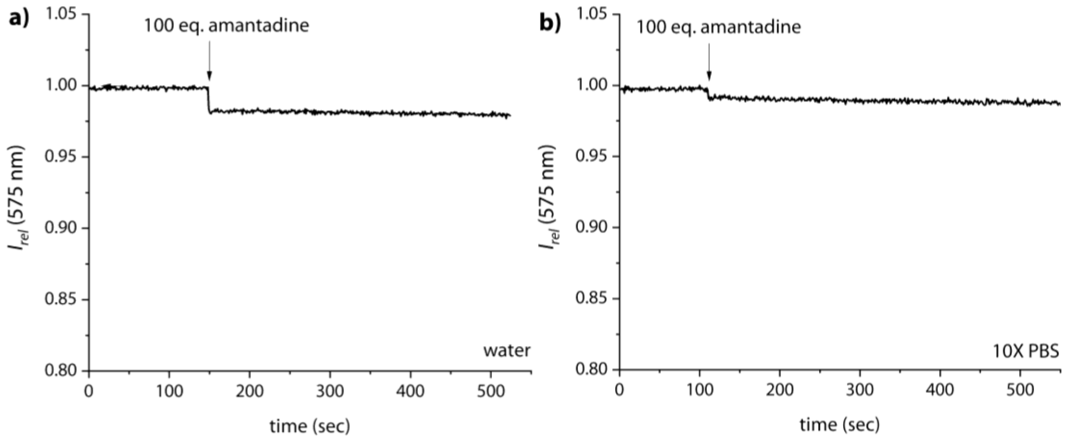

Figure S28. Normalized fluorescence-based kinetic traces monitored at 575 nm of 5 µM CB7⊃DASAP and 500 µM (100 equivalents) amantadine in water (a) and 10X PBS (b) at 25°C, λ_{ex} = 450 nm.

Fluorescence kinetic traces of DASAP to CB7⊃amantadine

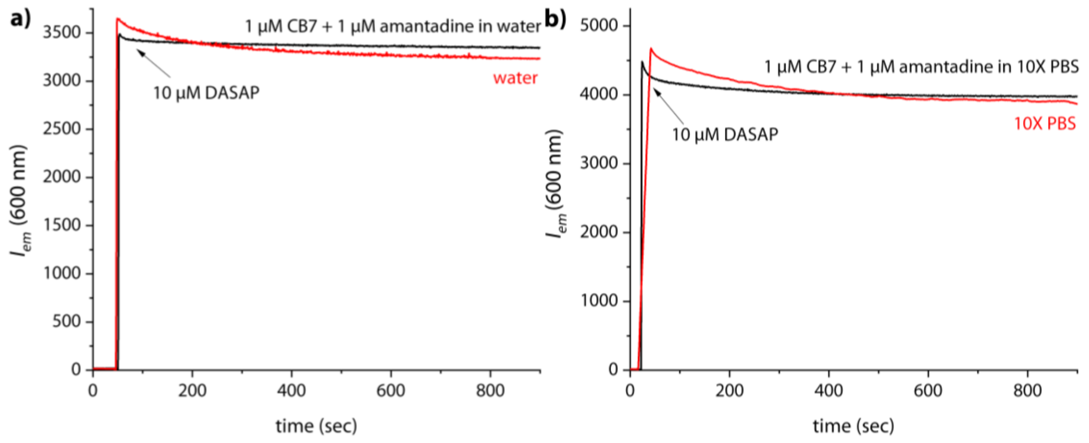

Figure S29. (a) Fluorescence-based kinetic traces at 600 nm of 10 µM DASAP into water (red) and 1 µM CB7⊃amantadine (0.1 eq.) in water (black), λ_{ex} = 530 nm; (b) Fluorescence-based kinetic traces at 600 nm of 10 µM DASAP into 10X PBS (red) and 1 µM CB7⊃amantadine (0.1 eq.) in 10X PBS (black), λ_{ex} = 530 nm.

Fluorescence titration and kinetic traces of cucurbit[7]uril to MDAP

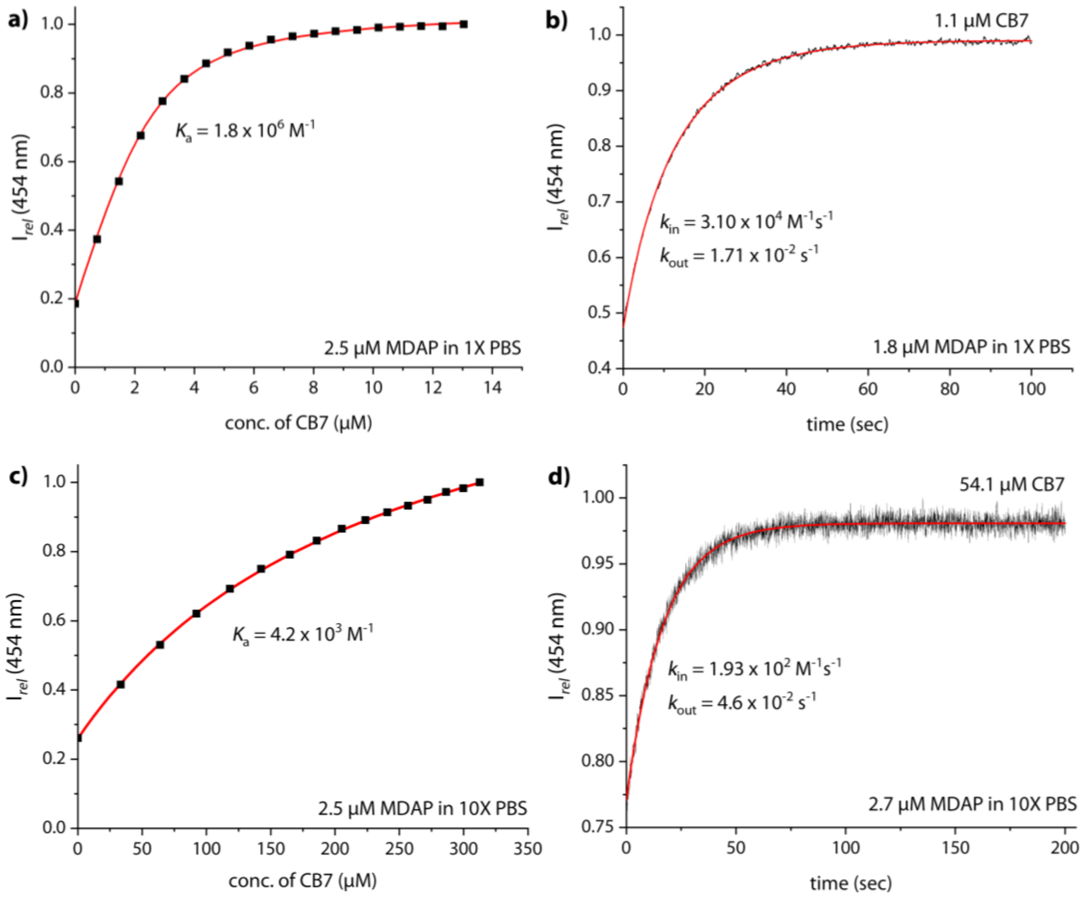

Figure S30. Fitting plot of normalized emission intensity at 454 nm of 2.5 µM MDAP in 1X PBS (a) and 10X PBS (c) at 25°C, upon addition of CB7, λ_{ex} = 339 nm. Interval time between titration steps: 100 sec; Fitting plot of normalized fluorescence-based kinetic traces of 1.8 µM MDAP with 1.1 µM CB7 in 1X PBS (b) and 2.7 µM MDAP with 54.1 µM CB7 in 10X PBS (d) at 25°C, λ_{ex} = 339 nm. The excess CB7 addition in case of 10X PBS was used to ensure a significant intensity change.

Absorption and emission spectra of chemosensor 2

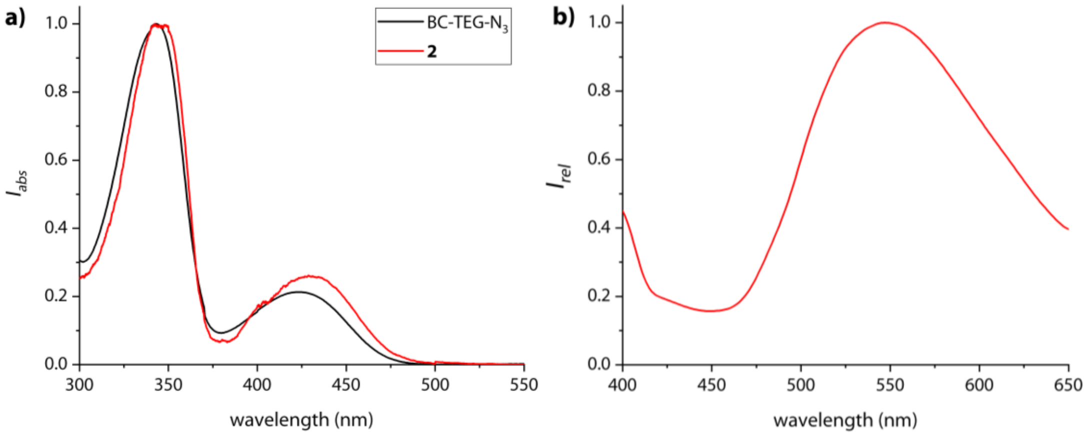

Figure S31. (a) Normalized UV-Vis absorption spectra of 5 μM BC-TEG-N_3 and of chemosensor **2** in water at 25°C. (b) Normalized emission spectra of 1 μM **2** in water at 25°C, λ_{ex} = 350 nm.

Fluorescence titration of NaCl to 1 and 2

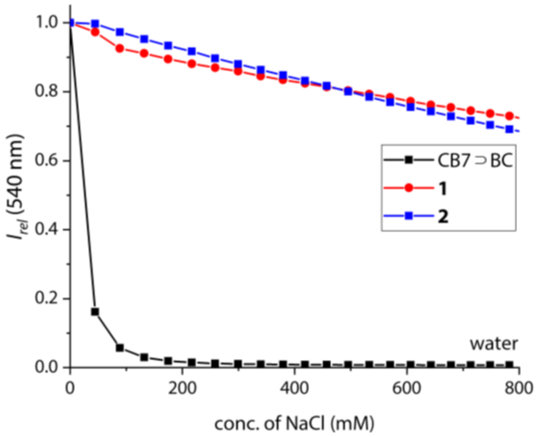

Figure S32. Plot of normalized emission intensity at 540 nm of 1 μM CB7⊃BC complex (black), **1** (red) and **2** (blue) in water at 25°C, upon addition of NaCl, λ_{ex}=350 nm. Interval time between titration steps: 100 seconds.

Fluorescence titration of amantadine to 1 and 2

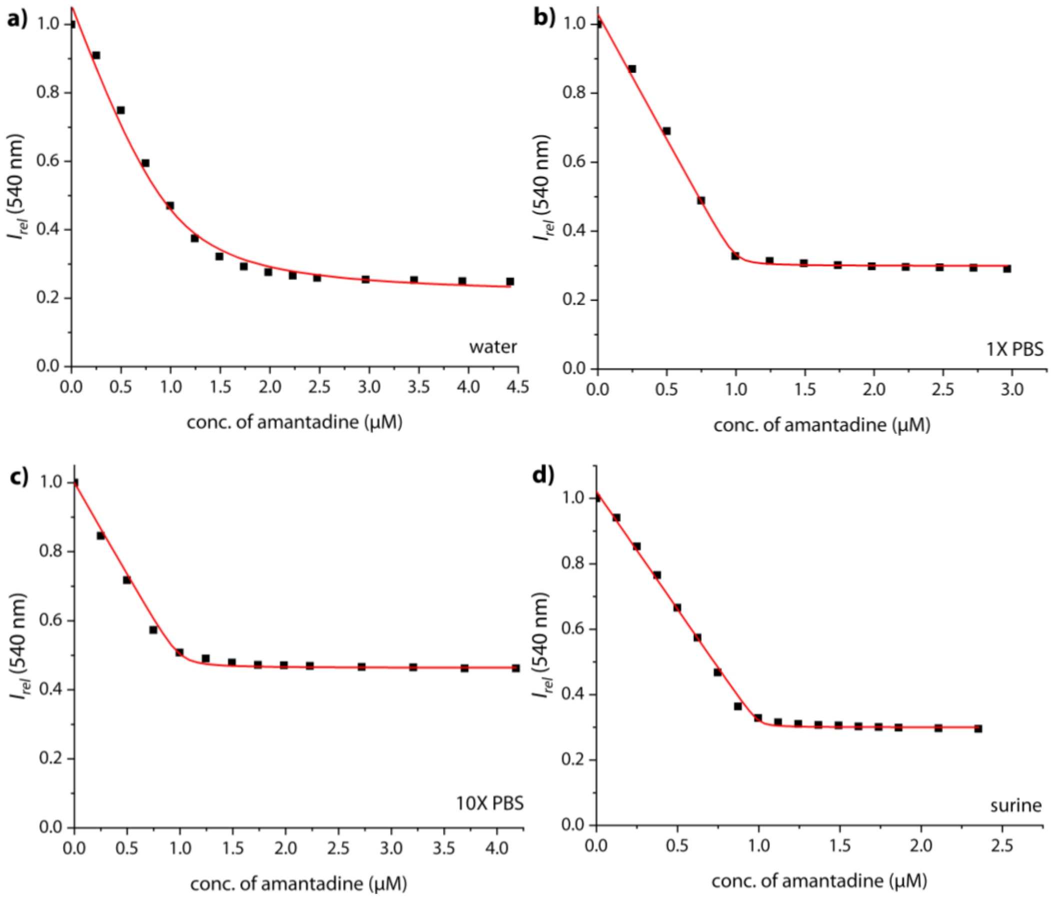

Figure S33. Fitting plots of normalized emission intensity at 540 nm of 1 μM **1** in water (a), 1X PBS (b), 10X PBS (c) and surine (d) at 25°C, upon addition of amantadine, λ_{ex} = 350 nm. Intervals between titration steps: 500 seconds in water, 200 seconds in 1X PBS/10X PBS/surine.

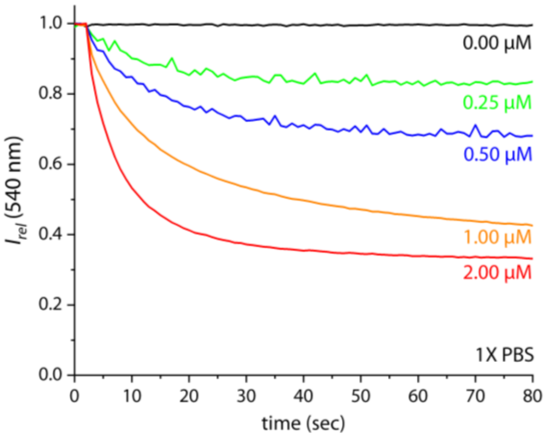

Figure S34. Normalized fluorescence-based kinetic traces at varied concentrations of amantadine and 1 μM **1** in 1X PBS at 25°C. Concentration of amantadine for each curve (top to bottom, black to red): 0, 0.25, 0.50, 1 and 2 μM, λ_{ex} = 350 nm.

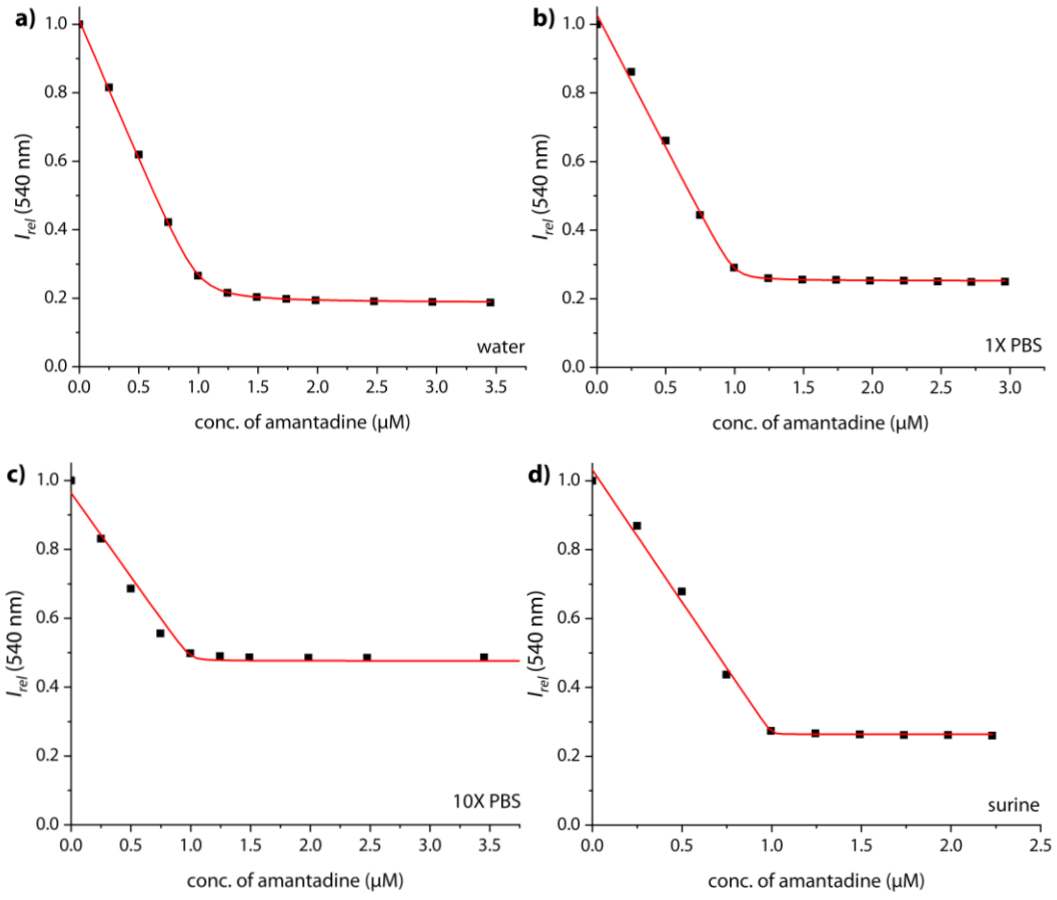

Figure S35. Fitting plot of normalized emission intensity at 540 nm of 1 μM **2** in water (a), 1X PBS (b), 10X PBS (c) and surine (d) at 25°C, upon addition of amantadine, λ_{ex} = 350 nm. Interval time between titration steps: 500 seconds in water, 200 seconds in 1X PBS/10X PBS/surine.

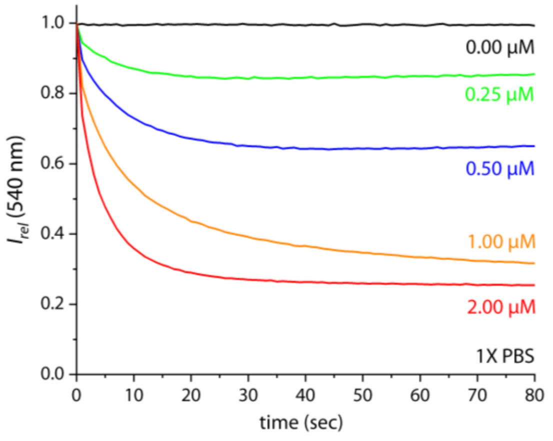

Figure S36. Normalized fluorescence-based kinetic traces at varied concentrations of amantadine and 1 μM **2** in 1X PBS at 25°C. Concentration of amantadine for each curve (top to bottom, black to red): 0, 0.25, 0.50, 1 and 2 μM, λ_{ex} = 350 nm.

The normalized fluorescence plots of the amantadine titrations clearly show behaviors characterized by two straight lines intersecting at the equivalence point, which was reached exactly upon addition of 1.0 eq. of amantadine (for 1 µM chemosensor). This behavior indicates quantitative complex formation at the lower micromolar concentration range used in these experiments. For the here used setup, the binding constant between amantadine and the berberine-based chemosensors is too high to be determined directly by fluorescence titrations. The inset of simulated titration curves indicates for $K_a > 10^8$ M^{-1}.

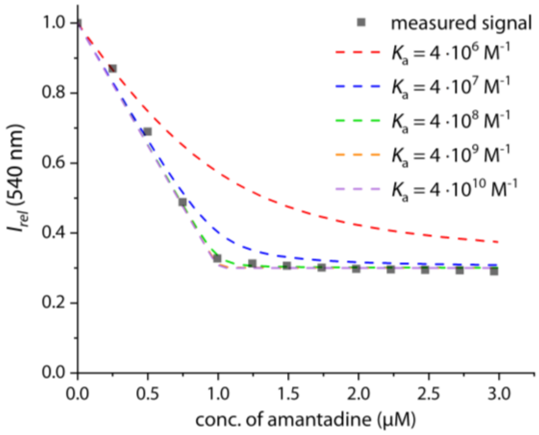

Figure S37. Fitting simulation of emission intensity at 540 nm of 1 µM of chemosensor **1** or **2** upon addition of amantadine for several binding affinities (dashed lines). In addition, the measured data for the titration of amantadine to 1 µM **1** in 1X PBS buffer at 25°C is shown, λ_{ex} = 350 nm.

Fluorescence titration of cadaverine to 1 and 2

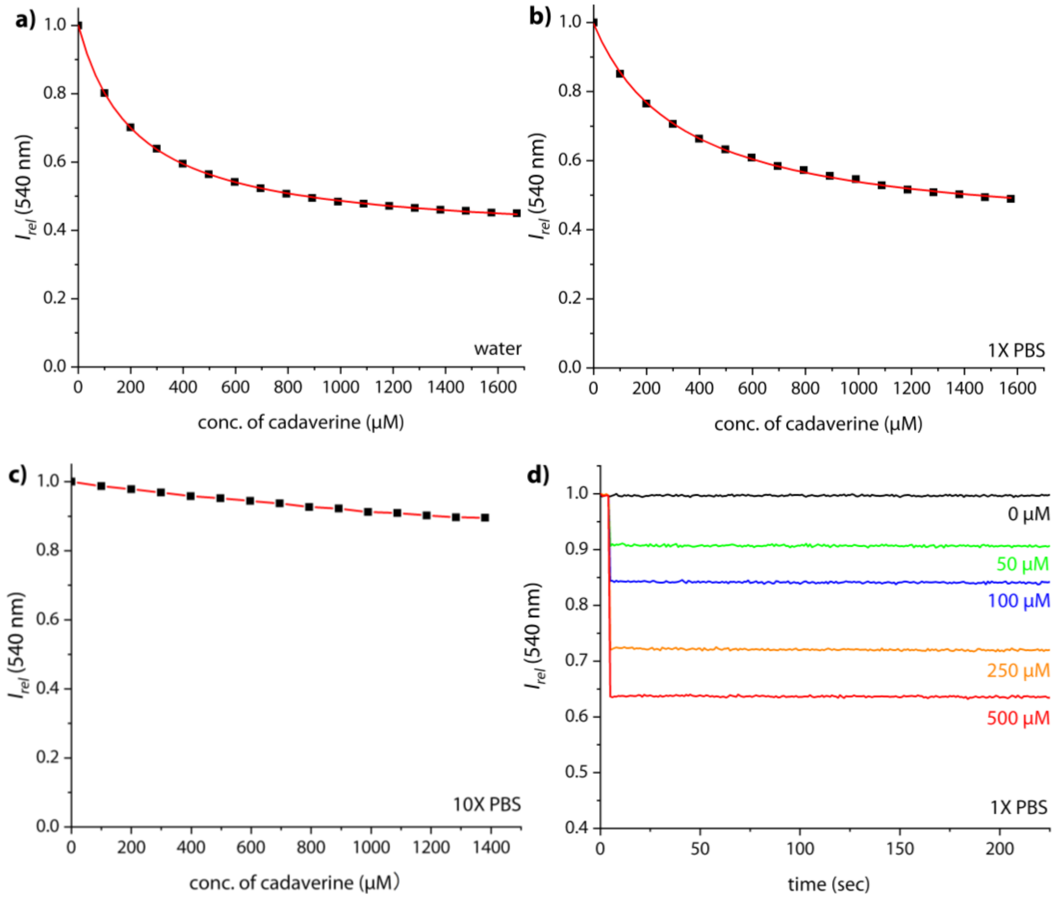

Figure S38. Fitting plot of normalized emission intensity at 540 nm of 1 μM **1** in water (a), 1X PBS (b) and plot of normalized emission intensity at 540 nm of 1 μM **1** in 10X PBS (c) at 25°C, upon addition of cadaverine. Interval time between titration steps: 200 seconds in water, 20 seconds in 1X PBS and 10X PBS. (d) Normalized fluorescence-based kinetic traces at varied concentrations of cadaverine and 1 μM **1** in 1X PBS at 25°C. Concentration of cadaverine for each curve (top to bottom, black to red): 0, 50, 100, 250 and 500 μM, λ_{ex} = 350 nm.

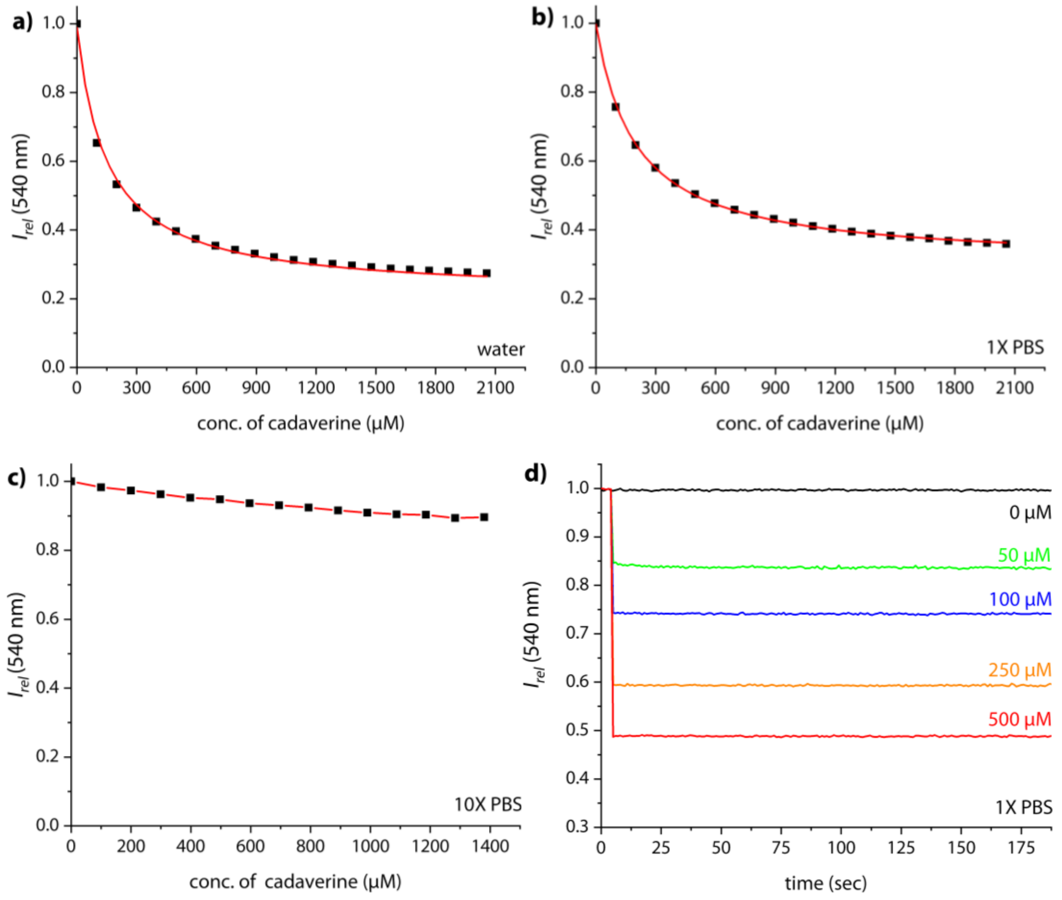

Figure S39. Fitting plot of normalized emission intensity at 540 nm of 1 μM **2** in water (a), 1X PBS (b) and plot of normalized emission intensity at 540 nm of 1 μM **2** in 10X PBS (c) at 25°C, upon addition of cadaverine. Interval time between titration steps: 200 seconds in water, 20 seconds in 1X PBS and 10X PBS. (d) Normalized fluorescence-based kinetic traces at varied concentrations of cadaverine and 1 μM **2** in 1X PBS at 25°C. Concentration of cadaverine for each curve (top to bottom, black to red): 0, 50, 100, 250 and 500 μM, λ_{ex} = 350 nm.

Fluorescence titration of spermine to 1 and 2

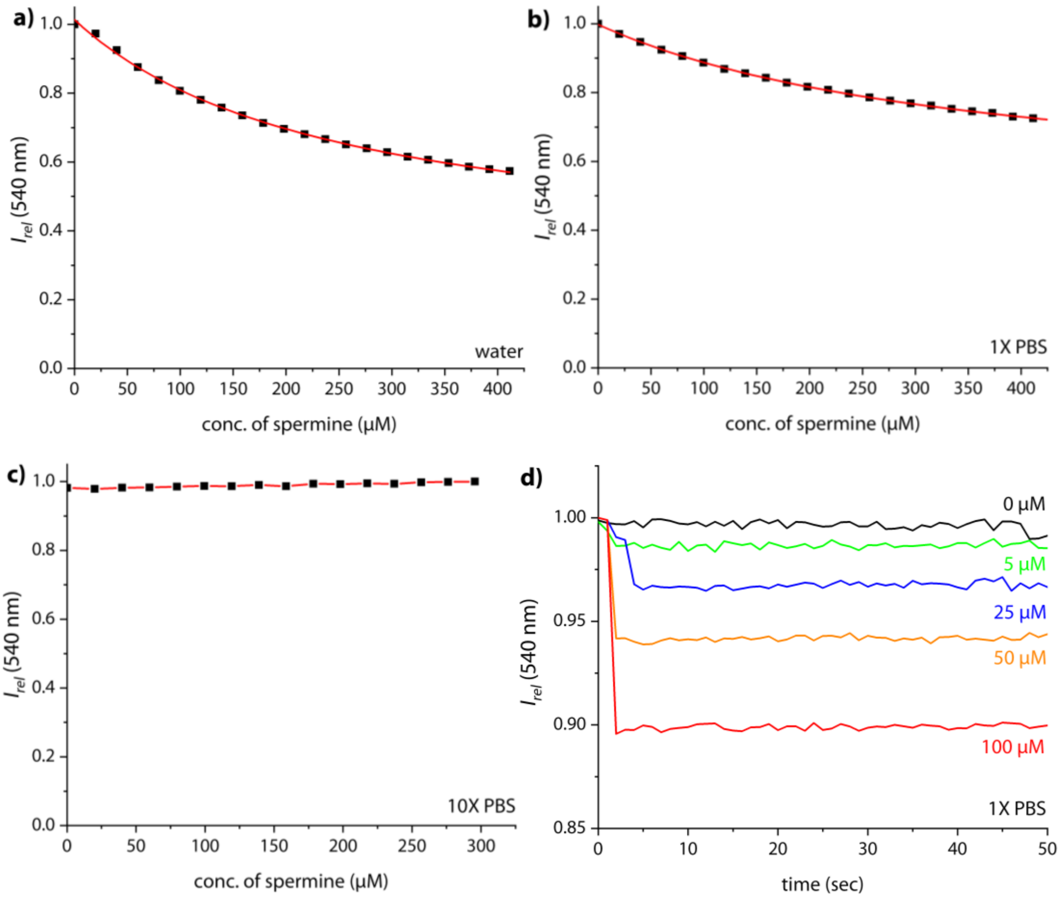

Figure S40. Fitting plot of normalized emission intensity at 540 nm of 1 μM **1** in water (a), 1X PBS (b) and plot of normalized emission intensity at 540 nm of 1 μM **1** in 10X PBS (c) at 25°C, upon addition of spermine. Interval time between titration steps: 200 seconds in water, 20 seconds in 1X PBS and 10X PBS. (d) Normalized fluorescence-based kinetic traces at varied concentrations of spermine and 1 μM **1** in 1X PBS at 25°C. Concentration of spermine for each curve (top to bottom, black to red): 0, 5, 25, 50 and 100 μM, λ_{ex}= 350 nm.

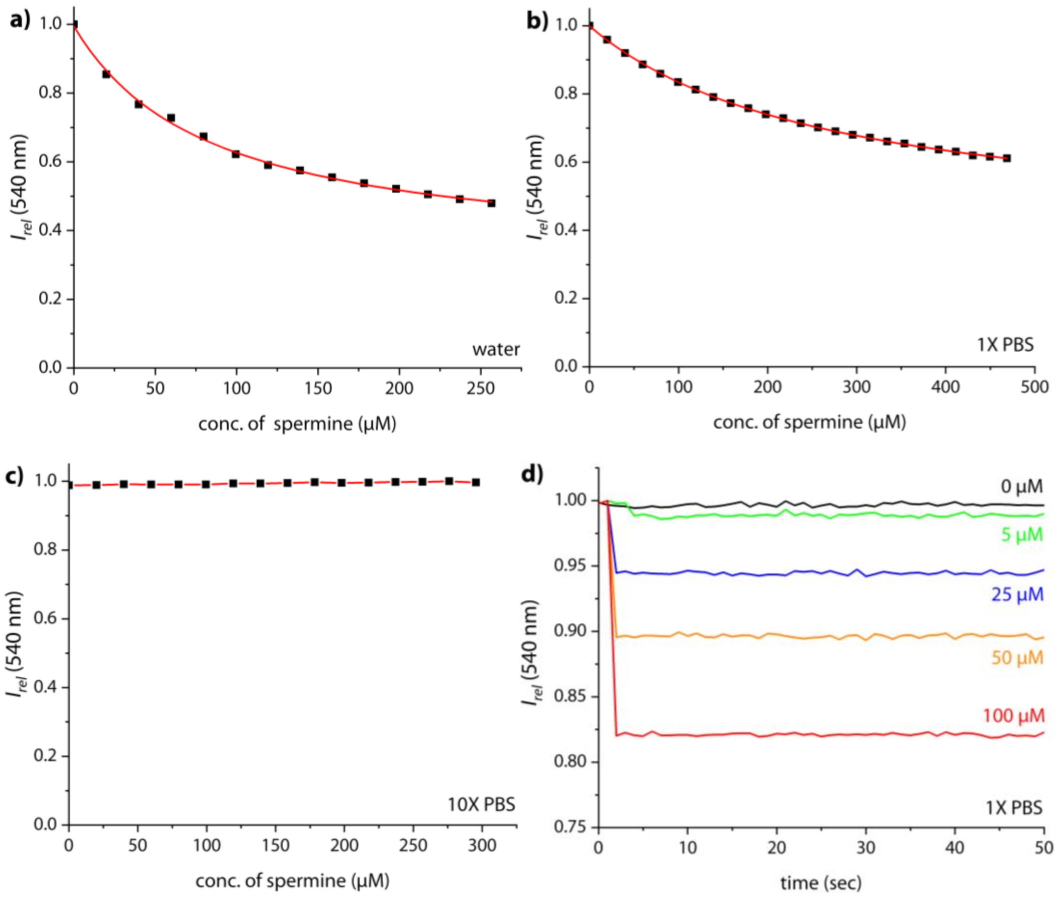

Figure S41. Fitting plot of normalized emission intensity at 540 nm of 1 μM **2** in water (a) ,1X PBS (b) and plot of normalized emission intensity at 540 nm of 1 μM **2** in 10X PBS (c) at 25°C, upon addition of spermine. Interval time between titration steps: 200 seconds in water, 20 seconds in 1X PBS and 10X PBS. (d) Normalized fluorescence-based kinetic traces at varied concentrations of spermine and 1 μM **2** in 1X PBS at 25°C. Concentration of spermine for each curve (top to bottom, black to red): 0, 5, 25, 50 and 75 μM, λ_{ex}= 350 nm.

Fluorescence titration of spermidine to 1 and 2

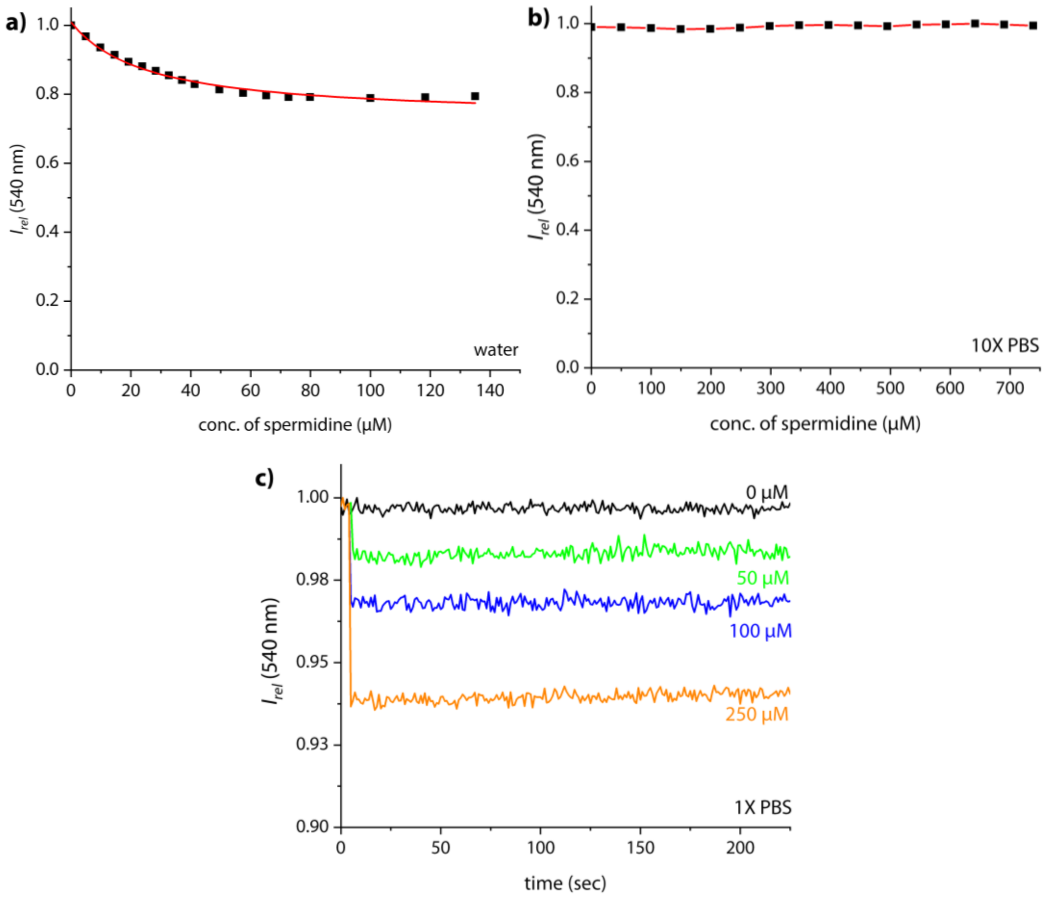

Figure S42. Fitting plot of normalized emission intensity at 540 nm of 1 µM **1** in water (a) and plot of normalized emission intensity at 540 nm of 1 µM **1** in 10X PBS (b) at 25°C, upon addition of spermidine. Interval time between titration steps: 200 seconds in water and 20 seconds in 10X PBS; (c) Normalized fluorescence-based kinetic traces at varied concentrations of spermidine and 1 µM **1** in 1X PBS at 25°C. Concentration of spermidine for each curve (top to bottom, black to orange): 0, 50, 100 and 250 µM, λ_{ex}= 350 nm.

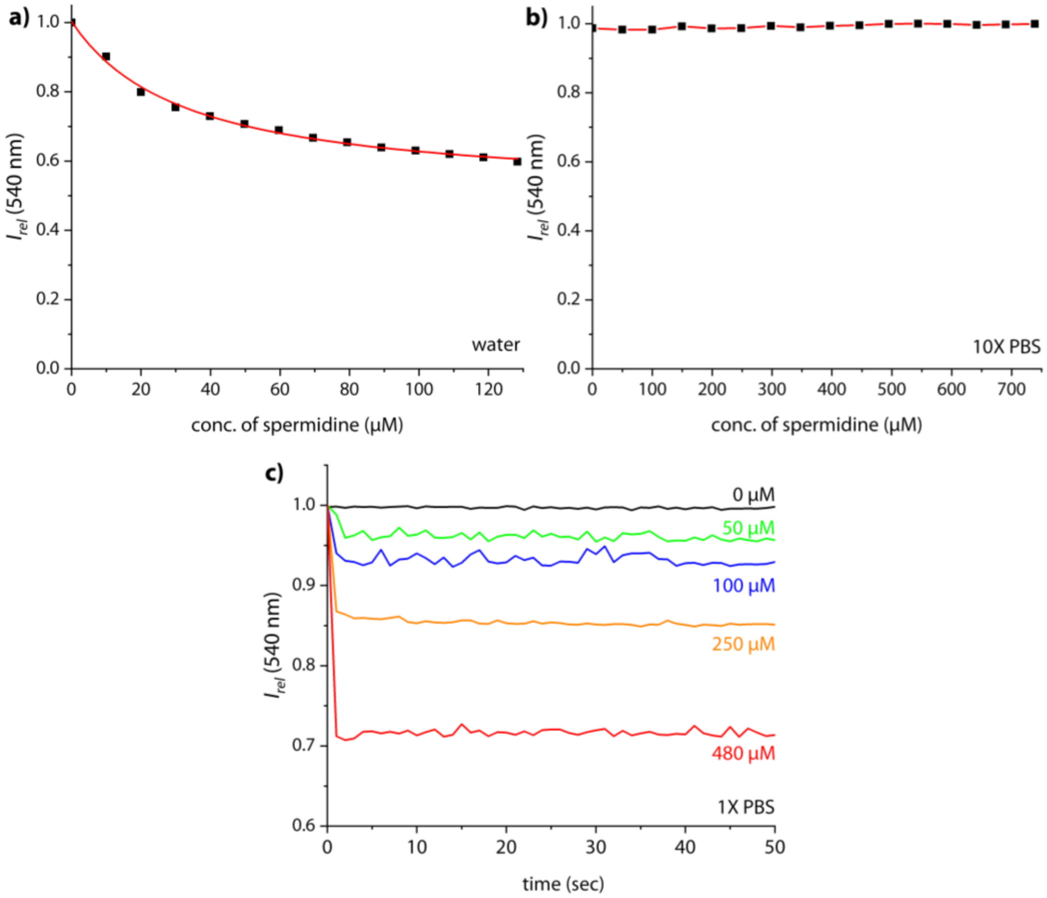

Figure S43. Fitting plot of normalized emission intensity at 540 nm of 1 µM **2** in water (a) and plot of normalized emission intensity at 540 nm of 1 µM **2** in 10X PBS (b) at 25°C, upon addition of spermidine. Interval time between titration steps: 200 seconds in water and 20 seconds in 10X PBS. (c) Normalized fluorescence-based kinetic traces at varied concentrations of spermidine and 1 µM **2** in 1X PBS at 25°C. Concentration of spermidine for each curve (top to bottom, black to red): 0, 50, 100, 250 and 480 µM, λ_{ex}= 350 nm.

Fluorescence titration of 1-adamantanol to 1 and 2

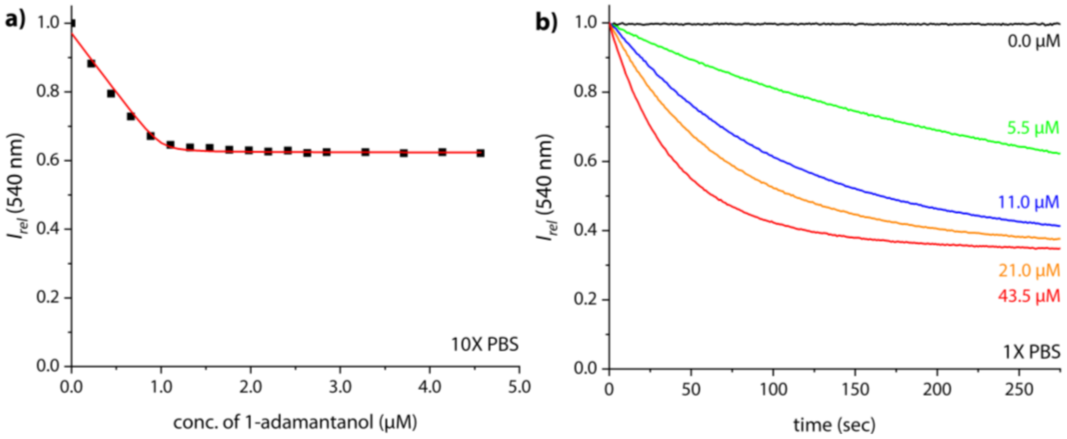

Figure S44. (a) Fitting plot of normalized emission intensity at 540 nm of 1 μM **1** in 10X PBS at 25°C, upon addition of 1-adamantanol. Interval time between titration steps: 3000 seconds in 10X PBS. (b) Normalized fluorescence-based kinetic traces at varied concentrations of 1-adamantanol and 1 μM **1** in 1X PBS at 25°C. Concentration of 1-adamantanol for each curve (top to bottom, black to red): 0.0, 5.5, 11.0, 21.0 and 43.5 μM, λ_{ex}= 350 nm.

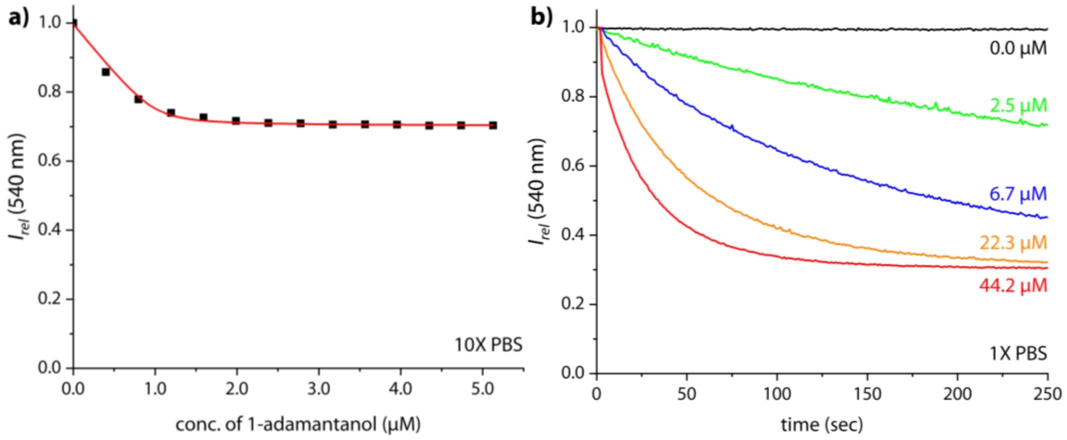

Figure S45. (a) Fitting plot of normalized emission intensity at 540 nm of 1 μM **2** in 10X PBS at 25°C, upon addition of 1-adamantanol. Interval time between titration steps: 3000 seconds in 10X PBS; (b) Normalized fluorescence-based kinetic traces at varied concentrations of 1-adamantanol to 1 μM **2** in 1X PBS at 25°C. Concentration of 1-adamantanol for each curve (top to bottom, black to red): 0.0, 2.5, 6.7, 22.3 and 44.2 μM, λ_{ex}= 350 nm.

Fluorescence titration of nortestosterone to 1 and 2

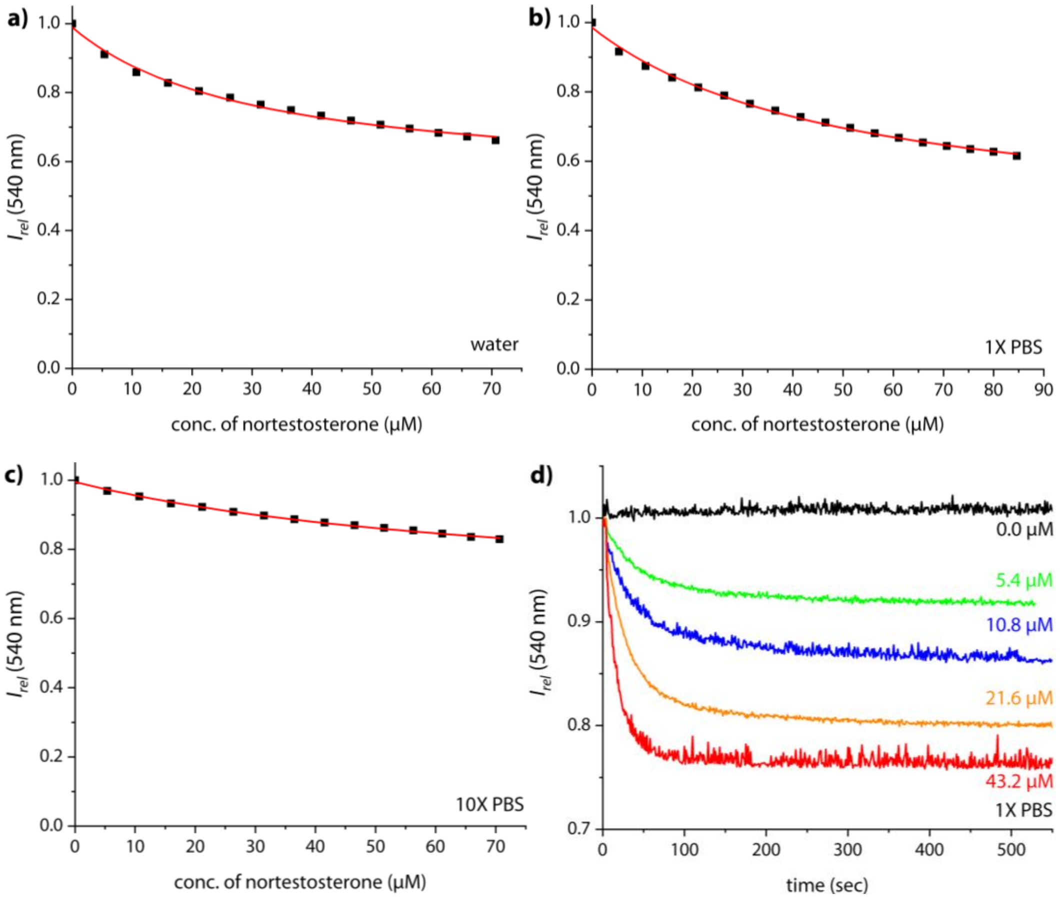

Figure S46. Fitting plot of normalized emission intensity at 540 nm of 1 μM **1** in water (a) ,1X PBS (b) and 10X PBS (c) at 25°C, upon addition of nortestosterone. Interval time between titration steps: 1000 seconds in water, 500 seconds in 1X PBS and 200 seconds in 10X PBS. (d) Normalized fluorescence-based kinetic traces at varied concentrations of nortestosterone and 1 μM **1** in 1X PBS at 25°C. Concentration of nortestosterone for each curve (top to bottom, black to red): 0.0, 5.4, 10.8, 21.6 and 43.2 μM, λ_{ex}= 350 nm.

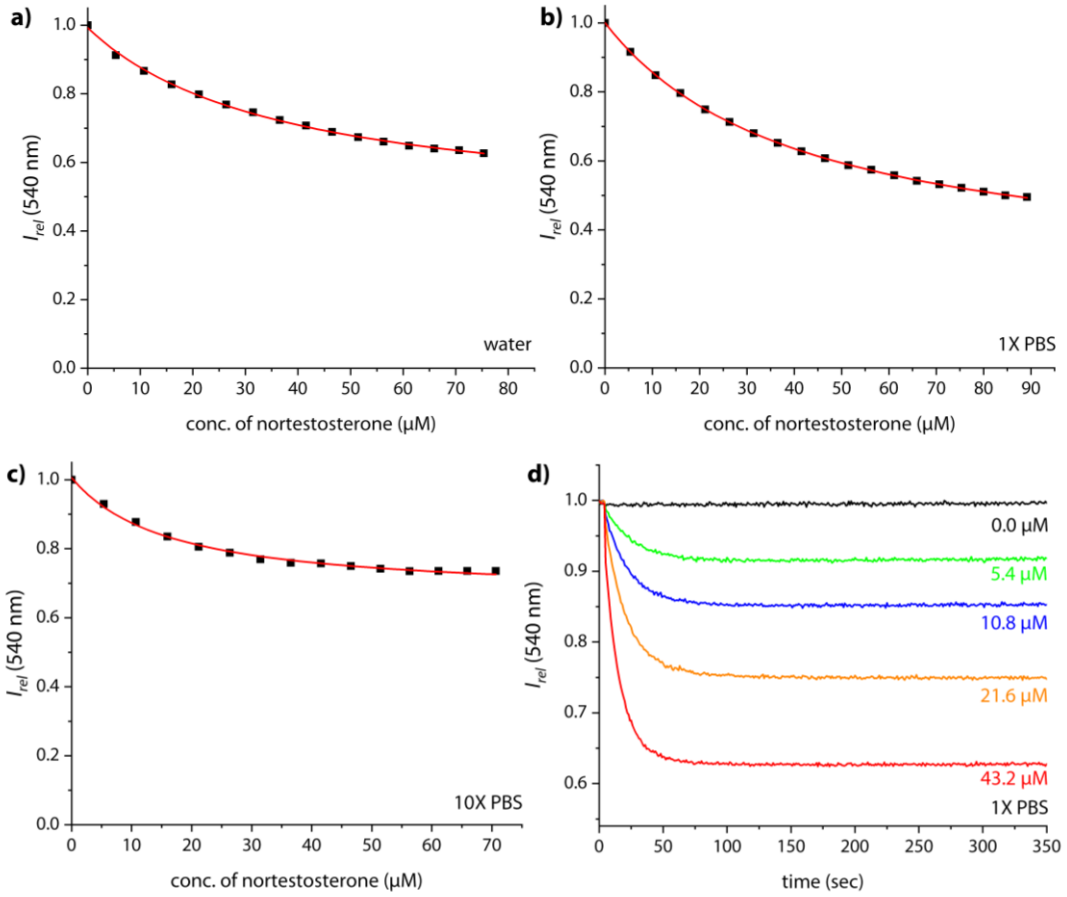

Figure S47. Fitting plot of normalized emission intensity at 540 nm of 1 μM **2** in water (a) ,1X PBS (b) and 10X PBS (c) at 25°C, upon addition of nortestosterone. Interval time between titration steps: 1000 seconds in water, 500 seconds in 1X PBS and 200 seconds in 10X PBS. (d) Normalized fluorescence-based kinetic traces at varied concentrations of nortestosterone and 1 μM **2** in 1X PBS at 25°C. Concentration of nortestosterone for each curve (top to bottom, black to red): 0, 5.4, 10.8, 21.6 and 43.2 μM, λ_{ex}= 350 nm.

Overlay of kinetic traces on addition of amantadine to 1 and 2

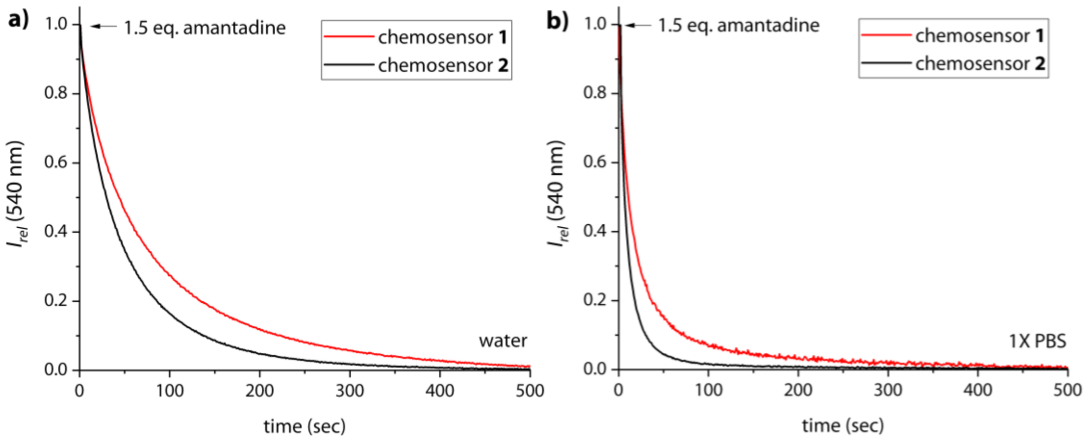

Figure S48. Overlay of normalized fluorescence-based kinetic traces for the addition of 1.5 µM amantadine to 1 µM **1** (red line) and 1 µM **2** (black line) in water (a) and 1X PBS (b) at 25°C, λ_{ex} = 350 nm. (Normalization method [0,1] in Origin software package was used).

Detection limit of 2 for amantadine

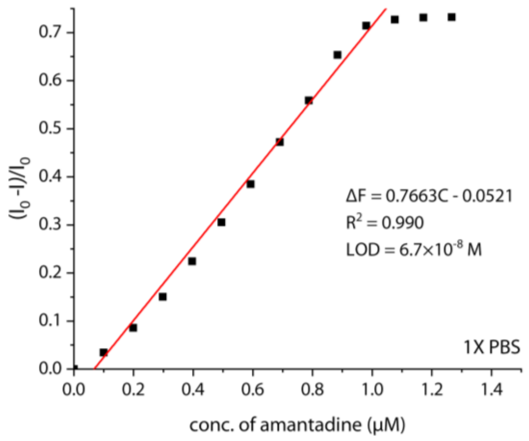

Figure S49. Fitting plot of emission intensity monitored at 540 nm of 1 µM **2** in 1X PBS at 25°C, upon addition of amantadine, λ_{ex} = 350 nm. The emission intensity of **2** without any amantadine was measured for five individual samples in 1X PBS and based on these measurements the standard deviation (σ) was determined. The limit of detection (LOD) was then calculated with the equation, 3σ/S. S is the slope between intensity versus concentration of amantadine (ΔF is the ratio of fluorescence intensity change, and C is the concentration of amantadine in µM). Detection of 100 nM amantadine in surine is shown in Figure S53.

Amantadine determination in biofluid probes

Amantadine determination with 1 and 2 in surine

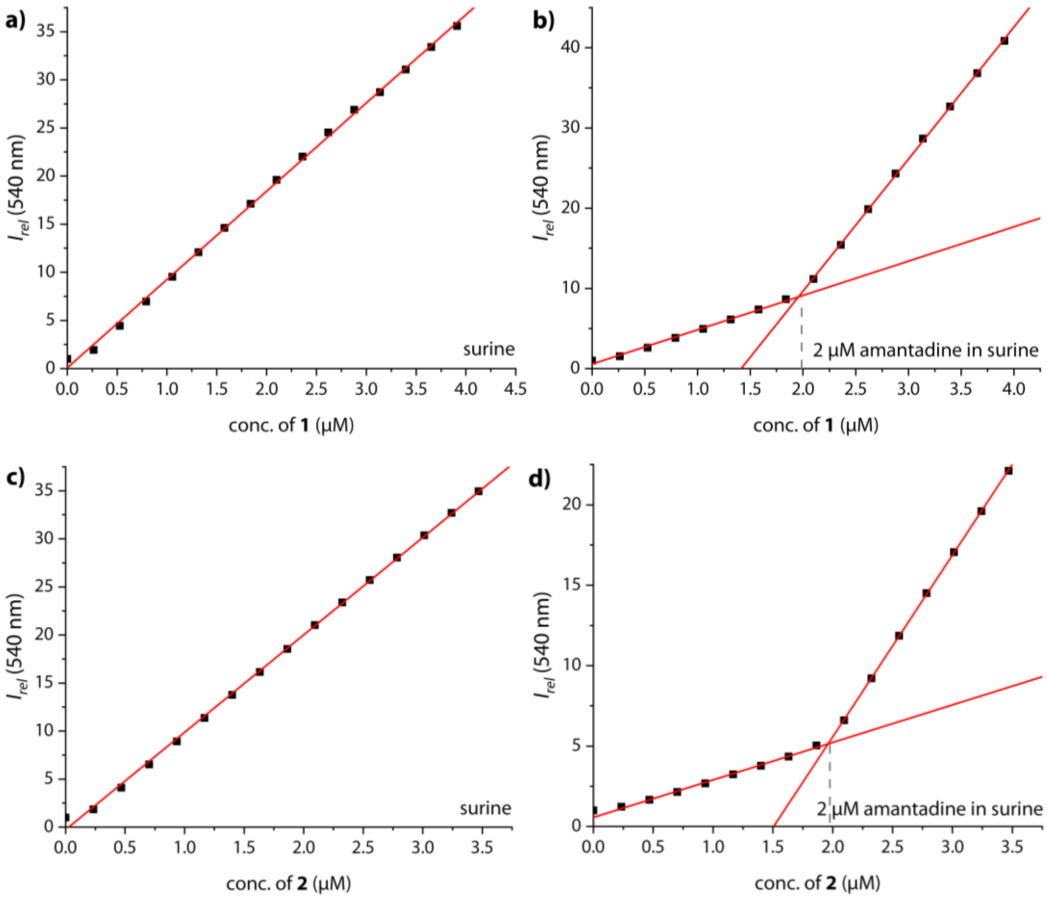

Figure S50. Fitting plot of the normalized emission intensity at 540 nm versus concentration of **1** (a) and **2** (c) in surine without any amantadine, and concentration of **1** (b) and **2** (d) in surine spiked with 2 μM amantadine at 25°C, λ_{ex} = 350 nm. Interval time between titration steps: 50 seconds.

Amantadine determination with CB7⊃MDAP in surine

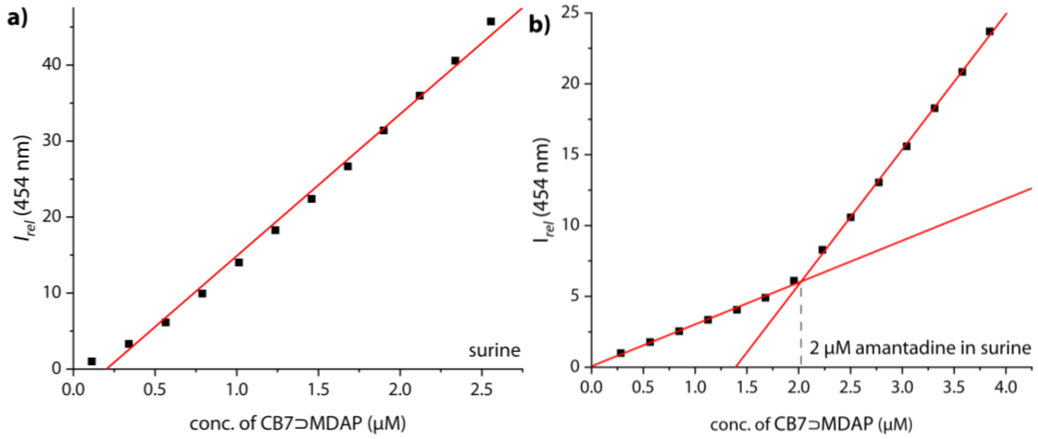

Figure S51. Fitting plot of the normalized emission intensity at 454 nm versus concentration of CB7⊃MDAP (a) in surine without any amantadine and (b) surine spiked with 2 μM amantadine at 25°C, λ_{ex} = 339 nm. Interval time between titration steps: 200 seconds.

Amantadine determination with 2 and CB7⊃MDAP in surine in the presence of spermine

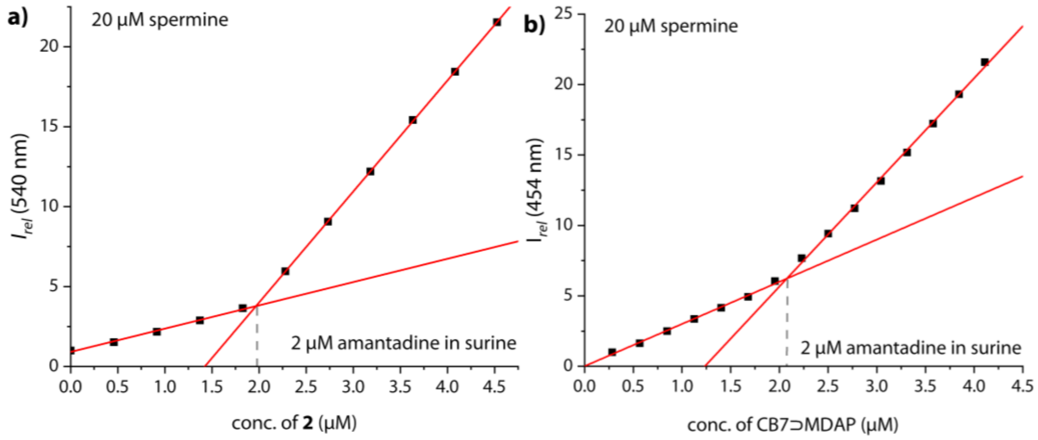

Figure S52. Fitting plot of the normalized emission intensity versus concentration of (a) **2** at 540 nm, λ_{ex} = 350 nm and (b) CB7⊃MDAP at 454 nm, λ_{ex} = 339 nm in surine spiked with 2 μM amantadine and 20 μM spermine at 25°C. Interval time between titration steps: 50 seconds for **2** and 200 seconds for CB7⊃MDAP.

100 nM amantadine detection by 2 in surine

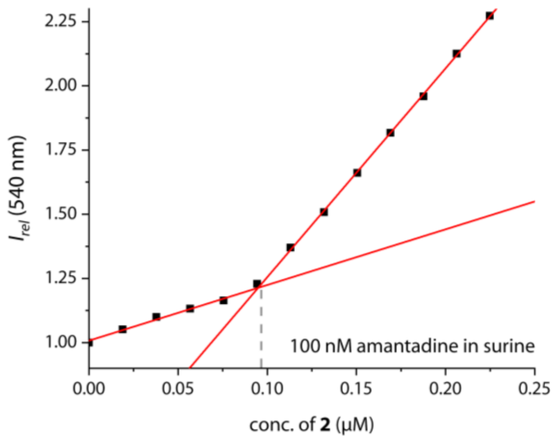

Figure S53. Fitting plot of the normalized emission intensity at 540 nm versus concentration of **2** in surine spiked with 100 nM amantadine at 25°C, λ_{ex} = 350 nm. Interval time between titration steps: 200 seconds.

Amantadine determination with 1 and 2 in urine

In order to simulate urine from Parkinson patients receiving amantadine treatment, urine samples from three healthy donors were each spiked with 10 µM amantadine and then diluted with water to reach a final concentration of 2 µM amantadine following the biological relevant concentration. The urine was probed without any processing steps. It was used within 3 days after excretion and stored in the fridge. To probe different urine compositions, morning urine and daily urine samples were used, however, the higher concentration of ingredients in the morning urine didn't affect the sensors at all. The fitting curves were obtained through a linear fit function by using the software Origin.

Urine sample I

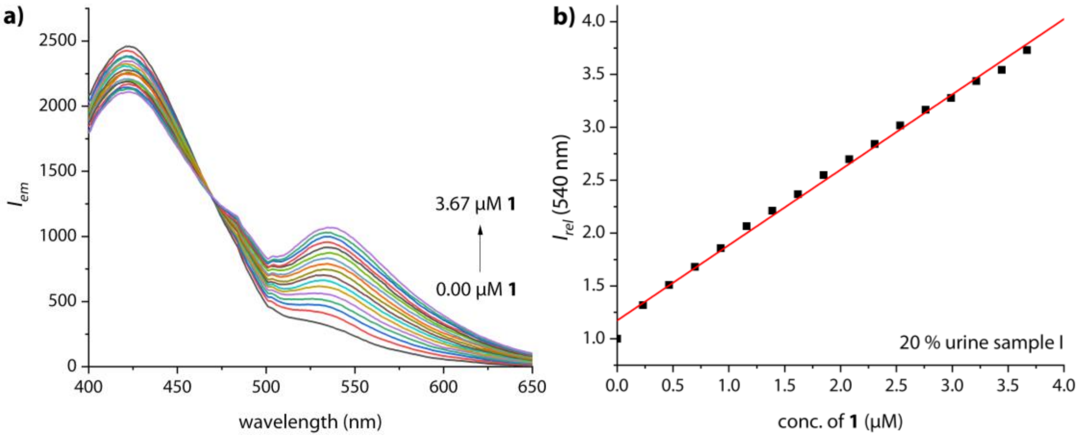

Figure S54. (a) Emission spectra of 20% diluted urine sample I without any amantadine at 25°C, upon addition of **1** (0 – 3.67 µM); b) Fitting plot of the normalized emission intensity at 540 nm versus concentration of **1** in 20% diluted urine sample I at 25°C, λ_{ex} = 350 nm. Interval time between titration steps: 50 seconds.

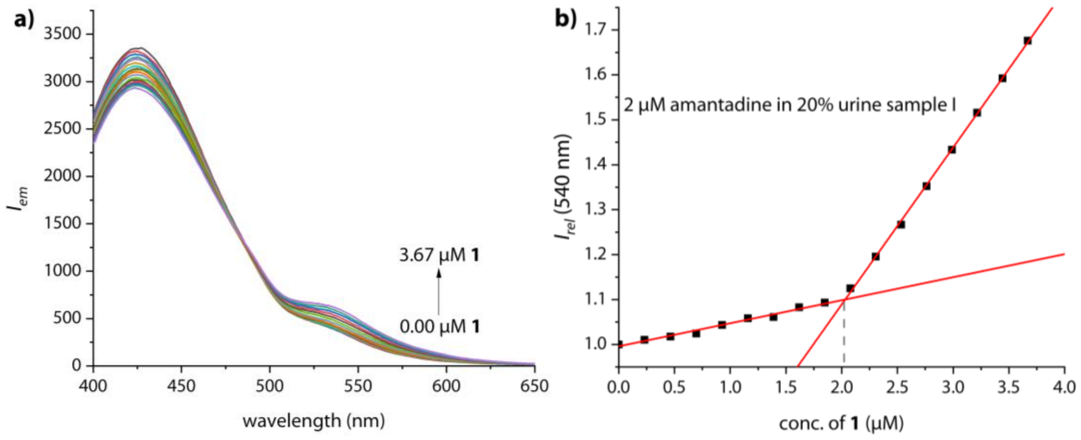

Figure S55. (a) Emission spectra of 20% diluted urine sample I spiked with 2 µM amantadine (end concentration) at 25°C, upon addition of **1** (0 – 3.67 µM); (b) Fitting plot of the normalized emission intensity at 540 nm versus concentration of **1** in 20% diluted urine sample I containing 2 µM amantadine at 25°C, λ_{ex} = 350 nm. Interval time between titration steps: 50 seconds.

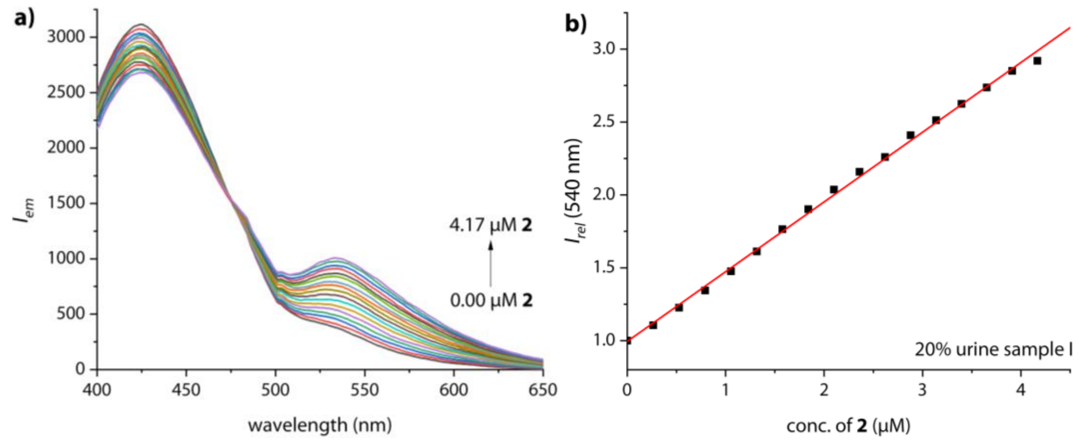

Figure S56. (a) Emission spectra of 20% diluted urine sample I without any amantadine at 25°C upon addition of **2** (0 – 4.17 µM); (b) Fitting plot of the normalized emission intensity at 540 nm versus concentration of **2** in 20% diluted urine sample I at 25°C, λ_{ex} = 350 nm. Interval time between titration steps: 50 seconds.

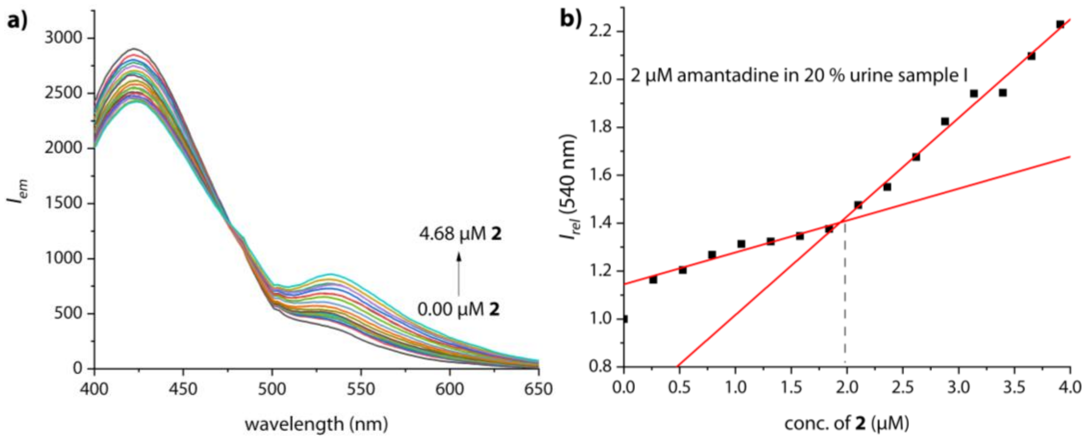

Figure S57. (a) Emission spectra of 20% diluted urine sample I spiked with 2 µM amantadine (end concentration) at 25°C, upon addition of **2** (0 – 4.68 µM); (b) Fitting plot of the normalized emission intensity at 540 nm versus concentration of **2** in 20% diluted urine sample I containing 2 µM amantadine at 25°C, λ_{ex} = 350 nm. Interval time between titration steps: 50 seconds.

Urine sample II

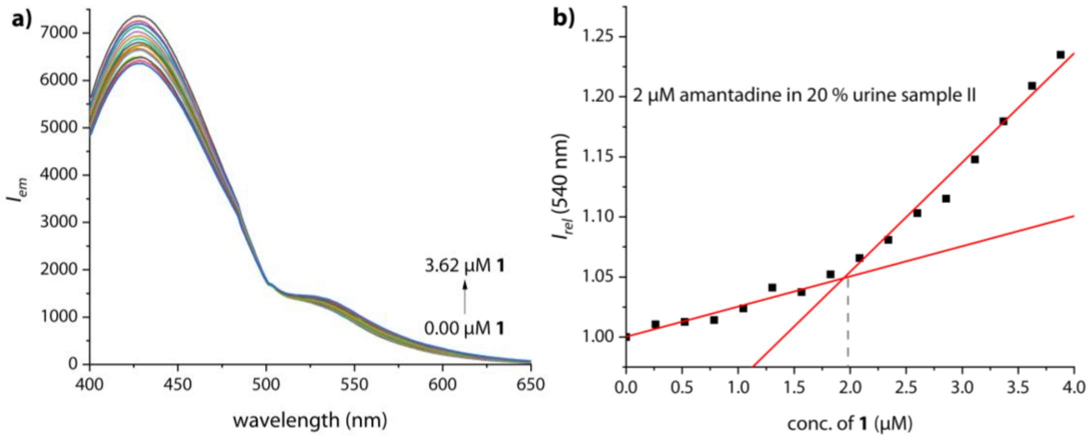

Figure S58. (a) Emission spectra of 20% diluted urine sample II spiked with 2 μM amantadine (end concentration) at 25°C, upon addition of **1** (0 – 3.62 μM); (b) Fitting plot of the normalized emission intensity at 540 nm versus concentration of **1** in 20% diluted urine sample II containing 2 μM amantadine at 25°C, λ_{ex} = 350 nm. Interval time between titration steps: 50 seconds.

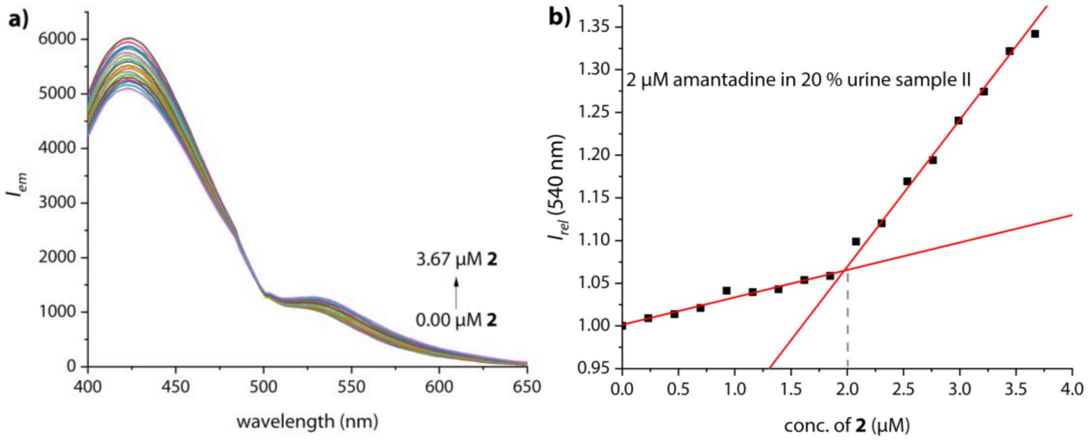

Figure S59. (a) Emission spectra of 20% diluted urine sample II spiked with 2 μM amantadine (end concentration) at 25°C, upon addition of **2** (0 – 3.67 μM); (b) Fitting plot of the normalized emission intensity at 540 nm versus concentration of **2** in 20% diluted urine sample II containing 2 μM amantadine at 25°C, λ_{ex} = 350 nm. Interval time between titration steps: 50 seconds.

Urine sample III

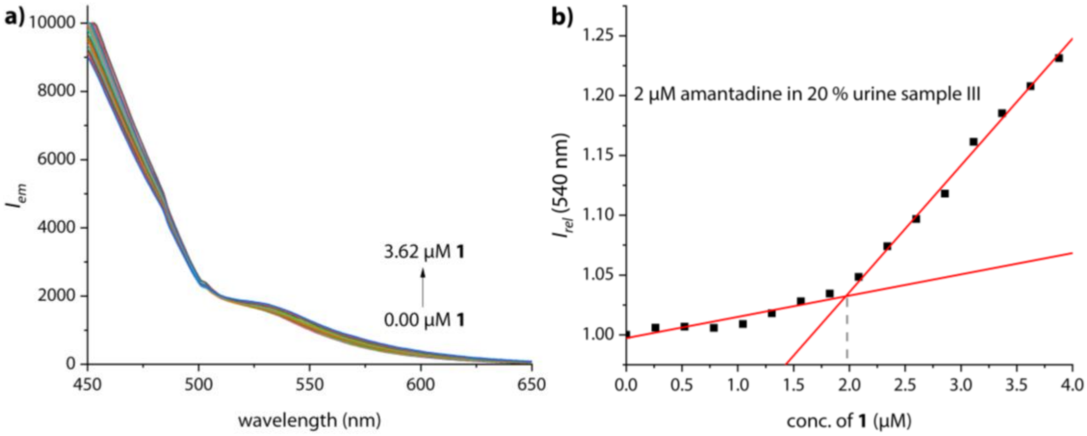

Figure S60. (a) Emission spectra of 20% diluted urine sample III spiked with 2 μM amantadine (end concentration) at 25°C, upon addition of **1** (0 – 3.62 μM); (b) Fitting plot of the normalized emission intensity at 540 nm versus concentration of **1** in 20% diluted urine sample III containing 2 μM amantadine at 25°C, λ_{ex} = 350 nm. Interval time between titration steps: 50 seconds.

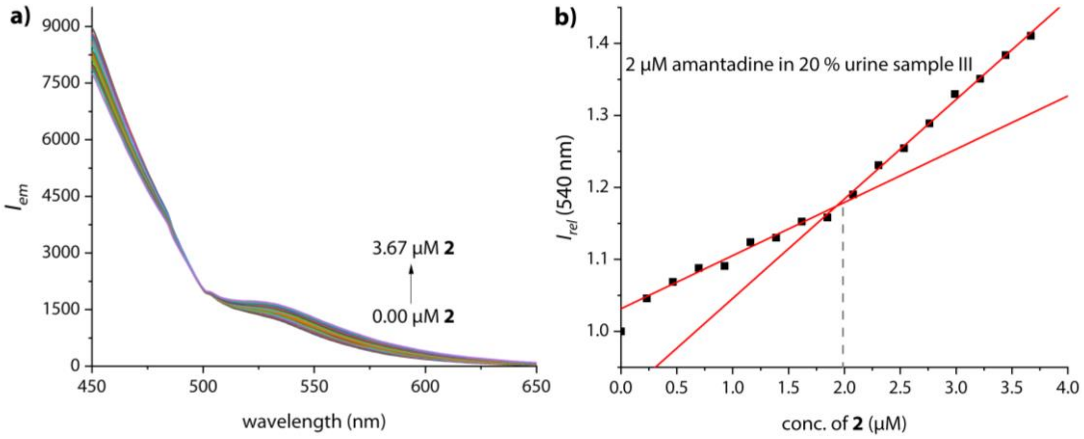

Figure S61. (a) Emission spectra of 20% diluted urine sample III spiked with 2 μM amantadine (end concentration) at 25°C, upon addition of **2** (0 – 3.67 μM); (b) Fitting plot of the normalized emission intensity at 540 nm versus concentration of **2** in 20% diluted urine sample III containing 2 μM amantadine at 25°C, λ_{ex} = 350 nm. Interval time between titration steps: 50 seconds.

Amantadine determination with 1 and 2 in saliva

In order to simulate saliva from Parkinson patients receiving amantadine treatment, saliva samples from three healthy donors were each spiked with 2 µM amantadine following the biological relevant concentration. The saliva was probed after centrifuging at 8000 rpm for 10 minutes to remove insoluble component. It was used within 3 days after excretion and stored in the fridge. The fitting curves were obtained through a linear fit function by using the software Origin.

Saliva sample I

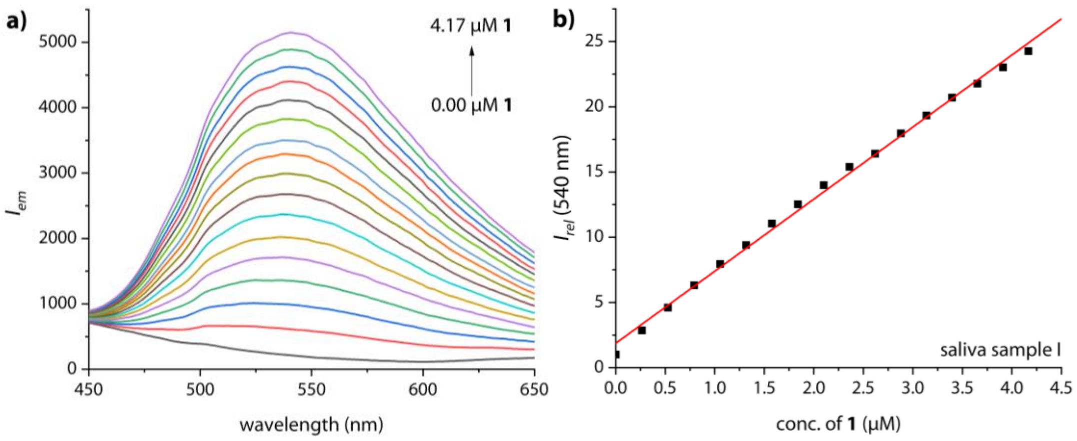

Figure S62. (a) Emission spectra of saliva sample I without any amantadine at 25°C, upon addition of **1** (0 – 4.17 µM); b) Fitting plot of the normalized emission intensity at 540 nm versus concentration of **1** in saliva sample I at 25°C, λ_{ex} = 350 nm.

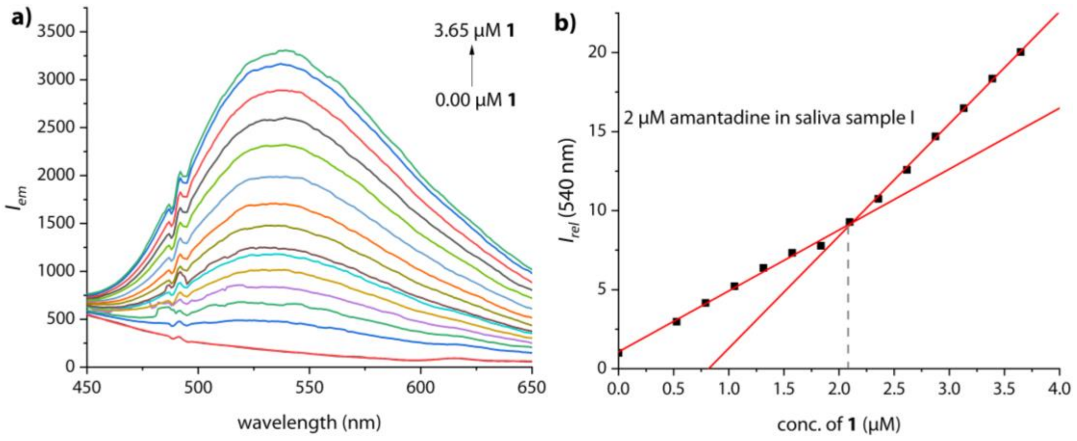

Figure S63. (a) Emission spectra of saliva sample I spiked with 2 µM amantadine (end concentration) at 25°C, upon addition of **1** (0 – 3.65 µM); (b) Fitting plot of the normalized emission intensity at 540 nm versus concentration of **1** in saliva sample I containing 2 µM amantadine at 25°C, λ_{ex} = 350 nm.

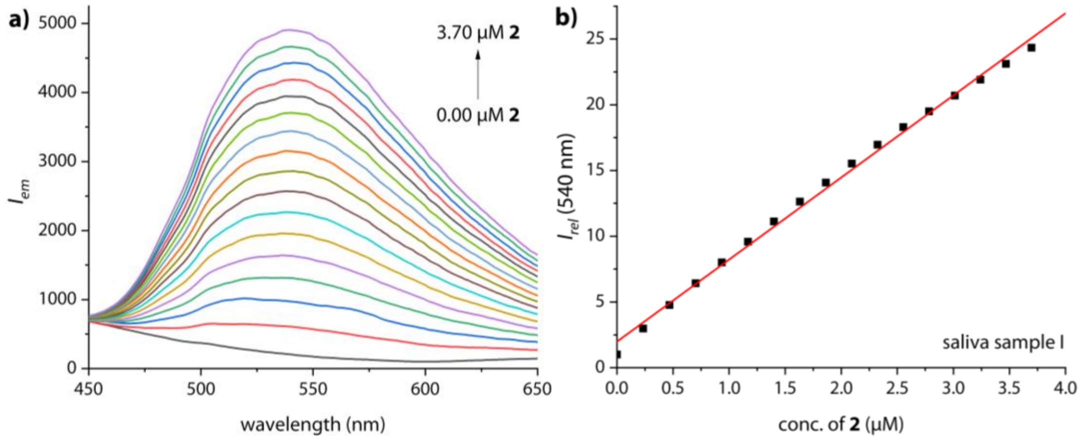

Figure S64. (a) Emission spectra of saliva sample I without any amantadine at 25°C, upon addition of **2** (0 – 3.70 μM), λ_{ex} = 350 nm; b) Fitting plot of the normalized emission intensity at 540 nm versus concentration of **2** in saliva sample I at 25°C, λ_{ex} = 350 nm.

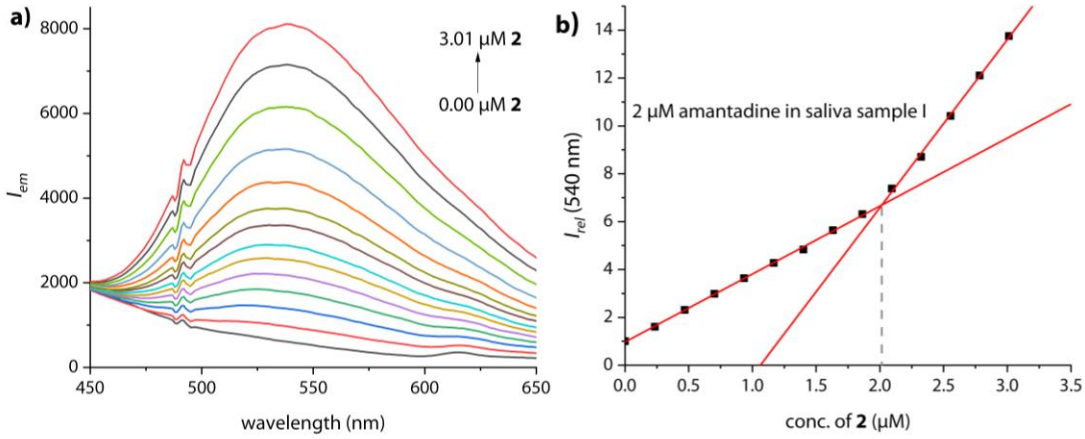

Figure S65. (a) Emission spectra of saliva sample I spiked with 2 μM amantadine (end concentration) at 25°C, upon addition of **2** (0 – 3.01 μM); b) Fitting plot of the normalized emission intensity at 540 nm versus concentration of **2** in saliva sample I containing 2 μM amantadine at 25°C, λ_{ex} = 350 nm.

Saliva sample II

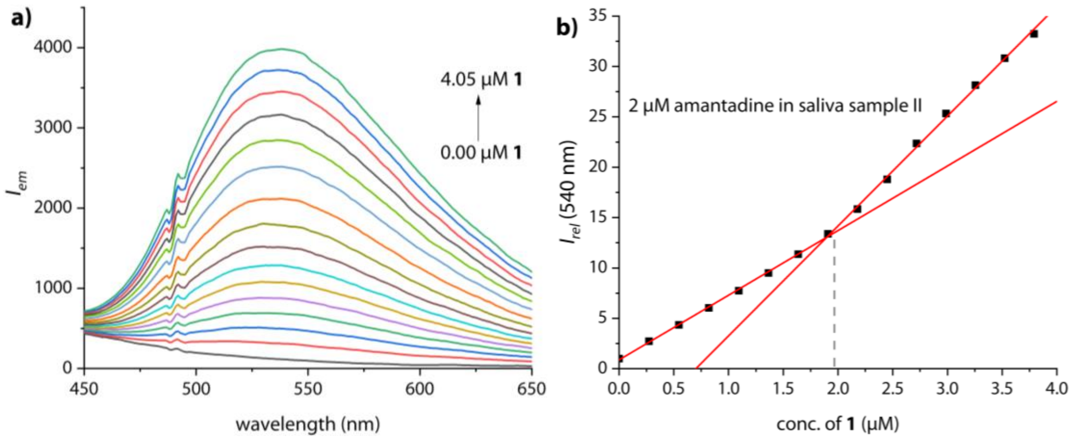

Figure S66. (a) Emission spectra of saliva sample II spiked with 2 μM amantadine (end concentration) at 25°C, upon addition of **1** (0 – 4.05 μM); b) Fitting plot of the normalized emission intensity at 540 nm versus concentration of **1** in saliva sample II containing 2 μM amantadine at 25°C, λ_{ex} = 350 nm.

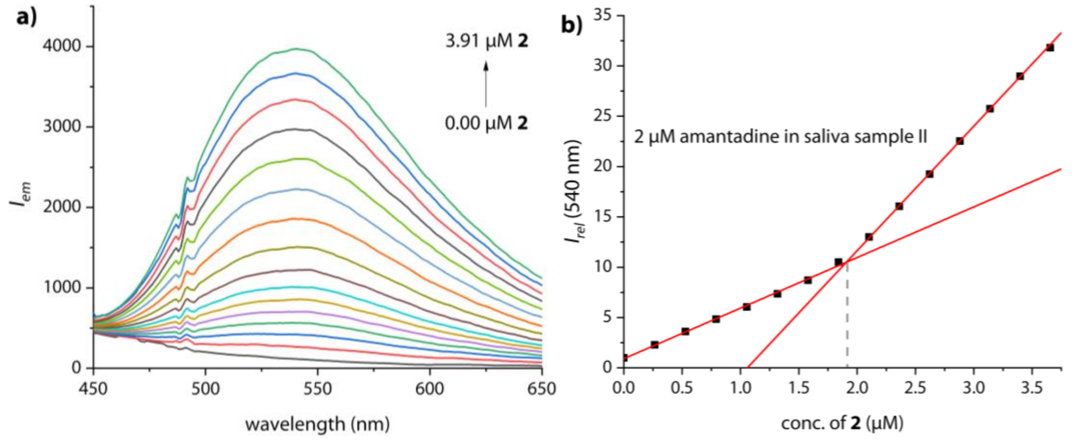

Figure S67. (a) Emission spectra of saliva sample II spiked with 2 μM amantadine (end concentration) at 25°C, upon addition of **2** (0 – 3.91 μM); b) Fitting plot of the normalized emission intensity at 540 nm versus concentration of **2** in saliva sample II containing 2 μM amantadine at 25°C, λ_{ex} = 350 nm.

Saliva sample III

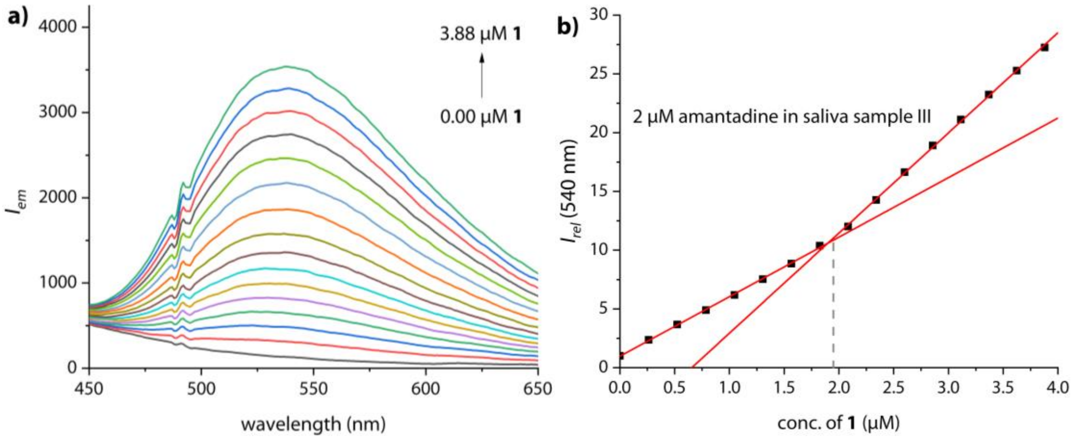

Figure S68. (a) Emission spectra of saliva sample III spiked with 2 μM amantadine (end concentration) at 25°C, upon addition of **1** (0 – 3.88 μM); b) Fitting plot of the normalized emission intensity at 540 nm versus concentration of **1** in saliva sample III containing 2 μM amantadine at 25°C, λ_{ex} = 350 nm.

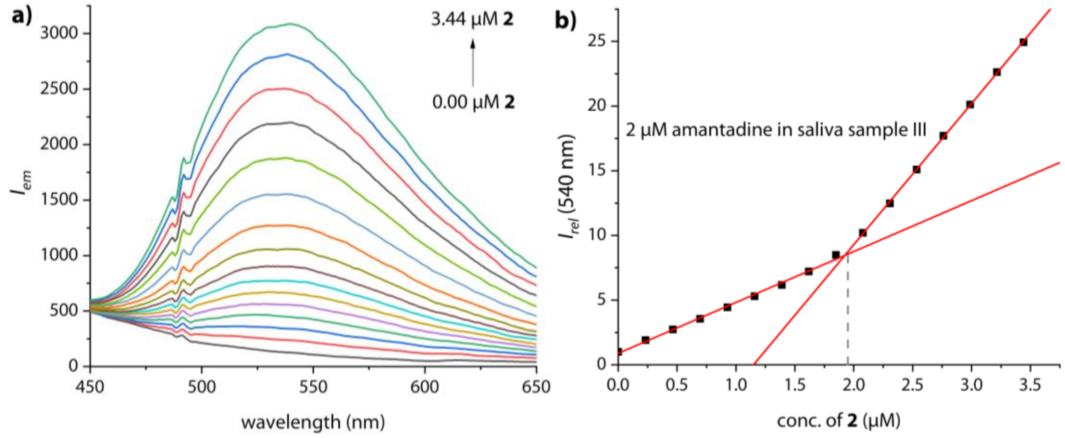

Figure S69. (a) Emission spectra of saliva sample III spiked with 2 μM amantadine (end concentration) at 25°C, upon addition of **2** (0 – 3.44 μM); b) Fitting plot of the normalized emission intensity at 540 nm versus concentration of **2** in saliva sample III containing 2 μM amantadine at 25°C, λ_{ex} = 350 nm.

Amantadine determination with 2 and CB7⊃MDAP in artificial saliva in the absence or presence of cadaverine

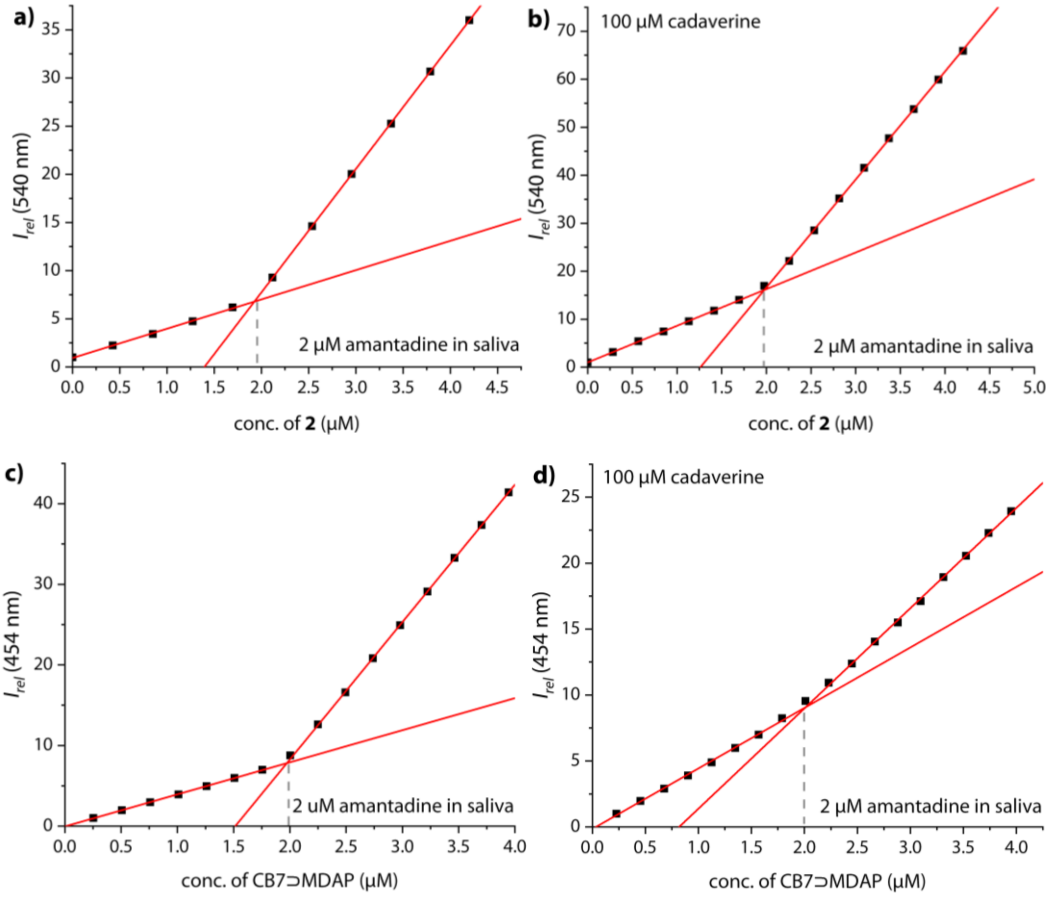

Figure S70. Fitting plot of the normalized emission intensity versus concentration of **2** at 540 nm, λ_{ex} = 350 nm in artificial saliva spiked with 2 μM amantadine (a) and additional 100 μM cadaverine (b) at 25°C; Fitting plot of the normalized emission intensity versus concentration of CB7⊃MDAP at 454 nm, λ_{ex} = 339 nm in artificial saliva spiked with 2 μM amantadine (c) and additional 100 μM cadaverine (d) at 25°C. Interval time between titration steps: 50 seconds for **2** and 200 seconds for CB7⊃MDAP.

References

1 a) C. Marquez, H. Fang and W. M. Nau, *IEEE Trans. Nanobiosci.*, 2004, **3**, 39; b) D. Jiao and O. A. Scherman, *Green Chem.*, 2012, **14**, 2445.

2 N. Dong, J. He, T. Li, A. Peralta, M. R. Avei, M. Ma and A. E. Kaifer, *J. Org. Chem.*, 2018, **83**, 5467.

3 S. Zhang, Z. Domínguez, K. I. Assaf, M. Nilam, T. Thiele, U. Pischel, U. Schedler, W. M. Nau and A. Hennig, *Chem. Sci.*, 2018, **9**, 8575.

4 M. A. Cejas and F. M. Raymo, *Langmuir*, 2005, **21**, 5795.

5 H. Cao, S. Liao, W. Zhong, X. Xiao, J. Zhu, W. Li, X. Wu and Y. Feng, *Molecules*, 2017, **22**, 1752.

6 T.-H. Chen, A. Schneemann, R. A. Fischer and S. M. Cohen, *Dalton Trans.*, 2016, **45**, 3063.

7 V. A. Shadrikova, E. V. Golovin and Y. N. Klimochkin, *Chem. Heterocycl. Compd.*, 2015, **50**, 1586.
